# Cryo-EM Structures of Eastern Equine Encephalitis Virus Reveal Mechanisms of Virus Disassembly and Antibody Neutralization

**DOI:** 10.1016/j.celrep.2018.11.067

**Published:** 2018-12-11

**Authors:** S. Saif Hasan, Chengqun Sun, Arthur S. Kim, Yasunori Watanabe, Chun-Liang Chen, Thomas Klose, Geeta Buda, Max Crispin, Michael S. Diamond, William B. Klimstra, Michael G. Rossmann

**Affiliations:** 1 Department of Biological Sciences, Purdue University, West Lafayette, IN 47907, USA; 2 Department of Immunology, University of Pittsburgh, Pittsburgh, PA 15261, USA; 3 Center for Vaccine Research, University of Pittsburgh, Pittsburgh, PA 15261, USA; 4 Department of Medicine, Washington University School of Medicine, St. Louis, MO 63110, USA; 5 Department of Pathology and Immunology, Washington University School of Medicine, St. Louis, MO 63110, USA; 6 Centre for Biological Sciences and Institute of Life Sciences, University of Southampton, Southampton SO17 1BJ, UK; 7 Oxford Glycobiology Institute, Department of Biochemistry, University of Oxford, Oxford OX1 3QU, UK; 8 Division of Structural Biology, University of Oxford, Oxford OX3 7BN, UK; 9 Department of Molecular Microbiology, Washington University School of Medicine, St. Louis, MO 63110, USA; 10 The Andrew M. and Jane M. Bursky Center for Human Immunology and Immunotherapy Programs, Washington University School of Medicine, St. Louis, MO 63110, USA

**Keywords:** alphavirus, cryoelectron microscopy, glycosylation, antibodies, conformational changes, virus entry, virus disassembly

## Abstract

Alphaviruses are enveloped pathogens that cause arthritis and encephalitis. Here, we report a 4.4-Å cryoelectron microscopy (cryo-EM) structure of eastern equine encephalitis virus (EEEV), an alphavirus that causes fatal encephalitis in humans. Our analysis provides insights into viral entry into host cells. The envelope protein E2 showed a binding site for the cellular attachment factor heparan sulfate. The presence of a cryptic E2 glycan suggests how EEEV escapes surveillance by lectin-expressing myeloid lineage cells, which are sentinels of the immune system. A mechanism for nucleocapsid core release and disassembly upon viral entry was inferred based on pH changes and capsid dissociation from envelope proteins. The EEEV capsid structure showed a viral RNA genome binding site adjacent to a ribosome binding site for viral genome translation following genome release. Using five Fab-EEEV complexes derived from neutralizing antibodies, our investigation provides insights into EEEV host cell interactions and protective epitopes relevant to vaccine design.

## Highlights

• EEEV cryo-EM structure shows the basis of receptor binding and pH-triggered disassembly
• Cryptic envelope protein glycosylation interferes with immune detection
• EEEV RNA genome binding site on capsid protein has an extended conformation
• Antibody inhibition of EEEV entry involves cross-linking of viral envelope proteins

## Graphical Abstract

**Figure:**
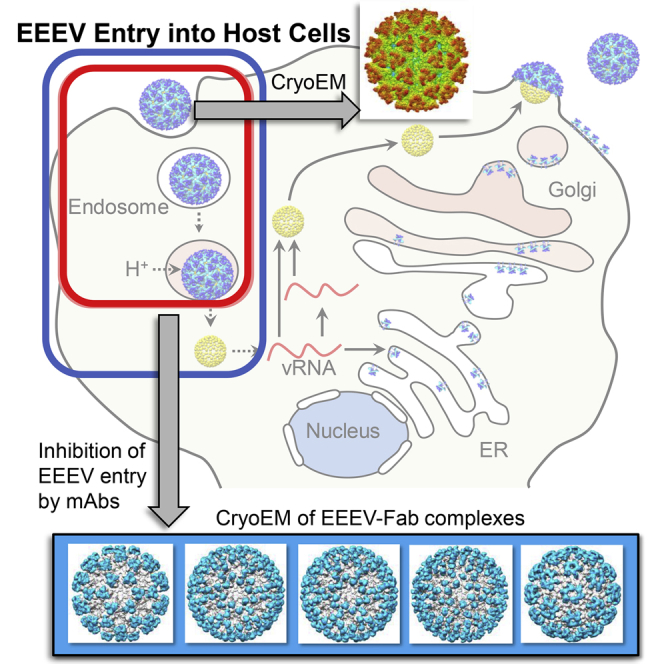

## Teaser

Hasan et al. use single-particle cryoelectron microscopy to elucidate the molecular basis of host cell entry of neurovirulent EEEV. They show that the EEEV envelope is primed for intracellular pH sensing and subsequent disassembly. Monoclonal antibodies effectively inhibit EEEV entry by cross-linking the viral envelope.

## Introduction

Alphaviruses are arthropod-transmitted enveloped pathogens that cause epidemics in humans and other vertebrate animals (Jose et al., 2009, Schwartz and Albert, 2010, Strauss and Strauss, 1994). Alphaviruses have an ∼12-kb unsegmented single-stranded (+)RNA genome that encodes four non-structural and five structural proteins (Strauss and Strauss, 1994). The icosahedral shell of alphaviruses consists of an outer layer of trans-membrane envelope E1 and E2 proteins and an inner capsid layer separated by a host-derived membrane. Previous cryoelectron microscopy (cryo-EM) studies of chikungunya (CHIKV), Semliki Forest (SFV), Sindbis (SINV), Ross River (RRV), Venezuelan (VEEV), and western equine encephalitis (WEEV) viruses have shown that the E1 and E2 proteins are organized into 20 icosahedral 3-fold and 60 quasi-3-fold trimeric spikes (Kostyuchenko et al., 2011, Mancini et al., 2000, Mukhopadhyay et al., 2006, Sherman and Weaver, 2010, Smith et al., 1995, Sun et al., 2013, Zhang et al., 2002, Zhang et al., 2005, Zhang et al., 2011). Crystallographic structures of the E1 and E2 ectodomains and the capsid C-terminal domain (CTD) also have been determined for several alphaviruses (Choi et al., 1991, Gibbons et al., 2004, Lescar et al., 2001, Li et al., 2010, Voss et al., 2010). The capsid N-terminal domain (NTD) is disordered and binds the negatively charged alphavirus RNA genome (Owen and Kuhn, 1996).

Alphaviruses utilize the E2 protein for attachment to incompletely characterized receptors (Schwartz and Albert, 2010, Zhang et al., 2018). Alphaviruses are internalized by endocytosis (Figure S1). Endosome acidification triggers conformational changes in the E1 and E2 proteins that generate the fusogenic conformation of the E1 protein (Gibbons et al., 2004, Haag et al., 2002). Viral-endosomal membrane fusion is followed by the release of the nucleocapsid (NC) core into the host cytosol for initiation of viral replication (Haag et al., 2002).

Structural investigations of alphaviruses have concentrated mainly on arthritogenic alphaviruses (Kostyuchenko et al., 2011, Mukhopadhyay et al., 2006, Smith et al., 1995, Sun et al., 2013, Tang et al., 2011, Zhang et al., 2002, Zhang et al., 2005). In contrast, structural information on encephalitic alphaviruses is limited (Porta et al., 2014, Sherman and Weaver, 2010, Zhang et al., 2011). Encephalitic alphaviruses are considered potential biological weapons, as virus particles can be dispersed as aerosols to initiate infections (Roy et al., 2009). Severe neurological disease is associated with infections of eastern equine encephalitis virus (EEEV), which causes up to 70% fatality rates in symptomatic cases (Armstrong and Andreadis, 2013, Villari et al., 1995). Outbreaks of EEEV have been reported in recent years in the eastern parts of the United States and in Panama (Carrera et al., 2013, Silverman et al., 2013). To gain insight into the molecular organization of encephalitic alphaviruses, we determined a cryo-EM structure of an EEEV virion derived from a SINV-EEEV chimeric virus to a resolution varying from 3.5 to 6.5 Å, corresponding to an average resolution of 4.4 Å. This structure provides information about EEEV entry into host cells (steps 1–4 in Figure S1). Structures of previously reported sequences of a genome binding site (Owen and Kuhn, 1996) and a ribosome binding site (RBS) (Wengler et al., 1992) were observed on the capsid protein. The EEEV cryo-EM map also revealed a binding site for heparan sulfate (HS), which has been linked to viral neurovirulence and avoidance of lymphotropism (Gardner et al., 2011, Gardner et al., 2013). The cryo-EM analysis of EEEV, quantitative glycan analysis, and virus internalization assays provide mechanistic insights into an evasion mechanism by which EEEV inefficiently enters into myeloid lineage cells including macrophages and dendritic cells. Cryo-EM structures of EEEV complexed with Fab fragments from five potently neutralizing monoclonal antibodies (mAbs) provided insights into host cell entry and neutralization.

## Results

**Figure 1:**
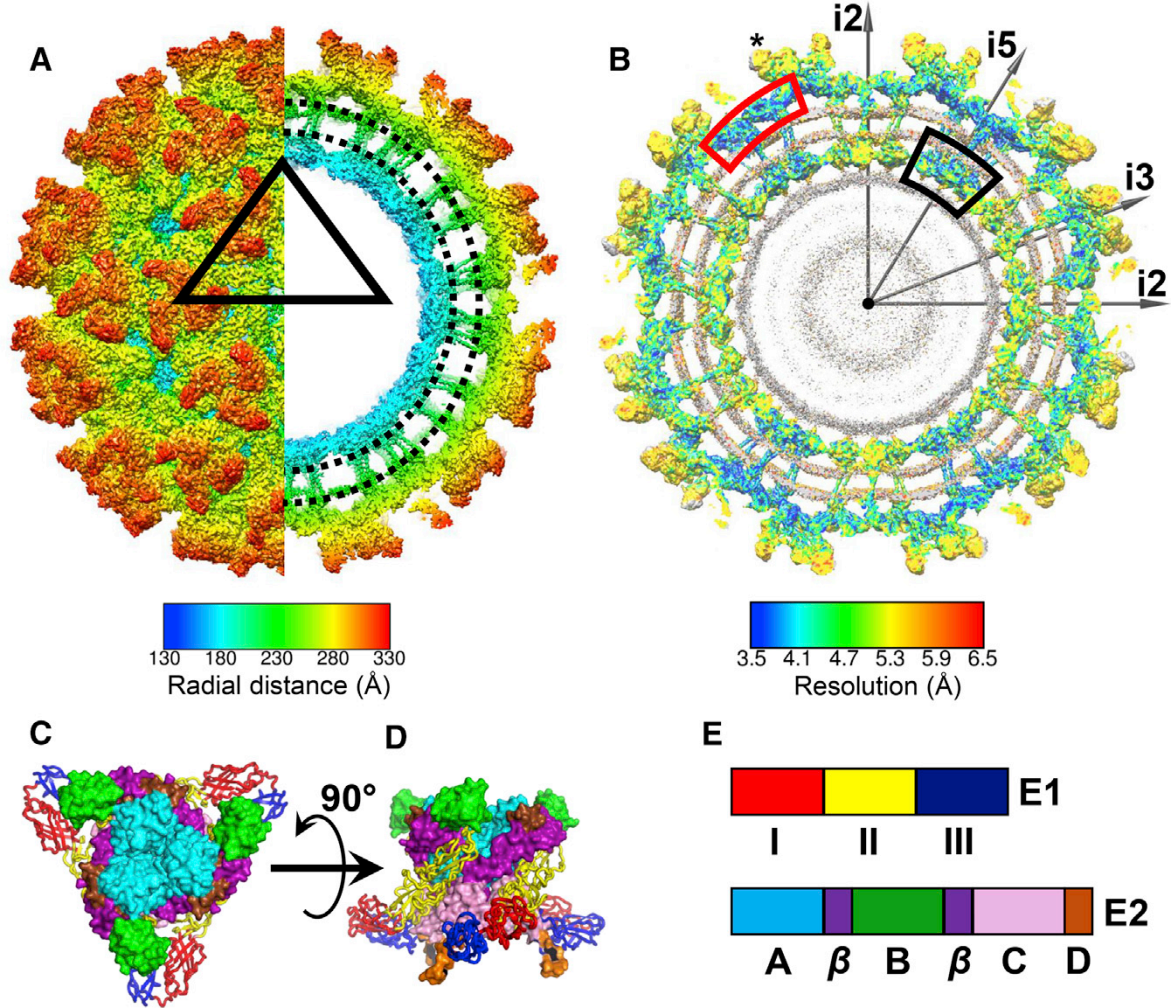
Cryo-EM Structure of EEEV (A) Radially colored surface representation of EEEV. The right half shows a section of the map (lipid bilayer between the black dotted lines). (B) Resolution distribution in the cryo-EM map according to the scale provided at the bottom. The RNA genome (gray) is excluded from the resolution scale. Gray arrows: directions of icosahedral symmetry axes. Red box, E1 ectodomain; black box, capsid proteins near icosahedral 5-fold axis; asterisk, E2 ectodomain. (C–E) Trimeric E1-E2 spikes shown in (C) a radial orientation and (D) rotated by 90°. E1 and E2 are shown using ribbon and surface representation, respectively. (E) Domain distribution in E1 and E2 protein ecto-domains. The color codes follow domain distribution as indicated in (E). See also Figures S1–S3.

The EEEV cryo-EM map shows overall conservation of structural features among alphaviruses (Figures 1A, 1B, S2, and S3A–S3D; Tables S1 and S2). The E1 ectodomain is divided into domains I, II (responsible for fusion), and III. The E2 ectodomain consists of domains A (putative receptor-binding), B (putative receptor-binding and protection of fusion loop), C, D, and a β-ribbon connector (Li et al., 2010, Voss et al., 2010) (Figures 1C–1E). The EEEV E1 and E2 ectodomains are stabilized by disulfide bonds (Figures S3E and S3F). The E1 and E2 ectodomain are resolved to a resolution of ∼3.5–4.0 and ∼4.0–5.5 Å, respectively (Figure 1B), with the relatively poor resolution of the latter due to potential flexibility. The capsid molecules near icosahedral 5-fold axes have a resolution of ∼3.5 Å. In contrast, capsid molecules positioned near other sites have a relatively poor resolution.

### HS Binding Motif

**Figure 2:**
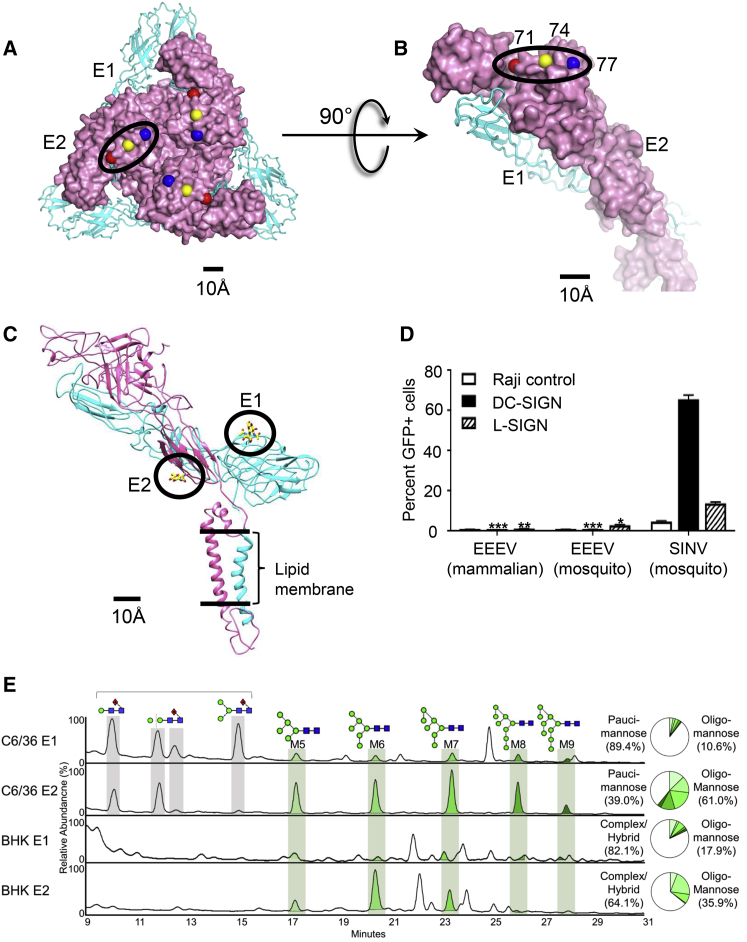
Putative Receptor-Binding Sites on the E1 and E2 Proteins (A) The Cα-atoms of the HS binding Lys residues are shown as spheres (Lys71, red; Lys74, yellow; Lys77, blue) on the E2 ectodomain (pink). One of the three symmetry-related triads in a trimeric spike is marked in a black oval. (B) HS binding residues in one E1-E2 hetero-dimer. (C) N-linked glycosylation sites on E1 (cyan) and E2 (pink) ectodomains, highlighted in black circles. (D) Assay of EEEV infection in Raji cells expressing DC-SIGN and L-SIGN. Infection of L-SIGN expressing Raji cells by EEEV derived from mosquito cells was significantly greater (two-way ANOVA with Tukey’s multiple-comparison test; ^∗^p < 0.0001) than infection of the Raji control cells, whereas infection with mammalian cell-derived virus was not (^∗∗^p > 0.05). Mosquito cell and mammalian cell-derived EEEV infection rates were not significantly higher for DC-SIGN-expressing cells than control cells (^∗∗∗^p > 0.05). SINV is used as a positive control for host cell entry. Three replicates were used for each virus and cell type, and two independent experiments were performed. The error bars represent SD. (E) Quantitative and compositional analysis of EEEV glycosylation. HILIC-UPLC profiles of fluorescently labeled N-linked glycans from EEEV-derived E1 and E2 glycoproteins from C6/36 and BHK-15 cells. Oligomannose-type glycans (M5-M9; Man_5_GlcNAc_2_-Man_9_GlcNAc_2_) (green) were identified by Endo H digestion with quantification of major glycan types summarized in the pie charts. See also Figures S4–S6.

The HS binding phenotype of EEEV is observed in wild-type strains and is not an artifact of cell culture adaptation (Gardner et al., 2011, Gardner et al., 2013). EEEV neurovirulence, HS binding phenotype, and avoidance of lymphotropism are linked to three basic residues, Lys71, Lys74, and Lys77 (“Lys-triad”), on the E2 ectodomain (Gardner et al., 2011, Gardner et al., 2013). Here, these three E2 Lys residues are part of an exposed β-strand and loop on the surface of domain A (Figures 2A and 2B). Lys77 is located closest to the trimeric spike 3-fold axis. The Cα-atoms of adjacent Lys residues are separated by ∼10 Å and form a linear binding site for HS. The symmetry-related Cα-atoms of the three Lys77 residues on a trimeric spike are separated by a distance of ∼25 Å. Among other alphaviruses, none of which interact with HS with the efficiency of EEEV (Gardner et al., 2011), Lys74 is most conserved, Lys77 is least conserved, and Lys71 is often replaced by His (Figure S4). The residue at E2 position 75 is acidic in all alphaviruses except EEEV (Figure S4).

### Envelope Protein Glycosylation

E1-E2 N-linked glycans are potential binding sites for cell surface lectins such as DC-SIGN (dendritic cell-specific intercellular adhesion molecule-3-grabbing non-integrin) and L-SIGN (liver/lymph node-specific intercellular adhesion molecule-3 grabbing non-integrin) (Klimstra et al., 2003). DC-SIGN is expressed in lymphoid dendritic cells and macrophages, whereas L-SIGN is expressed in endothelial cells in lymph nodes, liver sinusoids, and the placenta (Lozach et al., 2007). N-linked glycans are attached to EEEV E1 Asn134 and E2 Asn315, which are each part of the glycosylation motifs Asn134-Ile135-Thr136 and Asn315-Phe316-Thr317, respectively (Figures 2C, S4, and S5). The E1 glycan is accessible on the EEEV surface close to the icosahedral 2-fold and 5-fold vertices (Figures S6A and S6B). However, the E2 glycan is not exposed (Figure 2C). The EEEV cryo-EM map accommodates an *N*-acetyl-glucosamine monosaccharide at the E2 site and a disaccharide at the E1 site (Figure 2C). Of note, at least one glycosylation site on the E2 protein is accessible on the surface of all alphaviruses examined here except for EEEV (Figure S6C).

To evaluate whether EEEV interacts with DC-SIGN or L-SIGN possibly through the exposed E1 glycan, a flow cytometric assay was performed using Raji B lymphoblast cells ectopically expressing DC-SIGN or L-SIGN (Figure 2D). Expression of DC-SIGN and L-SIGN on the surface of Raji cells was previously shown to allow alphavirus binding, entry, and replication in these otherwise receptor-deficient cells (Klimstra et al., 2003). We observed that EEEV was unable to efficiently infect with Raji cells expressing DC-SIGN or L-SIGN as compared to SINV, the positive control (Figure 2D). Thus, unlike other alphaviruses, neurotropic EEEV has limited interactions with DC-SIGN and L-SIGN.

DC-SIGN and L-SIGN have been shown to interact most efficiently with high-mannose carbohydrate modifications, which are a characteristic feature of invertebrate glycosylation pathways including those in arbovirus mosquito vectors (Crispin et al., 2014, Mitchell et al., 2001). To evaluate the carbohydrate composition of the EEEV E1 and E2 glycans, a comparative hydrophilic interaction chromatography ultra-performance liquid chromatography (HILIC-UPLC) analysis was performed using virus cultivated in *Aedes albopictus* C6/36 or mammalian BHK-15 cells (Figure 2E). The exposed E1 glycan consisted of predominantly pauci-mannose carbohydrates in the C6/36 cell-derived virus and complex-type carbohydrates in the BHK-15 cell-derived virus, but not oligo-mannose glycans required for efficient interactions with DC-SIGN and L-SIGN. Therefore, even though EEEV contains an exposed glycan on the E1 ectodomain, the carbohydrate composition of the invertebrate-derived glycan at this site does not favor interactions with these lectins.

### Interactions of EEEV Membrane with Receptors

A third potential alphavirus receptor is the mucin TIM-1, likely because of its ability to bind phosphatidylserine (PS) lipids (Jemielity et al., 2013, Moller-Tank et al., 2013). The EEEV membrane is accessible at the icosahedral 2- and 5-fold vertices (Figures S6D and S6E), which is a conserved structural feature of alphaviruses and presents potential lipid interaction sites with host TIM1. As the diameter of the TIM1 lipid-binding IgV domain is ∼26 Å (PDB ID 5DZO [Yuan et al., 2015]), this receptor could be accommodated near the exposed EEEV membrane at the icosahedral 2-fold vertices; here, the hole exposing the viral membrane has an approximately elliptical shape and a diameter of ∼32 Å along the shorter elliptical axis. In contrast, TIM1-viral membrane interactions at the icosahedral 5-fold vertices (hole diameter, ∼23 Å) would require conformational changes in the E1 ectodomains near the 5-fold axes and possibly in the TIM1 receptor.

### Integrin Binding Sites

Integrins are membrane proteins involved in cellular adhesion (Ruoslahti, 1996). Two integrin-binding motifs are found in the EEEV E2 protein sequence, i.e., an RGD (Arg37-Gly38-Asp39) and a PPG (Pro104-Pro105-Gly106) motif in the E2 ectodomain (Figure S6F), and one motif is found in the E1 ectodomain sequence, i.e., a KGD motif (Lys378-Gly379-Asp380) (Figure S6F). The Pro104-Pro105-Gly106 motif also is found in the E2 protein of encephalitic WEEV and the arthritogenic Mayaro virus (MAYV), RRV, and SINV (Figure S4). All three motifs have been implicated in interactions of viruses with integrins (Chen et al., 2012, La Linn et al., 2005, Mason et al., 1994). The PPG motif also was suggested to be involved in alphavirus interactions with an integrin (La Linn et al., 2005). Of the three integrin binding sites described here, the PPG site is most accessible on the viral surface whereas the RGD site is least exposed as it is located at the E1-E2 interface. The KGD motif is located close to the E1-E1 interface near the icosahedral 2- and 5-fold vertices. However, direct binding interactions of alphaviruses to integrins have yet to be demonstrated.

### Structure of the Capsid Protein

**Figure 3:**
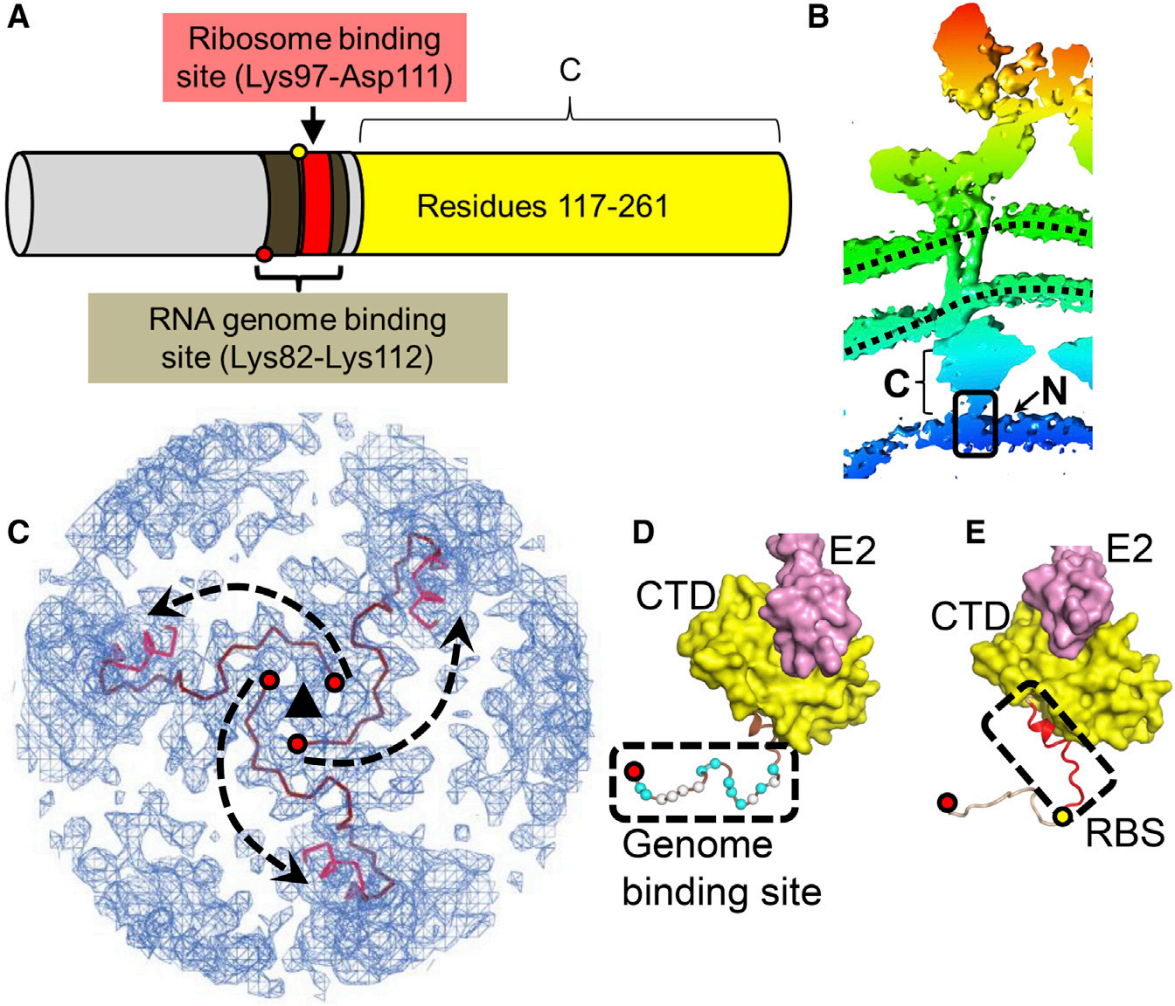
Structure of the EEEV Capsid Protein (A) NTD and CTD are shown in gray and yellow, respectively. (B) Map around one E1-E2-capsid trimer (radial coloring according to scale in Figure 1A; dotted lines: viral membrane). Capsid NTD and CTD are labeled “N” and “C,” respectively. Black box, depth of region shown in (C). (C) Genome binding residues Lys82-Lys112 are shown as extended brown chains. The Lys82 Cα-atom is shown in (C)–(E) to mark the N terminus of the main-chain trace of the capsid genome binding site. (D) Capsid genome binding site (within a black box) and CTD (yellow surface). Red circle, Lys82 Cα-atom; cyan and white circles, Cα-atoms of basic and Pro residues, respectively; pink, E2 endodomain. (E) RBS is shown as a red ribbon enclosed in a box. Yellow circle, Lys97 Cα-atom (see A for reference). See also Figure S7.

The EEEV capsid protein consists of two domains: NTD, residues 1–116, and CTD, residues 117–261 (Figures 3A and 3B). The structure of the alphavirus capsid CTD, which has a chymotrypsin-like fold, has been determined by crystallography (Choi et al., 1991) and cryo-EM (Zhang et al., 2011). The capsid residues Lys81-Arg114 in SINV (Owen and Kuhn, 1996) (corresponding to EEEV capsid Lys82-Lys112) have been implicated in interactions with the RNA genome (Figure 3A). Despite the identification of the NTD genome-binding sequence on the capsid protein more than 20 years ago (Owen and Kuhn, 1996), the structure of this domain has remained elusive, probably because the capsid NTD sequence (Met1-Ile116 in EEEV) shows features characteristic of intrinsically disordered proteins (Uversky, 2013) with high concentrations of basic residues Arg and Lys (27% of the sequence) and structure-disrupting Pro and Gly residues (26% of the sequence).

In the EEEV cryo-EM map, the main-chain coordinates of the genome binding capsid protein residues Lys82-Lys112 were observed to be adjacent to the capsid CTD (Figure 3C). The residues Lys82-Gly99 form an extended network underneath the capsid CTD (Figures 3C and 3D). These residues form an extended coil that contains a high concentration of basic residues and structure-disrupting Pro residues (Figure 3D).

An analysis of the EEEV cryo-EM map at lower contour levels shows multiple conformations of the capsid N-terminal chain, although noise in the map does not allow the main-chain atoms of these other conformations to be traced. In a previous low-resolution reconstruction of SINV, the section of the map corresponding to the Lys82-Gly99 region described here (Figures S7A and S7B) was interpreted to represent genomic RNA and not the capsid NTD (Zhang et al., 2002). The current map of EEEV confirms that the density into which Lys82-Gly99 has been traced is indeed a part of the capsid protein.

**Figure 4:**
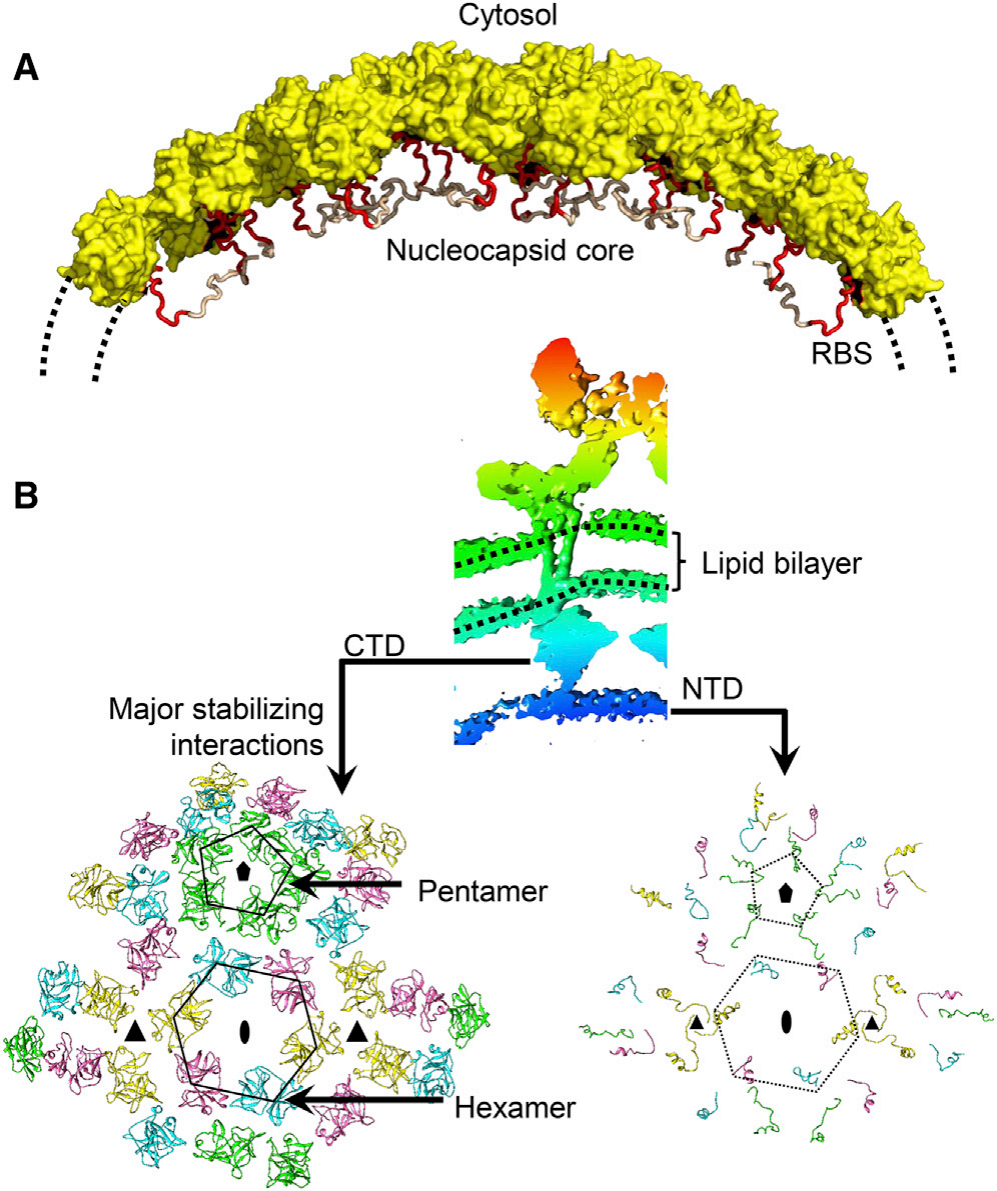
Organization of the Nucleocapsid Core (A) Core surface formed by capsid CTD (yellow). RBSs are colored red. (B) Organization of capsid double-layered shell. Top: cryo-EM map around one E1-E2-capsid trimer (radial coloring according to the scale in Figure 1A; dotted black lines, viral membrane). Bottom panels, left: Pentamers and hexamers of capsid CTD near the icosahedral 5- and 2-fold axes, respectively. No capsid-capsid contacts are observed near the icosahedral 3-fold axes. Right: Capsid genome binding segment. Capsid-capsid contacts are only near the icosahedral 3-fold. See also Figure S7.

The alphavirus capsid protein binds to ribosomes during NC disassembly in the cytosol (Wengler et al., 1992). In EEEV, the residues constituting the RBS (Lys97-Asp111) form a coil and a short helix (Figure 3E). The RBS was located on the inside of the intact NC cores (Figure 4A). This implies that exposure of the RBS requires at least a partial dissociation of the icosahedral capsid shell. The icosahedral capsid shell in EEEV consists of an outer layer formed by the chymotrypsin-like CTD and an inner layer formed by the extended NTD (Figure 4B). The capsid-capsid contacts in the outer capsid layer occur around the icosahedral 2-fold and 5-fold vertices and not around the 3-fold vertices (Figure 4B). Two pairs of residues involved in electrostatic interactions were observed at the capsid-capsid interface in the outer CTD layer: Lys171-Glu233 and Asp173-Arg236. The outer NC layer is stabilized by interactions with the E2 protein endodomains (Figure S7C).

### Electrostatic Interactions at the E1-E2 Dimer

**Figure 5:**
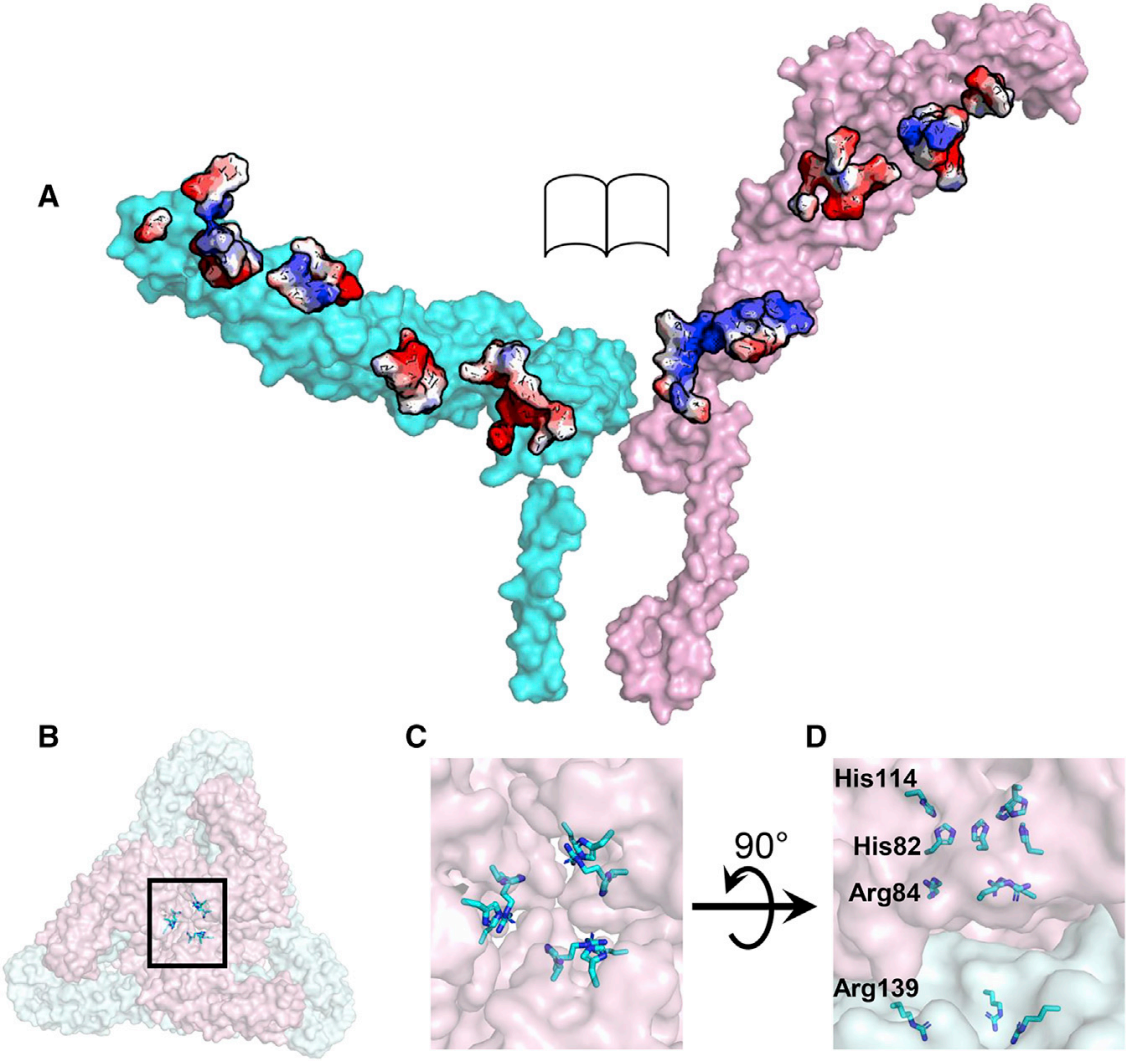
Charged Residues at the E1-E2 Interface (A) Open book view of E1 (cyan) and E2 (pink) proteins showing complementary charged residues at the E1-E2 dimer interface. Acidic and basic residues are colored red and blue, respectively. (B–D) Basic residues in the E2-E2 interface formed by three symmetry-related E2 ectodomains (pink), along the spike 3-fold axis. (B) Trimeric spike along 3-fold axis. The black box encloses the basic residues in the E2-E2 interface. (C) Magnified view of the black box from (B). (D) The four basic residues along the E2-E2 interface from three symmetry-related E2 molecules. See also Figures S4 and S7.

Endosome acidification during alphavirus entry triggers the dissociation of the E1-E2 dimer (Haag et al., 2002). The E1-E2 dimer interface has a concentration of complementary acidic residues on E1 and basic residues on E2 ectodomains (Figure 5A; Table S3). This pH-responsive complementary charged character of E1 and E2 proteins is conserved among alphaviruses (Table S4).

### Formation of Fusogenic E1 Trimers

In the acidic endosomal environment, E1-E2 dimer is followed by the formation of fusogenic E1 trimers, which requires displacement of the E2 ectodomains away from the 3-fold axis of each trimeric spike (Haag et al., 2002). In EEEV, the E2-E2 interface formed by the three E2 ectodomains of each trimeric spike is enriched in pH-responsive basic residues (Figures 5B–5D), which show limited sequence conservation among alphaviruses (Figure S4).

### Inhibition of EEEV Entry by mAbs

The entry-related steps in the alphavirus replication cycle can be exploited as targets for neutralization of infection by mAbs. Hence, a structural analysis was performed of EEEV complexed with Fab fragments derived from five potently neutralizing entry-inhibiting anti-EEEV mouse mAbs, whose production and neutralization characteristics are described elsewhere (Kim et al., 2018).

**Figure 6:**
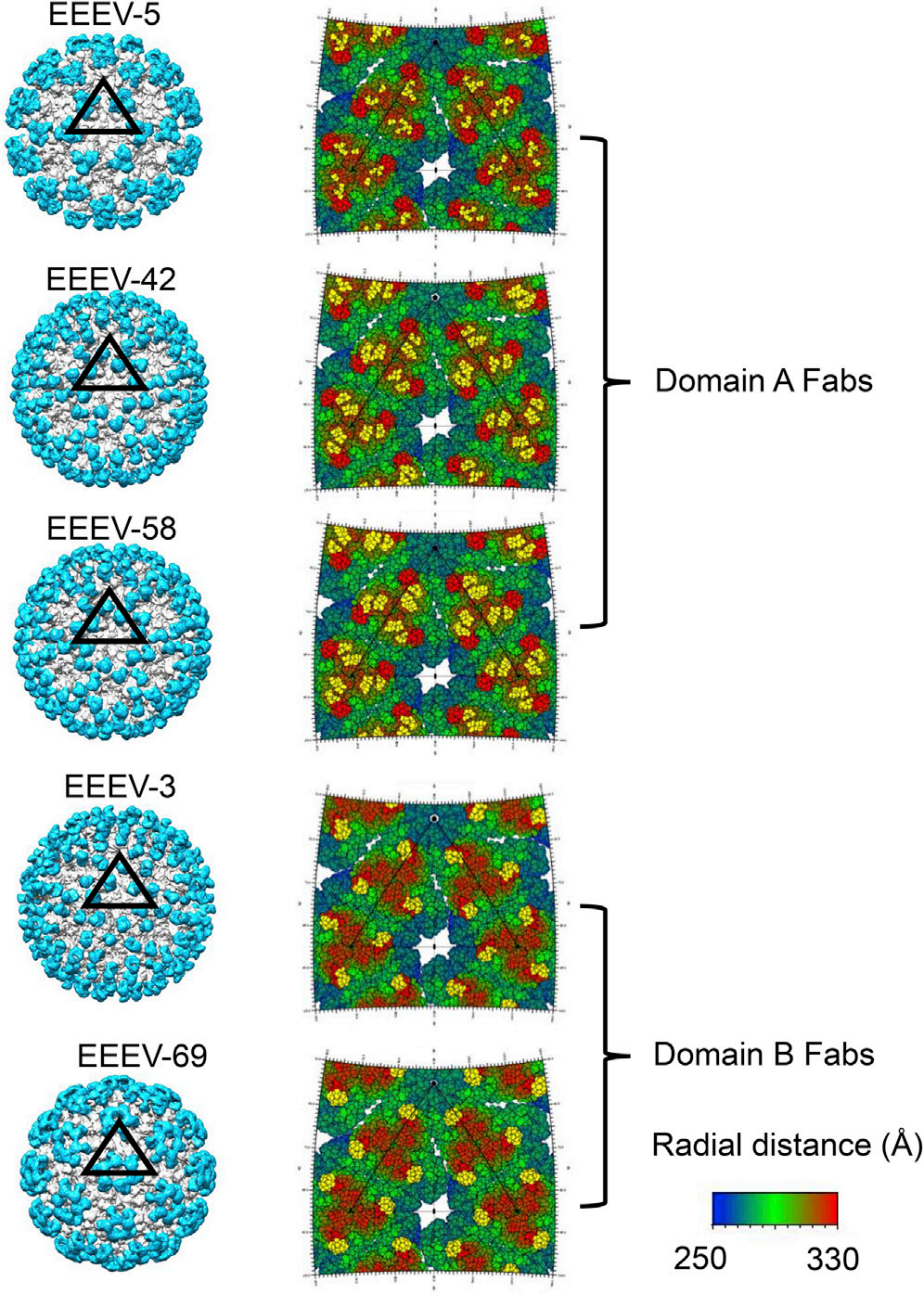
Cryo-EM Maps of EEEV-Fab Complexes The Fabs are bound to E2 domains A and B. Color scheme: Fabs, cyan; E1-E2, white. Right side: Radially colored road-maps of the EEEV surface show contact residues (yellow) constituting the Fab footprint. See also Figures S4 and S7.

The binding footprints of Fab fragments of five neutralizing mAbs (EEEV-3, EEEV-5, EEEV-42, EEEV-58, and EEEV-69) were mapped using cryo-EM structures (Figure 6). EEEV-5, EEEV-42, and EEEV-58 Fabs bound to domain A of E2 utilizing primarily polar interactions (Figure S7D). EEEV-5 and EEEV-42 Fabs interacted with Lys71 and Lys74, two of the three HS binding residues (Gardner et al., 2011), whereas EEEV-58 interacted only with Lys74 of the three HS binding residues (Figure S7D). These three Fabs also make a few interactions with residues in domain B and the β-connector (Figure S7D). In comparison, Fab fragments of EEEV-3 and EEEV-69 interacted exclusively with residues in domain B (Figure S7D). Domains A and B were previously implicated in host cell attachment and pH-triggered conformational changes (Li et al., 2010, Voss et al., 2010). The E2 residues that comprise the footprints of these anti-EEEV mAbs had only limited sequence conservation with other alphaviruses (Figure S7D). Consistent with this observation, the neutralizing activity of these mAbs does not extend to VEEV and WEEV, the related encephalitic alphaviruses (Kim et al., 2018).

**Figure 7:**
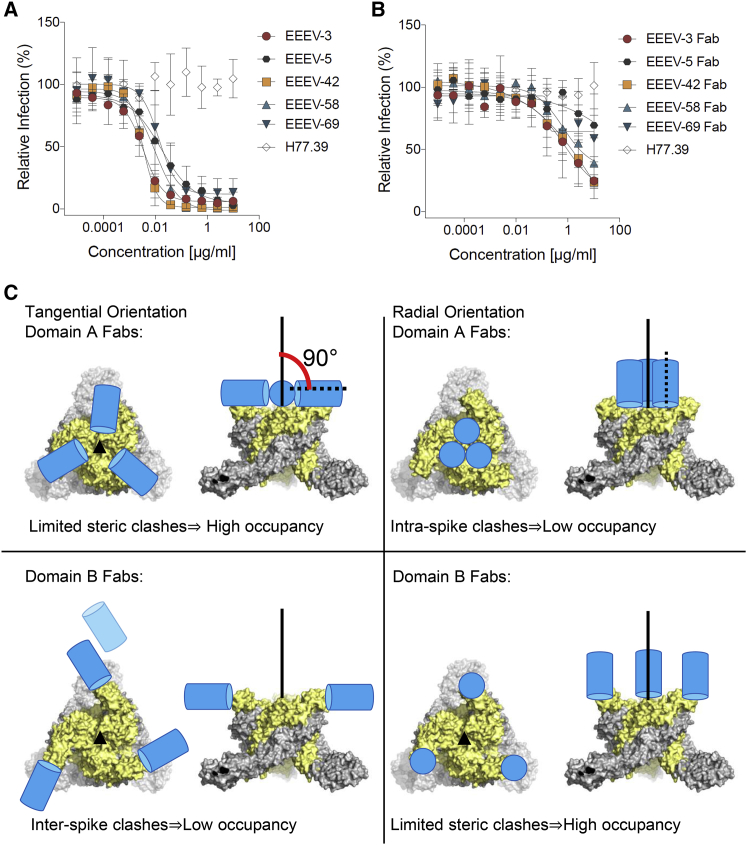
Neutralization of EEEV Infection (A and B) Focus reduction neutralization assay. (A) Bivalent mouse IgG mAbs inhibit infection efficiently unlike (B) monovalent Fabs (hepatitis C virus-specific mAb H77.39, negative control). The neutralization experiments were performed twice, each time in duplicate, and the curves report mean values and SDs. (C) Steric restrictions on Fab binding to alphaviruses. E1-E2 spike, gray-yellow; Fab, blue cylinder; Fab quasi-2-fold axis, broken line. Tangential binding does not cause significant clashes between domain A Fabs bound to a spike (upper left) unlike radial binding (upper right). In contrast, tangential binding of domain B Fabs may encounter clashes with Fabs bound to neighboring spikes and even with neighboring spikes (lower left), unlike radial binding (lower right).

Monovalent Fabs can achieve neutralization of alphavirus infections by either interfering with receptor binding to domain A or by clamping domain B in its neutral-pH conformation, which inhibits subsequent pH-triggered conformational changes. The stabilization of domains A and B can be assessed by calculating the average density of the fitted domain atoms (“sumf” [Rossmann et al., 2001]). In the EEEV-Fab cryo-EM maps (Table S5), the sumf values showed no significant stabilization of either domain A or B upon Fab binding. Indeed, the Fab fragments of the five anti-EEEV mAbs did not inhibit EEEV infection efficiently as confirmed by neutralization assays, which suggests that a bivalent, cross-linking activity may be required to achieve optimal inhibition (Figures 7A and 7B) (Edeling et al., 2014).

Fab occupancies were determined by comparing sumf values (see above for definition) of fitted Fab coordinates with the sumf values of the fitted E1-E2 ectodomains assuming that the ectodomains are present in the virus at 100% occupancy (Table S6). The EEEV-5 Fab had the lowest average occupancy of 45%, whereas the EEEV-58 Fab had the highest average occupancy of 97% (Table S6). The EEEV-Fab complex cryo-EM structures also show diversity in Fab orientations (Figure 6; Table S6). EEEV-5 is the most radial in orientation of the five Fab fragments and forms an angle of 14.5° with the spike 3-fold axis, whereas B domain-specific EEEV-69 is the most tangential with an orientation angle of 52.0° with the spike 3-fold axis.

## Discussion

The overall cryo-EM structure of EEEV described in this investigation is analogous to a previously published VEEV cryo-EM structure (Zhang et al., 2011). However, the present investigation identified features related to EEEV entry and disassembly including the structure of the capsid genome binding segment for which no structural information was previously available.

### Receptor Binding Motifs: Implications for Host Cell Binding

Site-directed mutagenesis of EEEV Lys-triad residues to neutral Ala reduced HS dependence of EEEV infection by ∼90%, indicating that the three E2 Lys residues, especially positions 71 and 74, have major roles in HS binding (Gardner et al., 2011, Gardner et al., 2013). Our study showed that these three EEEV E2 Lys residues form a β-strand close to the 3-fold axis of each trimeric spike. Three symmetry-related copies of this HS binding Lys-triad are exposed on the surface of each trimeric spike. As HS is an extended polymer (Fuster and Wang, 2010), it is possible that the three symmetry-related Lys-triad sites on a trimeric spike are simultaneously engaged in high-avidity HS binding.

Previous investigations have correlated the lack of myeloid cell infectivity of EEEV to a micro-RNA-based translation-inhibition mechanism that suppresses replication of internalized EEEV genomes (Trobaugh et al., 2014). This mechanism circumvents immune system activation that is otherwise a common feature of other alphavirus infections (Trobaugh et al., 2014). We show here that lack of exposure of the E2 high-mannose glycan on the viral surface results in limited lectin-dependent infection of EEEV in cells expressing DC-SIGN or L-SIGN, which includes myeloid cells of the immune system. Along with HS binding, which reduces virus access to lymphoid tissues (Gardner et al., 2011), this lack of lectin-dependent cell entry may be utilized by EEEV to further suppress myeloid cell infection and, together with micro-RNA-mediated inhibition, drive the extreme neurovirulence in mammals.

Another putative mechanism by which alphaviruses interact with host cell membranes is through the viral lipid bilayer, which can bind with TIM1 membrane protein that binds PS lipid (Jemielity et al., 2013). PS is exposed on the outer plasma membrane leaflet upon the induction of apoptosis (Leventis and Grinstein, 2010). Alphavirus infections cause apoptosis of host cells (Levine et al., 1993). Progeny alphavirus particles that bud from the mammalian cell plasma membrane could expose PS on the exposed outer leaflet of the viral membrane. Indeed, PS has been reported in alphavirus membranes (Hirschberg and Robbins, 1974, Laine et al., 1972). Here, it was inferred that TIM1-alphavirus interactions would be favored at the icosahedral 2-fold vertices compared to the 5-fold vertices as the diameter of the holes in the alphaviral envelope is large enough at the 2-fold vertices to accommodate the lipid-binding domain of TIM1. Whether interactions of alphaviruses with TIM1 involve only the lipid membrane or also the envelope proteins is currently not known.

### Electrostatic Interactions at the E1-E2 Dimer Interface: Relevance to Alphavirus Disassembly

Endosome acidification triggers E1-E2 dimer dissociation in internalized alphavirus particles, resulting in viral-endosomal membrane fusion (Wahlberg et al., 1992). The EEEV E1-E2 dimer interface is formed by the acidic E1 and basic E2 proteins. Acidic pH in the endosome can neutralize the negatively charged E1 residues, thereby decreasing dimer stability. As the charged character of E1-E2 proteins is conserved among alphaviruses, this pH-dependent dimer dissociation mechanism is likely to be common to the life cycle of alphaviruses.

Low-pH-induced E1-E2 dimer dissociation is accompanied by trimeric spike disruption. As reported here, the E2-E2 interface along the 3-fold axis of each trimeric spike is enriched in basic residues. Acidic pH would protonate these basic residues, leading to electrostatic repulsion and trimer dissociation. The limited sequence conservation of these basic residues might explain variations in pH requirements for fusion of different alphaviruses in either early or late endosomes (van Duijl-Richter et al., 2015). An estimation of the quantitative contribution of each charged amino acid to the pH-triggered structural changes would require a comprehensive residue-by-residue mutagenesis analysis.

Low pH in the endosome lumen triggers the exposure of the E1 fusion loop peptide. The purified E1 ectodomain is stable as a monomer under neutral pH conditions without the need for stabilizing detergent or lipid (Klimjack et al., 1994, Wahlberg et al., 1992). The crystal structural of the E1 ectodomain showed an exposed fusion loop in the absence of stabilizing detergents or lipids (Lescar et al., 2001). This implies that the exposure of the fusion loop peptide is not sufficient for membrane fusion. Low pH has been implicated in the generation of a fusogenic state of the E1 ectodomain independent of its association with the E2 ectodomain (Klimjack et al., 1994). This E1 fusogenic state then results in the insertion of the E1 fusion loop peptide and additional portions of the E1 ectodomain into the host membrane (Gibbons et al., 2003). As reported here, the alphavirus E1 ectodomain has a conserved acidic character. Neutralization of acidic residues has been reported to serve as a mechanism of enhancement of the hydrophobicity of acidic proteins, which promotes interaction with membranes (Barrera et al., 2011). The conserved acidic character of the E1 ectodomain may provide a mechanism for the insertion of portions of the E1 ectodomain, including the fusion loop, into lipid membranes. In fact, residues outside the E1 fusion loop have been implicated in interactions with membranes indicating that other portions of the E1 ectodomain are also involved in membrane interactions (Chatterjee et al., 2002, Vashishtha et al., 1998).

The assembly and fusion of alphaviruses and flaviviruses in low-pH environments show similarities. The E3 protein prevents alphavirus E1-E2 dimer dissociation during assembly in the low-pH trans-Golgi network (Voss et al., 2010, Zhang et al., 2011). Analogously, the flavivirus prM protein stabilizes flavivirus envelope (E) proteins during assembly and maturation in the acidic Golgi environment (Guirakhoo et al., 1992). Functional similarities are also inferred from homologous structures of alphavirus E1 fusion protein and the flavivirus E fusion protein (Figures S7E and S7F) (Haag et al., 2002, Harrison, 2008, Lescar et al., 2001, Uchime et al., 2013, Wahlberg et al., 1992), with both proteins having an acidic character (Tables S4 and S7).

### Structure of the Capsid Protein

The two-layered structure of the EEEV capsid core described here suggests a mechanism for capsid shell disassembly in the cytosol. Host membrane-to-E1 fusion will decrease the curvature of the E1-E2 layer. As only a short E2 endodomain interacts with the capsid shell, this may initiate “peeling off” of the E1-E2 layer from the NC. In the absence of stabilizing contacts from the envelope protein layer, disassembly of the NC may be initiated at the 3-fold axes that lack stabilizing interactions. Indeed, NCs have been shown to form holes and undergo expansion upon release from alphaviruses, implying that the released NCs are less compact in monomer-monomer associations (Paredes et al., 2003). Moreover, the EEEV capsid-capsid interface is enriched in complementary charged residues, which would be sensitive to pH changes and supports a disassembly mechanism based on acidification (Wengler and Wengler, 2002). Based on the present structural analysis, it could be speculated that the released alphavirus capsid may function as scaffolds that place the viral RNA genome, which acts similarly to a host cell mRNA molecule, next to a ribosome to initiate viral protein synthesis. However, evidence that demonstrates increased efficiency of translation due to ribosome binding next to the mRNA site is currently not available.

The presence of one RNA genome per alphavirus particle and 240 capsid proteins causes heterogeneity in RNA-capsid interactions. However, the icosahedrally averaged EEEV cryo-EM map shows an ordered main-chain for the capsid genome binding residues Lys82-Lys112, implying the occurrence of repetitive RNA-capsid interactions across 60 icosahedral asymmetric units. The occurrence of short sequence repeats in alphavirus genomes (Alam et al., 2014) suggests the possibility of structurally similar RNA-capsid interactions in each icosahedral asymmetric unit.

Non-genomic single-stranded nucleic acids have been reported to initiate NC assembly (Tellinghuisen et al., 1999). However, in the absence of high-resolution reconstructions of artificially assembled NCs, it is currently not possible to evaluate how strictly specific RNA-capsid interactions are required for the assembly of icosahedral NCs.

### Structural Mechanism of EEEV Inhibition by Neutralizing mAbs

The structural analysis of Fab binding described here has general applications for icosahedral viruses, which demonstrate clustering of epitopes close to symmetry axes. This inhibits Fab or mAb binding to symmetry-related epitopes.

In this analysis, only the mAbs and not the Fab fragments achieve significant neutralization unlike the common observation that both bivalent mAbs and monovalent Fabs achieve neutralization (Edeling et al., 2014, Fibriansah et al., 2015). We speculate that cross-linking of E2 domains by bivalent mAbs augments neutralizing activity. An alternative explanation for the poor neutralization potency of Fabs is the loss of avidity due to mAb digestion into a monovalent Fab, although Fabs were able to bind to the E2 protein.

Fab-complexed structures provide insights into steric factors that influence mAb interactions, although saturation binding of the viral envelope proteins is not required for neutralization (Pierson et al., 2007) and Fab occupancies may not directly correlate with mAb potency. Icosahedral symmetry imposes steric constraints that are different for Fabs binding to domain A compared to domain B. In extreme cases, the Fab quasi-2-fold axis can be parallel (“radial”) or perpendicular (“tangential”) to the 3-fold axis of the trimeric spikes (Figure 7C). A Fab bound to domain A in a radial orientation will clash with Fabs bound to the other two symmetry-related positions on the same trimeric spike lowering average Fab occupancy (Figure 7C). This is consistent with the low occupancy of the approximately radially binding EEEV-5 Fab and with the higher occupancy of the more tangentially binding EEEV-58 Fab. Low-occupancy radial binding of Fab or mAb to domain A might result in incomplete blocking of receptor binding sites. In contrast to domain A Fabs, a tangential binding orientation of a domain B Fab will result in clashes with Fabs bound to neighboring spikes and also with E1-E2 dimers from neighboring spikes, thereby lowering the average Fab occupancy (Figure 7C). This will result in incomplete inhibition of pH-triggered conformational changes of domain B.

Here, it seems likely that mAbs whose Fabs bind to E2 domain A cause intraspike cross-linking due to the presence of three symmetry-related copies of domain A near the 3-fold axis of the trimeric spikes. In contrast, mAbs whose Fabs bind to domain B may favor interspike cross-linking as domain B is located at the tip of the E1-E2 dimer. Differences in the occupancies of Fabs that have similar orientations can be attributed to variations in the paratope sequence and Fab affinity for the epitope. This may be the case for EEEV-42 and EEEV-58 Fabs that have similar orientations but different occupancies.

In summary, this investigation has provided mechanistic insights into EEEV entry into host cells and the inhibition of EEEV infections by neutralizing mAbs.

## STAR★Methods

### Key Resources Table

**Table:** 

REAGENT or RESOURCE	SOURCE	IDENTIFIER
**Antibodies**		

EEEV-3	Diamond Lab	Kim et al., 2018
EEEV-5	Diamond Lab	Kim et al., 2018
EEEV-42	Diamond Lab	Kim et al., 2018
EEEV-58	Diamond Lab	Kim et al., 2018
EEEV-69	Diamond Lab	Kim et al., 2018
H77.39	Diamond Lab	Sabo et al., 2011
Peroxidase AffiniPure Goat Anti-Mouse IgG (H+L)	Jackson ImmunoResearch	115-032-062

**Bacterial and Virus Strains**		

SINV(TR339)-EEEV(FL93-939) chimera	Kim et al., 2018	N/A
SINV-TR339	Klimstra et al., 2003	N/A

**Chemicals, Peptides, and Recombinant Proteins**		

Acetonitrile	Fisher Scientific	Cat# 10489553
Procainamide hydrochloride	Abcam	Cat# ab120955
Dithiothreitol	Sigma-Aldrich	Cat# 43819
Ammonium formate buffer	Waters	Cat# 186007081
Sodium cyanoborohydride	Sigma-Aldrich	Cat# 156159
DMSO	Sigma-Aldrich	Cat# D2438
Acetic acid	Fisher Scientific	Cat# 10384970
PNGase F	New England BioLabs	Cat# P0705S
Endoglycosidase H	New England BioLabs	Cat# P0702S
PNGase A	New England BioLabs	Cat# P0707S

**Critical Commercial Assays**		

Pierce Fab Preparation Kit	Thermo Scientific	Cat# 44985

**Deposited Data**		

CryoEM map of native SINV-EEEV chimera (4.4Å)	This paper	EMD-9280
CryoEM map of native SINV-EEEV chimera showing N-terminal chain (4.8Å)	This paper	EMD-9281
CryoEM map of SINV-EEEV chimera with Fab of EEEV-3	This paper	EMD-9274
CryoEM map of SINV-EEEV chimera with Fab of EEEV-5	This paper	EMD-9275
CryoEM map of SINV-EEEV chimera with Fab of EEEV-42	This paper	EMD-9249
CryoEM map of SINV-EEEV chimera with Fab of EEEV-58	This paper	EMD-9278
CryoEM map of SINV-EEEV chimera with Fab of EEEV-69	This paper	EMD-9279
Coordinates of E1-E2-capsid protein for native SINV-EEEV cryoEM map	This paper	PDB ID 6MX4
Coordinates of capsid protein for native SINV-EEEV cryoEM map	This paper	PDB ID 6MX7
Coordinates of E1-E2 ecto-domains and fitted Fab homolog in SINV-EEEV:EEEV-3 cryoEM map	This paper	PDB ID 6MW9
Coordinates of E1-E2 ecto-domains and fitted Fab homolog in SINV-EEEV:EEEV-5 cryoEM map	This paper	PDB ID 6MWC
Coordinates of E1-E2 ecto-domains and fitted Fab homolog in SINV-EEEV:EEEV-42 cryoEM map	This paper	PDB ID 6MUI
Coordinates of E1-E2 ecto-domains and fitted Fab homolog in SINV-EEEV:EEEV-58 cryoEM map	This paper	PDB ID 6MWV
Coordinates of E1-E2 ecto-domains and fitted Fab homolog in SINV-EEEV:EEEV-69 cryoEM map	This paper	PDB ID 6MWX

**Experimental Models: Cell Lines**		

African green monkey kidney (Vero) cell	WHO Reference Cell Bank	WHO Vero cells
Baby Hamster Kidney (BHK)-15 cells	Kuhn Lab (Purdue University)	N/A
*Aedes albopictus* C6/36	ATCC	Cat#CRL-1660
Raji B cells expressing DC-SIGN	Kwon et al., 2002	N/A
Raji B cells expressing L-SIGN	Kwon et al., 2002	N/A
Raji B cells	Kwon et al., 2002	N/A

**Software and Algorithms**		

Prism	GraphPad Software	Prism Version 7.0d
Empower 3.0	Waters	N/A
NetNGlyc 1.0	http://www.cbs.dtu.dk/services/NetNGlyc/	N/A
Leginon	Carragher et al., 2000	N/A
Motioncor2	Zheng et al., 2017	N/A
CTFFIND4	Rohou and Grigorieff, 2015	N/A
Appion	Lander et al., 2009	N/A
Relion	Scheres, 2012	N/A
jspr	Guo and Jiang, 2014	N/A
ResMap	Kucukelbir et al., 2014	N/A
Phenix Real Space Refine	Adams et al., 2010	N/A
Rosetta	Alford et al., 2017	N/A
MolProbity	Chen et al., 2010	N/A
COOT	Emsley and Cowtan, 2004	N/A
PyMol	https://pymol.org/2/	N/A
Chimera	Pettersen et al., 2004	N/A
RIVEM	Xiao and Rossmann, 2007	N/A
EMfit	Rossmann et al., 2001	N/A
I-TASSER	Yang et al., 2015	N/A
Clustal Omega	Sievers et al., 2011	N/A
ESpript	Gouet et al., 2003	N/A
ExPasy	Gasteiger et al., 2003	N/A

**Other**		

Spe-ed Amide 2 cartridges	Applied Separations	Cat# 4821
Glycan BEH Amide column (2.1 mm x 100 mm, 1.7 μM)	Waters	Cat# 186004741
PVDF protein-binding membrane	Millipore	Cat# MAIPS4510
Ultrathin continuous carbon grids	Ted Pella	Cat# 01824

### Contact for Reagent and Resource Sharing

Further information and requests for resources and reagents should be directed to and will be fulfilled by the Lead Contact Michael G. Rossmann (mr@purdue.edu). An approved Material Transfer Agreement (MTA) may be required for resource sharing.

### Experimental Model and Subject Details

Baby hamster kidney (BHK)-15 cells were obtained from the laboratory of Richard J. Kuhn (Purdue University). Vero cells were obtained from the WHO Reference Cell Bank. C6/36 cells were obtained from ATCC. Raji B cells including those engineered to express DC-SIGN and L-SIGN were obtained from the laboratory of Dan Littman, New York University School of Medicine. The construction of the SINV (TR339 strain)-EEEV (FL93-939 strain) chimera is reported elsewhere (Kim et al., 2018). All virus-infected cells were handled following biosafety level-2 containment safety procedures defined in institutional biosafety protocols.

### Method Details

#### Purification of SINV-EEEV chimeric particles from mammalian cells

EEEV purification was optimized from a previously published alphavirus purification protocol (Zhang et al., 2011). Chimeric particles were grown in BHK-15 cells after infection of ∼80% confluent cells at multiplicity of infection ∼5 under biological safety level (BSL) 2 containment conditions. Modified Eagle Medium (MEM) growth medium supplemented with 2% fetal bovine serum (FBS) and 1X non-essential amino acids (NEAA) was collected after a post-infection incubation of 37°C for 16 h and clarified of cell debris by centrifugation. EEEV particles in the clarified growth medium were then subjected to precipitation with 14% (weight/volume) PEG-6000 and 4.6% (weight/volume) NaCl. The growth medium was then centrifuged at 2,500 x g at 4°C for 30 min to pellet EEEV particles. Further purification of EEEV particles was performed using a linear, continuous 0%–90% Optiprep gradient centrifuged at 247,000 x g for 1 h at 4°C. The purified EEEV particles were concentrated to 1-2 mg/ml of E2 protein for cryoEM.

#### Purification of SINV-EEEV chimeric particles from mosquito cells

*Aedes albopictus* mosquito C6/36 cells were cultured to ∼90% confluence at 28-30°C in Dulbecco’s Modified Eagle Medium (DMEM) supplemented with 10% FBS, 1X non-essential amino acids and 1X streptomycin-pencillin. EEEV infection was performed using virus diluted to an MOI of ∼10 in DMEM followed by gentle rocking at 25°C for 1-2 hours. The EEEV containing medium was then removed, infected cells washed with 1X PBS and incubated for ∼30 hours in the presence of DMEM (1X), FBS (2%) and NEAA (1X). Purification of EEEV from C6/36 cells was performed as described earlier for alphavirus produced in BHK-15 cells (Mukhopadhyay et al., 2006).

#### CryoEM data collection

Purified EEEV particles were flash frozen on lacey carbon EM grids in liquid ethane under BSL-2 containment conditions. For cryoEM analysis, movies of the frozen EEEV particles were recorded with the software Leginon (Carragher et al., 2000) using a Gatan K2 direct electron detector attached to a 300 keV Titan-Krios microscope using a dose of ∼8 electrons per second.

#### CryoEM data processing

CryoEM movies (55 frames, 200 msec exposure per frame) were corrected for beam-induced motion using MotionCor2 (Zheng et al., 2017). Estimation of contrast transfer function (CTF) parameters was performed in CTFFIN4 (Rohou and Grigorieff, 2015). EEEV particles selected from micrographs using the software Appion (Lander et al., 2009) were subjected to reference-free 2D classification in RELION (Scheres, 2012) to identify a subset of homogeneous EEEV particles. A *de-novo* initial model from a small fraction of EEEV particles in *jspr* (Guo and Jiang, 2014) and was used for 2D alignment and 3D reconstruction by projection-matching. Refinement of particle center and orientation angles yielded a resolution of 8.5Å. Refinement of higher order parameters, i.e., defocus, beam tilt, astigmatism and anisotropic magnification improved the resolution of the cryoEM map to a resolution range of 3.5Å to 6.5Å, corresponding to an average resolution of 4.4Å. The resolution of the map was estimated corresponding to a gold-standard Fourier Shell Correlation (FSC) coefficient of 0.143 and the resolution range was calculated using the software ResMap (Kucukelbir et al., 2014, Rosenthal and Henderson, 2003, Scheres and Chen, 2012). The sharpening of the cryoEM map was performed in RELION using a B-factor of −198Å^2^.

#### CryoEM model building

Homology models of EEEV E1 and E2 proteins and a structure of VEEV capsid CTD (Yang et al., 2015, Zhang et al., 2011) modified to EEEV capsid sequence were re-built in Rosetta (Alford et al., 2017) to improve fitting of the coordinates into the EEEV cryoEM map. The resulting coordinates were subjected to an iterative protocol of manual rebuilding in Coot (Emsley and Cowtan, 2004) and refinement against the cryoEM map in phenix.real-space.refine (Adams et al., 2010) to improve coordinate fitting by minimizing Ramachandran outliers (Table S2). Main-chain coordinates of residues Lys82-Gly99 of the capsid protein NTD directly preceding the capsid CTD were traced in the map in Coot and refined against the map in phenix.real-space.refine. The EEEV cryoEM map showed evidence of glycosylation of E1 and E2 ectodomains at Asn134 and Asn315 respectively. Carbohydrates were built in Coot. The quality of the protein models was evaluated using Molprobity (Chen et al., 2010).

#### Interpretation of EEEV-Fab complex structures

Five cryoEM structures of EEEV-Fab complexes were interpreted by fitting coordinates of E1-E2 ectodomains and an alphavirus Fab homolog, PDB ID 5ANY (Long et al., 2015) using EMfit assuming T = 4 quasi-symmetry in the icosahedral asymmetric unit. A quantity “sumf” that measures average electron density around fitted atoms was determined for E1-E2 ectodomains and Fabs fitted in each map assuming T = 4 icosahedral symmetry (Rossmann et al., 2001). The occupancies of fitted Fabs were determined by scaling Fab sumf values to averaged E1-E2 sumf values assuming that E1-E2 are present at 100% occupancy in each map. Roadmaps of Fab footprints on the EEEV E2 protein were generated using the program RIVEM (Xiao and Rossmann, 2007).

The orientation angles for Fabs with respect to the spike 3-fold axis were calculated as follows. Fabs consist of four domains: heavy (V_H_) and light (V_L_) chain variable domains and heavy (C_H_) and light (C_L_) chain constant domains. A quasi-2-fold axis relates the V_H_-C_H_ domains to the V_L_-C_L_ domains. The centers of mass of all four domains were determined individually. Then, an average value was calculated for the center of mass for the V_H_-V_L_ pair and another for the C_H_-C_L_ pair. The Fab quasi-2-fold axis was represented as a vector extending from the average center of mass for the V_H_-V_L_ pair to the C_H_-C_L_ pair. Fab orientations were calculated between the Fab quasi-2-fold vector and the spike 3-fold vector.

#### Calculation of isoelectric points

Isoelectric points were calculated using the Expasy online server (Gasteiger et al., 2003). Sequence alignments were performed using Clustal-Omega (Sievers et al., 2011).The accession numbers for E1 and E2 sequences are: (EEEV), E1, NP_740648.1 and E2, ANB41727.1; (WEEV), E1, ACT75276.1 and E2, ABD57956.1; (VEEV), E1, AAD37000.1 and E2, AAU89534.1; (SINV), E1 NP_740677.1; (CHIKV), E1, AUS84459.1 and E2, ABN04188.1; (MAYV), E1, NP_579970.1 and E2, NP_579970.1, (RRV), E1, P08491 and E2, P08491. The SINV TR339 E2 sequence was obtained from (McKnight et al., 1996). Accession numbers for flavivirus E protein sequences are: Dengue virus serotype 1 (DENV1): GQ398255; Dengue virus serotype 2 (DENV2): NC_001474; Dengue virus serotype 3 (DENV3): EU081190; Dengue virus serotype 4 (DENV4): GQ398256; Japanese encephalitis virus (JEV): D90194; West Nile virus (WNV): DQ211652; Yellow fever virus Asibi (YFV_Asibi): AY640589; Zika virus (ZIKV_HPF): KJ776791.

#### Prediction of glycosylation sites

The sequences of E1 and E2 proteins listed above were analyzed for N-linked glycosylation motifs (Asn-X-Ser/Thr; X, any residue except Pro) (Gavel and von Heijne, 1990) using the NetNGlyc 1.0 software (http://www.cbs.dtu.dk/services/NetNGlyc/).

#### Quantitative glycan analysis by hydrophilic interaction chromatography-ultra performance liquid chromatography (HILIC-UPLC)

The E1 and E2 proteins of C6/36 and BHK-15 derived EEEV samples were separated by denaturing SDS-PAGE. Protein bands were excised for HILIC-UPLC. EEEV samples were inactivated by heating at 75°C for 5 minutes and then at 60°C for 15 minutes in the presence of 1% SDS for quantitative glycan analysis. Excised EEEV E1 and E2 gel bands were washed with alternate washes of acetonitrile and water before drying in a vacuum centrifuge. Bands were rehydrated with 100 μl of water and incubated with PNGase A and F at 37°C overnight. Released N-linked glycans were labeled by overnight incubation at 65°C with procainamide, using a labeling mixture of 110 mg/ml procainamide and 60 mg/ml sodium cyanoborohydrate in 70%DMSO and 30% glacial acetic acid. Labeled glycans were analyzed using a 2.1 mm × 10 mm Acquity BEH Glycan column (Waters) on a Waters Acquity H-Class UPLC instrument as performed in (Pritchard et al., 2015) with wavelengths of λ_ex_ = 310 and λ_em_ = 370. Endo H digestions of labeled glycans were used to quantify the abundance of oligomannose-type glycans, as previously described (Pritchard et al., 2015).

#### Flow cytometry analysis

Parental control Raji cells and Raji cells stably transfected with human DC-SIGN and L-SIGN were generously provided (Dan Littman, New York University School of Medicine) (Kwon et al., 2002). Cells were infected with GFP-expressing EEEV and SINV TR339 viruses exactly as described (Klimstra et al., 2003). At 18 hours post infection, cells were fixed in 4% paraformaldehyde and examined for GFP expression by flow cytometry.

#### mAb production

The generation, isolation and characterization of mouse mAbs has been described elsewhere (Kim et al., 2018). Fabs were generated from the mAbs by digestion with papain using a commercial Fab preparation kit from Thermo Fisher Scientfic following the manufacturer’s instructions.

#### Focus reduction neutralization assay

Vero cells were seeded at 3 × 10^5^ cells/well in a 96-well flat bottom plate 24 h prior to assay. mAbs or Fab fragments were diluted in DMEM supplemented with 5% fetal bovine serum (FBS), 100 U/ml penicillin, 100 μg/ml streptomycin and 10 mM HEPES and incubated with 100 FFU of EEEV for 1 h at 37°C. Virus-mAb or virus-Fab complex was added to the cell monolayer, and after a 1.5 h incubation, cells were overlaid with MEM containing 2% FBS and 1% (w/v) methylcellulose. Infection was allowed to proceed for 18 h and then fixed with 1% paraformaldehyde in phosphate buffered saline for 1 h. Plates were incubated with EEEV-10 (Kim et al., 2018) and subsequently with horseradish peroxidase-conjugated goat anti-human IgG. To visualize EEEV infected cell foci, TrueBlue peroxidase substrate was added to the plates and quantitated using an ImmunoSpot 5.0.37 macroanalyzer (Cellular Technologies Ltd). Neutralization curves were normalized to infected wells containing no mAbs and fitted using a nonlinear regression model. The HCV-specific mAb H77.39 was used as a negative control (Sabo et al., 2011).

Figures were prepared in Chimera (Pettersen et al., 2004), Coot (Emsley and Cowtan, 2004), PyMol and ESpript (http://espript.ibcp.fr/ESPript/ESPript/) (Gouet et al., 2003).

### Quantification and Statistical Analysis

CryoEM reconstructions were performed using a “gold-standard” method (Scheres and Chen, 2012). Briefly, each particle data-set was divided randomly into two halves. Each half-set was reconstructed independently to minimize overfitting. The resolution of each cryoEM map was determined using a Fourier shell correlation (FSC) coefficient of 0.143 between the two independently reconstructed half-maps (Rosenthal and Henderson, 2003). Data in resolution shells whose correlation was lower than 0.143 were omitted from the final maps. The reconstruction software *jspr* (Guo and Jiang, 2014) was used to calculate the FSC curves. Virus infection assays followed by flow cytometry were evaluated using two-way ANOVA with Tukey’s multiple comparison test to identify the means whose values were significantly different from the remaining means. Neutralization experiments were performed twice, each time in duplicate. The neutralization curves report mean-values and standard deviations.

### Data and Software Availability

All software and programs used in this paper for structure determination are available freely and are discussed in detail above. See the KEY RESOURCES table for the relevant references. The cryoEM maps and coordinates have been uploaded to the Electron Microscopy databank (EMDB) and Protein Data Bank (PDB) with the following accession codes: EEEV 4.4Å map (EMD-9280, PDB ID 6MX4), EEEV capsid N-terminal domain (EMD-9281, PDB ID 6MX7), EEEV complexed with Fab of EEEV-3 (EMD-9274, PDB ID 6MW9), EEEV complexed with Fab of EEEV-5 (EMD-9275, PDB ID 6MWC), EEEV complexed with Fab of EEEV-42 (EMD-9249, PDB ID 6MUI), EEEV complexed with Fab of EEEV-58 (EMD-9278, PDB ID 6MWV) and EEEV complexed with Fab of EEEV-69 (EMD-9279, PDB ID 6MWX).

## Supplemental Information

Document S1. Figures S1–S7 and Tables S1–S7

Document S2. Article plus Supplemental Information
