# Supplementary material for: Cryo-EM Structures of Eastern Equine Encephalitis Virus Reveal Mechanisms of Virus Disassembly and Antibody Neutralization
Source: Cell Rep. 2018 Dec 11;25(11):3136–3147.e5. doi: 10.1016/j.celrep.2018.11.067 (PMC6302666; doi:10.1016/j.celrep.2018.11.067)
Supplement: Document S1. Figures S1–S7 and Tables S1–S7 [file mmc1.pdf]

Cell Reports, Volume *25*

Supplemental Information

Cryo-EM Structures of Eastern Equine Encephalitis Virus Reveal Mechanisms of Virus Disassembly and Antibody Neutralization

S. Saif Hasan, Chengqun Sun, Arthur S. Kim, Yasunori Watanabe, Chun-Liang Chen, Thomas Klose, Geeta Buda, Max Crispin, Michael S. Diamond, William B. Klimstra, and Michael G. Rossmann

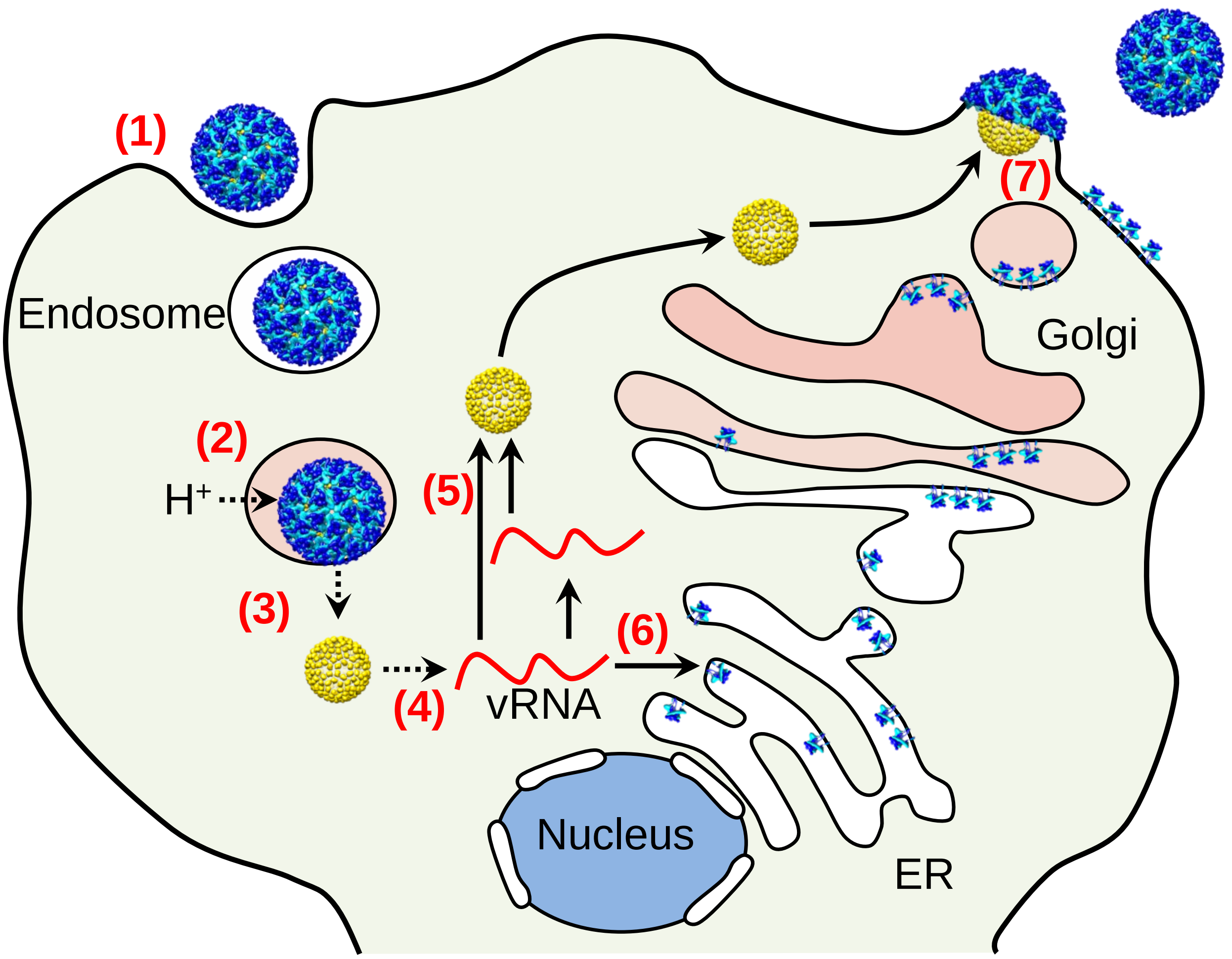

Figure S1. Life cycle of alphaviruses, Related to **Figure 1**. The steps involved in virus entry and disassembly are labelled in red from (**1**) to (**4**). The steps involved in progeny virus assembly and exit are labelled in red from (**5**) to (**7**). (**Step 1**) Alphavirus particles (blue, cyan) enter host cells by receptor-mediated endocytosis. (**Step 2**) The acidification of the endosome lumen (represented in pink) triggers fusion of the viral and endosomal membranes. (**Step 3**) The nucleocapsid core (yellow) is released into the cytosol. (**Step 4**) The core disintegrates to release the viral RNA genome (vRNA, red line). (**Step 5**) The synthesis of progeny RNA genome and nucleocapsid core takes place in the cytosol, whereas envelope protein synthesis involves the ER and Golgi network (**Step 6**). (**Step 7**) The assembly of progeny particles takes place at the plasma membrane.

Vertical axis: Fourier shell correlation (FSC)
Horizontal axis: 1/Resolution ($Å^{-1}$)

Hasan *et al.*, Figure S2

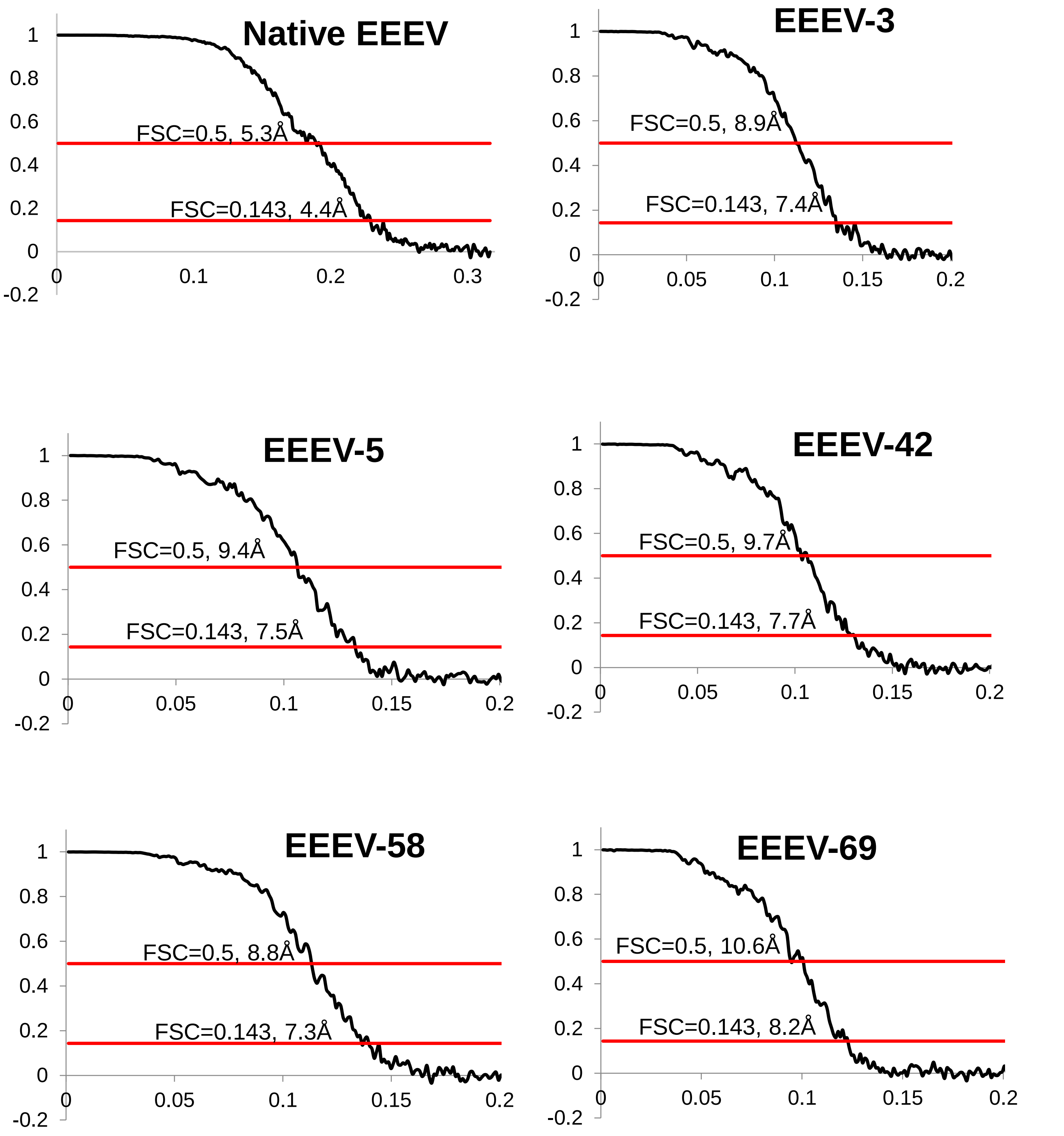

Figure S2. Fourier shell correlation (FSC) curves showing the resolutions of the native EEEV map and resolutions of five EEEV-Fab complex cryoEM maps, Related to **Figure 1**.

Statistics: Particles in each cryoEM reconstruction- native=30,806; EEEV+Fab3=8,416; EEEV+Fab5=6,583; EEEV+Fab42=4,733; EEEV+Fab58=7,335; EEEV+Fab69=5,964.

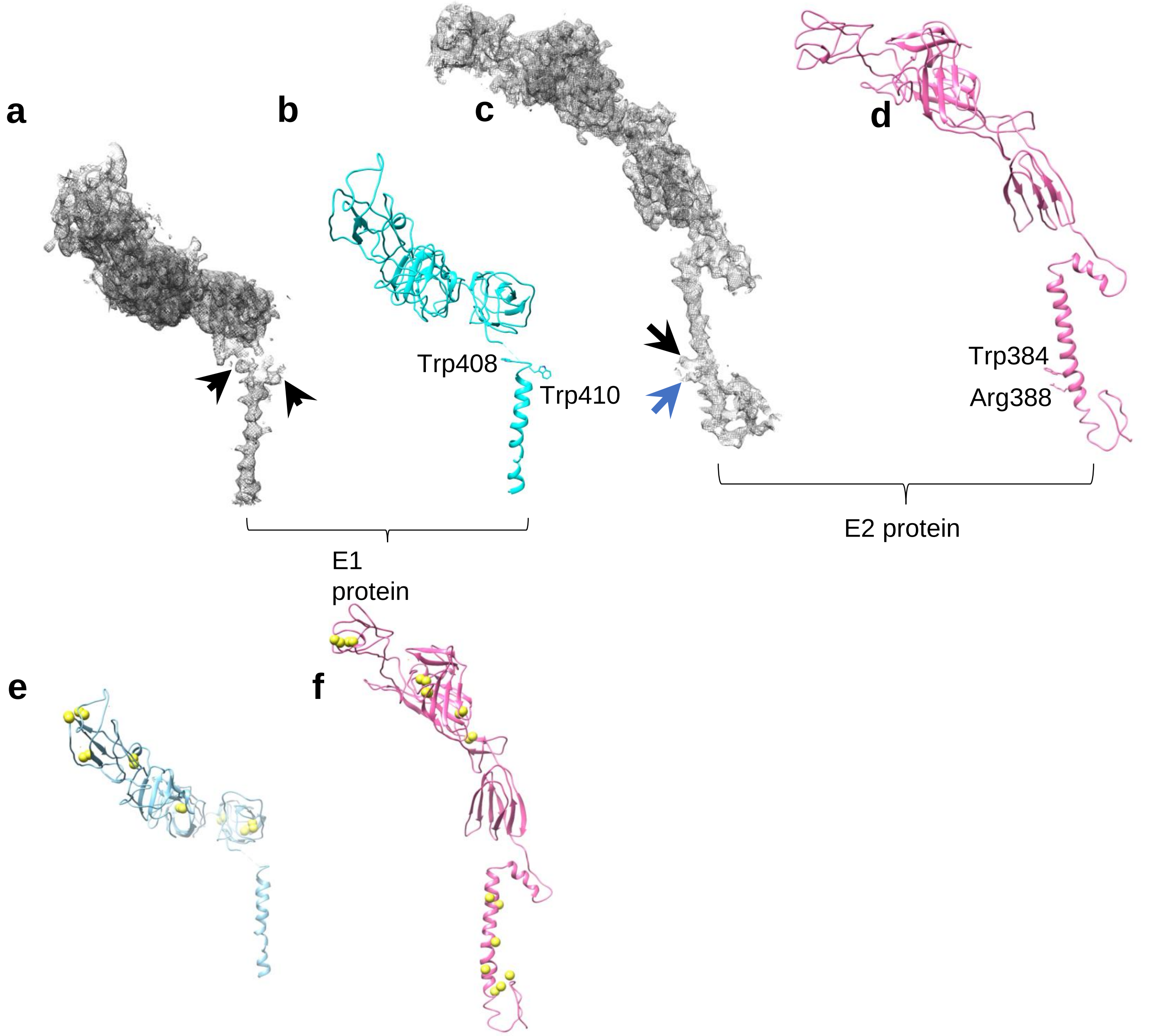

Figure S3. Structure of E1-E2 proteins, Related to **Figure 1**. (**a**) Map and (**b**) coordinates of the E1 protein, and (**c**) map and (**d**) coordinates of the E2 protein are shown. The black arrows point to aromatic residues whereas the blue arrow points to an Arg residue. (**e, f**) Cys residues in EEEV envelope proteins E1 and E2. The side-chain S-atoms of Cys residues are shown as yellow spheres. The Cys residues in E1 are restricted to the ecto-domain.

Hasan *et al*., Figure S4

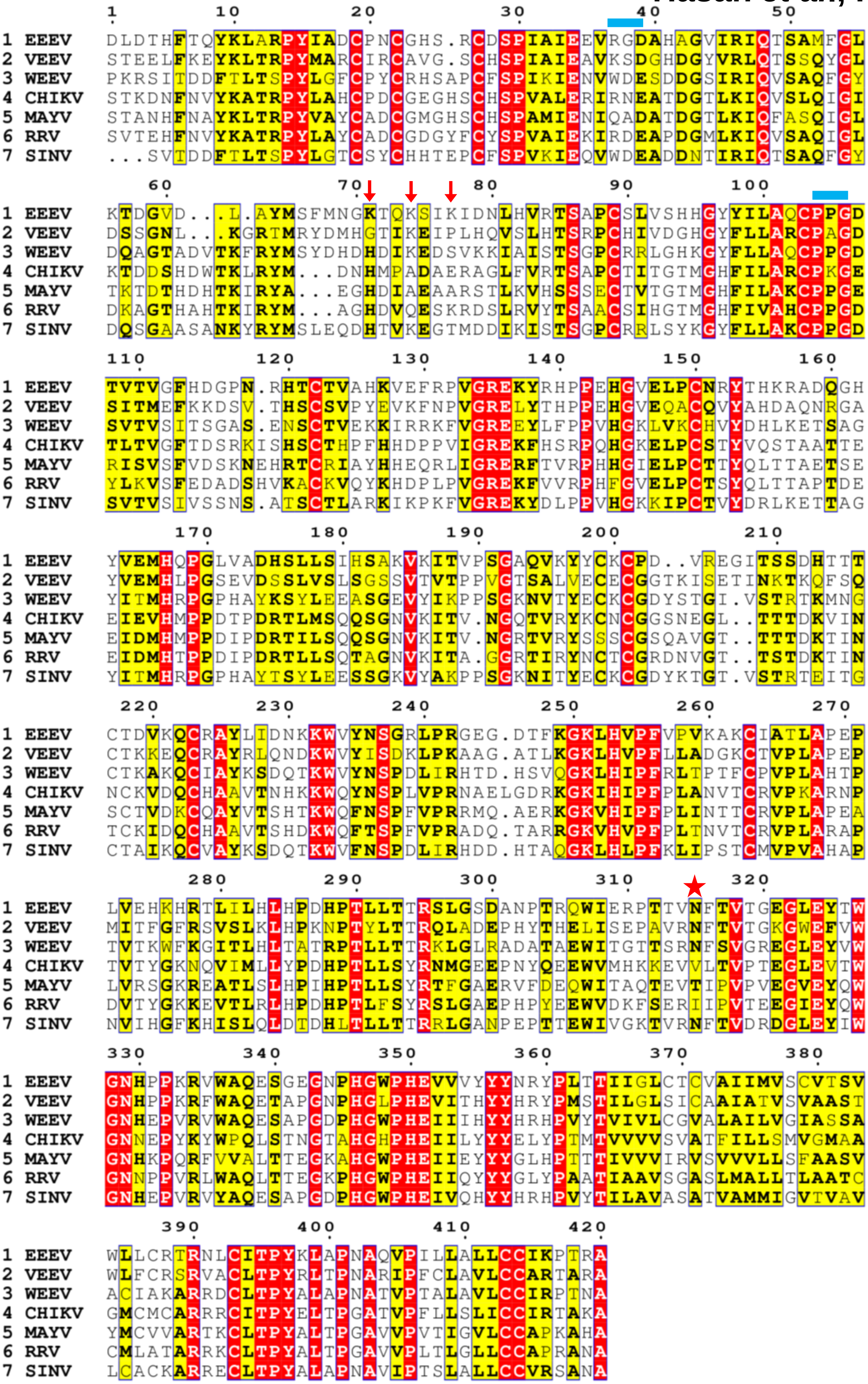

Figure S4. **Multiple sequence alignment of alphavirus E2 protein sequences,** Related to **Figure 2, 5, and 6**. Glycosylation motif is shown in a blue box. Sequence conservation color code: red, complete; yellow, partial; white, none.

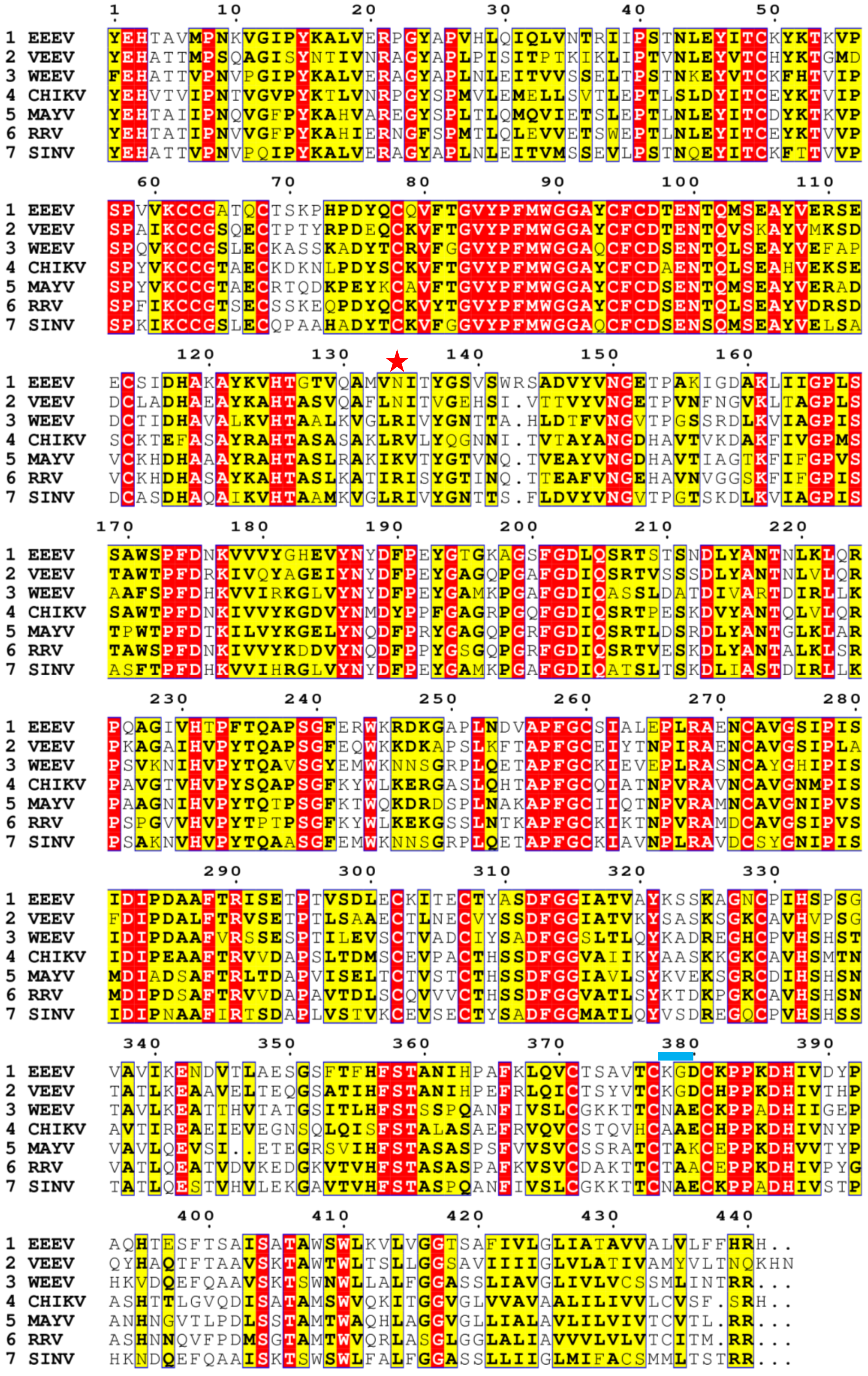

Figure S5. Multiple sequence alignment of alphavirus E1 protein sequences, Related to **Figure 2, and 6**. Glycosylation motif is shown in a blue box. Sequence conservation color code: red, complete; yellow, partial; white, none.

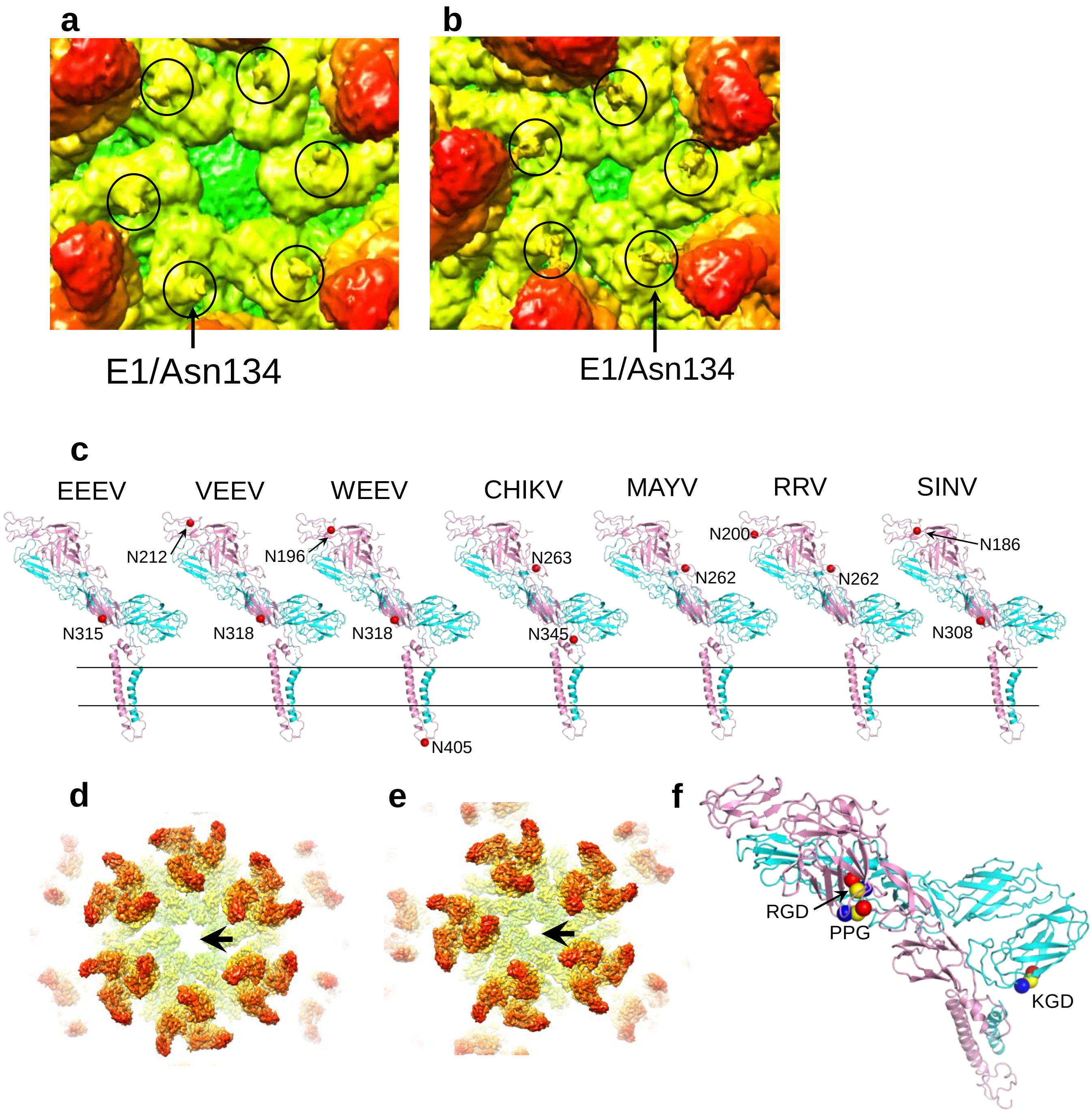

Figure S6. Envelope protein glycosylation and receptor binding sites, Related to **Figure 2**. Symmetry-related carbohydrate sites are shown near the (a) icosahedral 2-fold and (b) 5-fold vertices. The black ovals highlight symmetry-related carbohydrate sites. The map is unsharpened and colored according to the radial scale in Figure 1a. (**c**) Glycosylation sites in alphavirus receptor-binding E2 protein. The Cα-atom of Asn residue in a glycosylation motif is shown as a red sphere modeled in the EEEV E1-E2 dimer structure. E1, cyan; E2, pink. (**d, e, f**) Putative receptor binding sites in EEEV. Exposed viral membrane at the icosahedral (**d**) 2-fold and (e) 5-fold vertices for TIM1 interactions. Black arrows point to the exposed membrane. The map is colored according to the radial scale in Figure 1a. (**f**) Integrin binding motifs in EEEV E1-E2 dimer. Each motif consists of three amino acids as described here and in the main text. The C-atoms of each amino acid in the motif are shown as spheres (red, yellow, blue from N to C terminus). EEEV E2 motifs: RGD (Arg37-Gly38-Asp39) and PPG (Pro104-Pro105-Gly106). E1 motif: KGD motif (Lys378-Gly379-Asp380). E1, cyan; E2, pink.

Hasan *et al.*, Figure S7

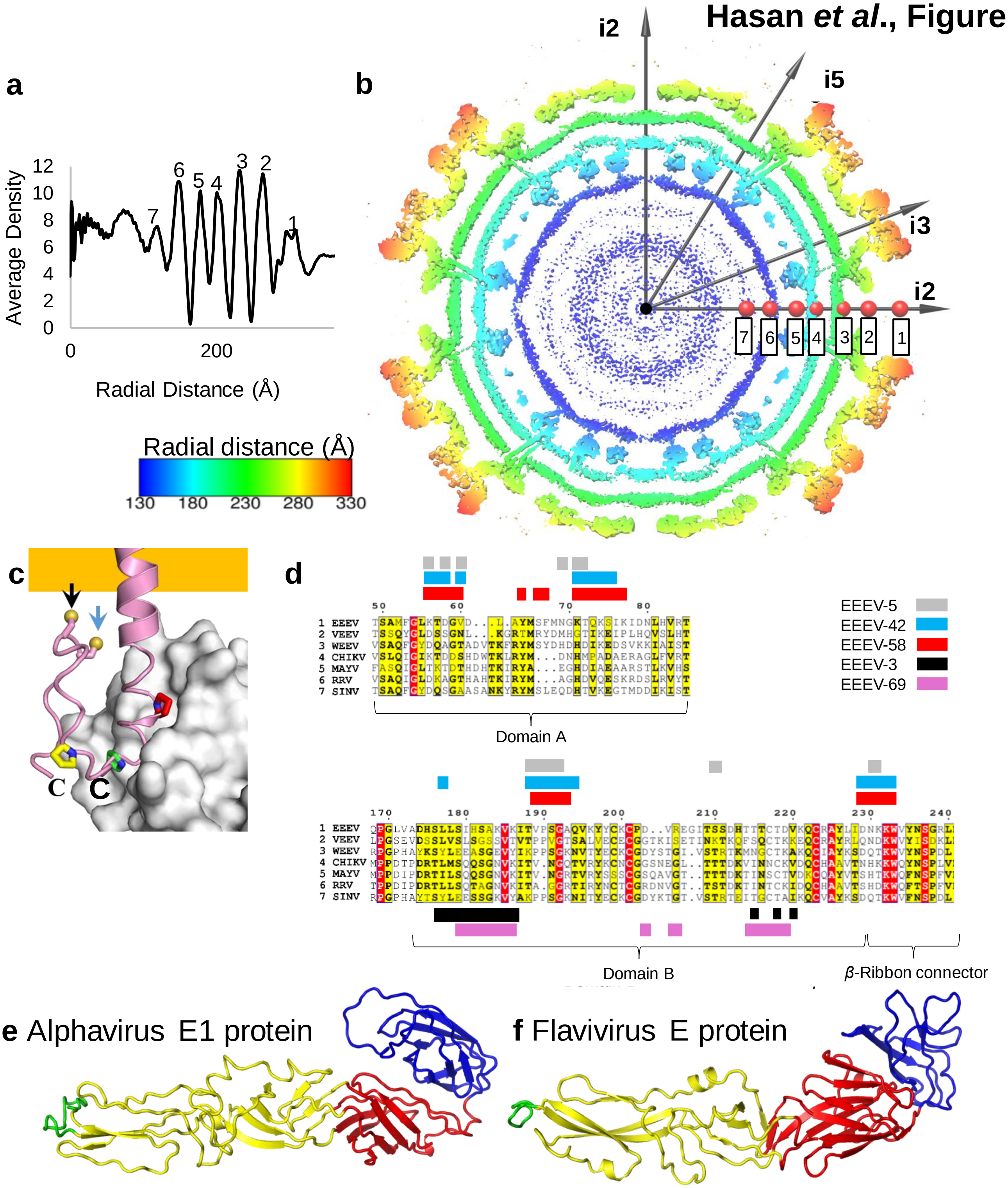

Figure S7. Structural features of EEEV, Related to **Figures 3, 4, 5, and 6**. (**a, b**) Radial density distribution of EEEV cryoEM map. (**a**) Trace of radial density. (**b**) Peak assignment: E2 ecto-domain (peak 1, r=300Å), base of E1-E2 spike (peak 2, r=263Å), outer-leaflet lipid bilayer head-groups (peak 3, r=232Å), inner-leaflet lipid bilayer head-groups (peak 4, r=200Å), capsid chymotrypsin-like C-terminal domain (peak 5, r=178Å), capsid RNA-binding N-terminal domain (peak 6, r=147Å), unidentified density (peak 7, r=119 Å). The peaks of radial density distribution are mapped as red spheres on a radially colored section of the EEEV cryoEM map. Icosahedral axes are shown as arrows. (**c**) Capsid-E2 interactions. The capsid C-terminal chymotrypsin-like domain (white surface) accommodates a turn at the E2 C-terminus (pink). Conserved E2 residues critical for the structure of the turn are shown (red, Pro396; green, Pro401; yellow, Pro406. The black and blue arrows point to the side-chains of Cys413 and Cys414 respectively, which face the membrane. (**d**) Footprints of individual Fabs are color-coded according to the key on the right. Red indicates complete conservation, yellow partial conservation and white no conservation of E2 residues. (**e**) EEEV E1 ecto-domain and (**f**) ZIKV E ecto-domain (PDB 5IRE). Color code: domain I, red; domain II, yellow; domain III, blue; fusion peptide, green.

SUPPLEMENTARY TABLES

Table S1. Summary of cryoEM data collection and processing, Related to **Figure 1**.

			EEEV-Fab Complex				
		EEEV	EEEV-3	EEEV-5	EEEV-42	EEEV-58	EEEV-69
Data Deposition	EMDB	9280	9274	9275	9249	9278	9279
	PDB ID	6MX4	6MW9	6MWC	6MUI	6MWV	6MWX
Microscope Settings	Dose ($e^-/Å^2$)	31	31	31	31	31	31
	Magnification (X)	18,000	18,000	18,000	18,000	18,000	18,000
	Pixel size (Å)	1.58	1.62	1.62	1.62	1.62	1.62
	Defocus range (μm)	-0.5 to -3.5	-1.5 to -5.0	-1.5 to -5.0	-1.0 to -3.5	-1.5 to -5.0	-1.0 to -3.5
Data	#Micrographs	2,416	937	765	570	900	579
	#Boxed particles	72,833	24,202	16,583	7,318	15,679	7,574
	#Particles after 2D classification	30,806	8,416	6,583	4,733	7,335	5,964
Resolution (Å)	FSC=0.5	5.3	8.9	9.4	9.7	8.8	10.6
	FSC=0.143	4.4	7.4	7.5	7.7	7.3	8.2

Table S2. Refinement statistics of E1-E2-capsid coordinates assuming four copies each of protein in the asymmetric unit, Related to **Figure 1**.

Ramachandran (favored+allowed)	99.6%
Ramchandran (outliers)	0.4%
Rotamers (favored + allowed)	100%
Rotamers (outliers)	0.0%
Cβ deviations	0
Molprobity all atom clash-score	11.0

Table S3. Isoelectric points of EEEV envelope proteins, Related to **Figure 5**.

	E1	E2
Full length	6.6	8.8
Ecto-domain	6.2	8.6
Binding site	4.6	10.2

Table S4. Isoelectric points (pI) of alphavirus envelope protein ecto-domains, Related to **Figure 5**.

Disease	Virus	E1 ecto-domain	E2 ecto-domain
		pI	pI
Encephalitis	EEEV	6.2	8.6
	WEEV	6.0	8.9
	VEEV	6.1	8.3
Arthritis	SINV	6.0	7.9
	CHIKV	6.1	7.6
	MAYV	6.7	8.0
	RRV	6.2	6.8

Table S5. Sumf values of E1-E2 domains in EEEV-Fab complex cryoEM maps, Related to **Figures 6** and **7**. The sumf values were averaged for four symmetry-related positions within the T=4 icosahedral asymmetric.

	Fab	E1 ecto-domain			E2 ecto-domain			
		I	II	III	*β*-Ribbon	A	B	C
Domain A	5	38.3	42.2	38.8	35.2	32.4	40.7	26.8
	42	38.7	39.9	41.1	32.5	31.2	41.0	28.7
	58	37.7	42.1	41.0	33.3	31.5	42.0	26.9
Domain B	3	36.4	41.3	40.6	37.9	35.4	42.6	26.9
	69	37.7	42.1	41.0	33.3	31.5	42.0	26.9

Table S6. Fabs: Occupancy and orientation, Related to **Figures 6** and **7**.

E2 Domain	A				A				A			
Fab	5				42				58			
Position	i1	q1	q2	q3	i1	q1	q2	q3	i1	q1	q2	q3
E1 ecto-domain	40.1	40.4	40.3	39.6	39.3	39.4	39.2	40.4	39.9	40.0	41.6	40.3
E2 ecto-domain	35.7	36.8	37.1	36.1	35.1	35.7	35.1	33.5	35.4	36.8	36.3	34.4
Fab	17.2	19.5	16.3	16.6	28.4	27.6	26.4	27.6	37.4	36.9	35.8	36.3
Occupancy (%)	45.4	50.5	42.1	43.9	76.3	73.5	71.1	74.7	99.3	96.1	91.9	97.2
Avg. Occupancy (%)	45.4				74.7				97.2			
Angle (°)	14.5				31.3				32.0			

E2 Domain	B				B			
Fab	3				69			
Position	i1	q1	q2	q3	i1	q1	q2	q3
E1 ecto-domain	38.8	39.2	39.7	40.8	41.6	41.4	40.4	41.7
E2 ecto-domain	37.3	38.4	39.2	38.2	40.7	40.9	38.6	38.1
Fab	34.5	34.2	34.1	34.1	37.1	34.0	33.6	34.5
Occupancy (%)	90.7	88.1	86.4	86.3	90.2	82.6	85.1	86.5
Avg. Occupancy (%)	88.8				87.5			
Angle (°)	21.1				52.0			

Table S7. Isoelectric points (pI) of flavivirus E protein ecto-domains, Related to **Figure 5**.

Flavivirus	pI
DENV1	6.5
DENV2	6.8
DENV3	6.1
DENV4	6.5
JEV	6.4
WNV	6.4
YFV_Asibi	5.8
ZIKV_HPF	6.1
