# Supplementary material for: Cryo-EM Structures of Eastern Equine Encephalitis Virus Reveal Mechanisms of Virus Disassembly and Antibody Neutralization
Source: Cell Rep. 2018 Dec 11;25(11):3136–3147.e5. doi: 10.1016/j.celrep.2018.11.067 (PMC6302666; doi:10.1016/j.celrep.2018.11.067)
Supplement: Document S2. Article plus Supplemental Information [file mmc2.pdf]

Cell Reports

Article

Cryo-EM Structures of Eastern Equine Encephalitis Virus Reveal Mechanisms of Virus Disassembly and Antibody Neutralization

Graphical Abstract

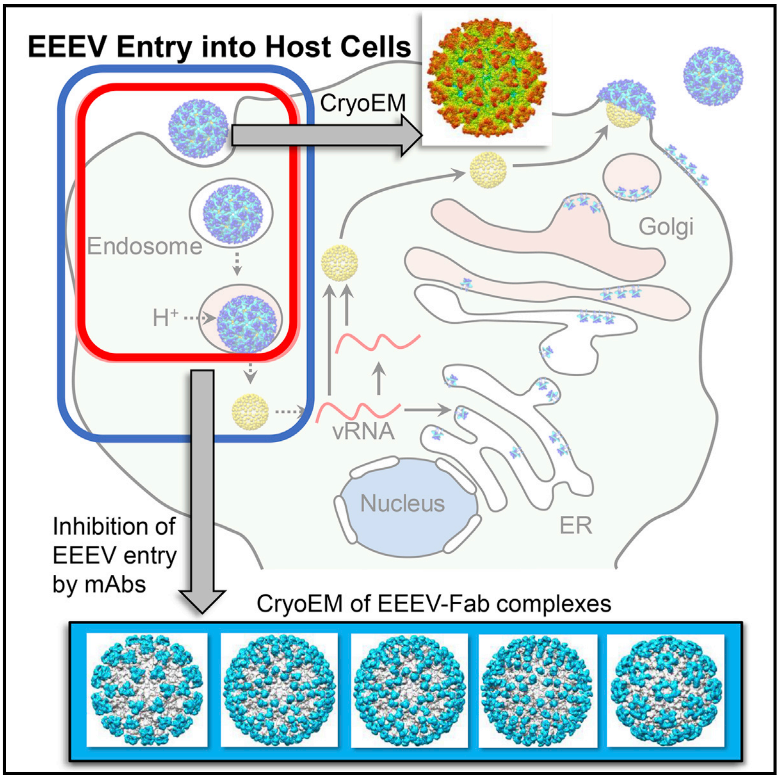

Highlights

- EEEV cryo-EM structure shows the basis of receptor binding and pH-triggered disassembly
- Cryptic envelope protein glycosylation interferes with immune detection
- EEEV RNA genome binding site on capsid protein has an extended conformation
- Antibody inhibition of EEEV entry involves cross-linking of viral envelope proteins

Authors

S. Saif Hasan, Chengqun Sun, Arthur S. Kim, ..., Michael S. Diamond, William B. Klimstra, Michael G. Rossmann

Correspondence

mr@purdue.edu

In Brief

Hasan et al. use single-particle cryoelectron microscopy to elucidate the molecular basis of host cell entry of neurovirulent EEEV. They show that the EEEV envelope is primed for intracellular pH sensing and subsequent disassembly. Monoclonal antibodies effectively inhibit EEEV entry by cross-linking the viral envelope.

Hasan et al., 2018, Cell Reports *25*, 3136–3147
December 11, 2018 © 2018 The Author(s).
https://doi.org/10.1016/j.celrep.2018.11.067

OPEN ACCESS
CellPress

Cell Reports
Article

Cryo-EM Structures of Eastern Equine Encephalitis Virus Reveal Mechanisms of Virus Disassembly and Antibody Neutralization

S. Saif Hasan,[1] Chengqun Sun,[2,3] Arthur S. Kim,[4,5] Yasunori Watanabe,[6,7,8] Chun-Liang Chen,[1] Thomas Klose,[1] Geeta Buda,[1] Max Crispin,[6] Michael S. Diamond,[4,5,9,10] William B. Klimstra,[2,3] and Michael G. Rossmann[1,11,*]
[1]Department of Biological Sciences, Purdue University, West Lafayette, IN 47907, USA
[2]Department of Immunology, University of Pittsburgh, Pittsburgh, PA 15261, USA
[3]Center for Vaccine Research, University of Pittsburgh, Pittsburgh, PA 15261, USA
[4]Department of Medicine, Washington University School of Medicine, St. Louis, MO 63110, USA
[5]Department of Pathology and Immunology, Washington University School of Medicine, St. Louis, MO 63110, USA
[6]Centre for Biological Sciences and Institute of Life Sciences, University of Southampton, Southampton SO17 1BJ, UK
[7]Oxford Glycobiology Institute, Department of Biochemistry, University of Oxford, Oxford OX1 3QU, UK
[8]Division of Structural Biology, University of Oxford, Oxford OX3 7BN, UK
[9]Department of Molecular Microbiology, Washington University School of Medicine, St. Louis, MO 63110, USA
[10]The Andrew M. and Jane M. Bursky Center for Human Immunology and Immunotherapy Programs, Washington University School of Medicine, St. Louis, MO 63110, USA
[11]Lead Contact
*Correspondence: mr@purdue.edu
https://doi.org/10.1016/j.celrep.2018.11.067

SUMMARY

Alphaviruses are enveloped pathogens that cause arthritis and encephalitis. Here, we report a 4.4-Å cryoelectron microscopy (cryo-EM) structure of eastern equine encephalitis virus (EEEV), an alphavirus that causes fatal encephalitis in humans. Our analysis provides insights into viral entry into host cells. The envelope protein E2 showed a binding site for the cellular attachment factor heparan sulfate. The presence of a cryptic E2 glycan suggests how EEEV escapes surveillance by lectin-expressing myeloid lineage cells, which are sentinels of the immune system. A mechanism for nucleocapsid core release and disassembly upon viral entry was inferred based on pH changes and capsid dissociation from envelope proteins. The EEEV capsid structure showed a viral RNA genome binding site adjacent to a ribosome binding site for viral genome translation following genome release. Using five Fab-EEEV complexes derived from neutralizing antibodies, our investigation provides insights into EEEV host cell interactions and protective epitopes relevant to vaccine design.

INTRODUCTION

Alphaviruses are arthropod-transmitted enveloped pathogens that cause epidemics in humans and other vertebrate animals (Jose et al., 2009; Schwartz and Albert, 2010; Strauss and Strauss, 1994). Alphaviruses have an ~12-kb unsegmented single-stranded (+)RNA genome that encodes four non-structural and five structural proteins (Strauss and Strauss, 1994). The icosahedral shell of alphaviruses consists of an outer layer of trans-membrane envelope E1 and E2 proteins and an inner capsid layer separated by a host-derived membrane. Previous cryoelectron microscopy (cryo-EM) studies of chikungunya (CHIKV), Semliki Forest (SFV), Sindbis (SINV), Ross River (RRV), Venezuelan (VEEV), and western equine encephalitis (WEEV) viruses have shown that the E1 and E2 proteins are organized into 20 icosahedral 3-fold and 60 quasi-3-fold trimeric spikes (Kostyuchenko et al., 2011; Mancini et al., 2000; Mukhopadhyay et al., 2006; Sherman and Weaver, 2010; Smith et al., 1995; Sun et al., 2013; Zhang et al., 2002, 2005, 2011). Crystallographic structures of the E1 and E2 ectodomains and the capsid C-terminal domain (CTD) also have been determined for several alphaviruses (Choi et al., 1991; Gibbons et al., 2004; Lescar et al., 2001; Li et al., 2010; Voss et al., 2010). The capsid N-terminal domain (NTD) is disordered and binds the negatively charged alphavirus RNA genome (Owen and Kuhn, 1996).

Alphaviruses utilize the E2 protein for attachment to incompletely characterized receptors (Schwartz and Albert, 2010; Zhang et al., 2018). Alphaviruses are internalized by endocytosis (Figure S1). Endosome acidification triggers conformational changes in the E1 and E2 proteins that generate the fusogenic conformation of the E1 protein (Gibbons et al., 2004; Haag et al., 2002). Viral-endosomal membrane fusion is followed by the release of the nucleocapsid (NC) core into the host cytosol for initiation of viral replication (Haag et al., 2002).

Structural investigations of alphaviruses have concentrated mainly on arthritogenic alphaviruses (Kostyuchenko et al., 2011; Mukhopadhyay et al., 2006; Smith et al., 1995; Sun et al., 2013; Tang et al., 2011; Zhang et al., 2002, 2005). In contrast, structural information on encephalitic alphaviruses is limited (Porta et al., 2014; Sherman and Weaver, 2010; Zhang et al., 2011). Encephalitic alphaviruses are considered potential biological weapons, as virus particles can be dispersed as aerosols to

 © 2018 The Author(s).
This is an open access article under the CC BY license (http://creativecommons.org/licenses/by/4.0/).

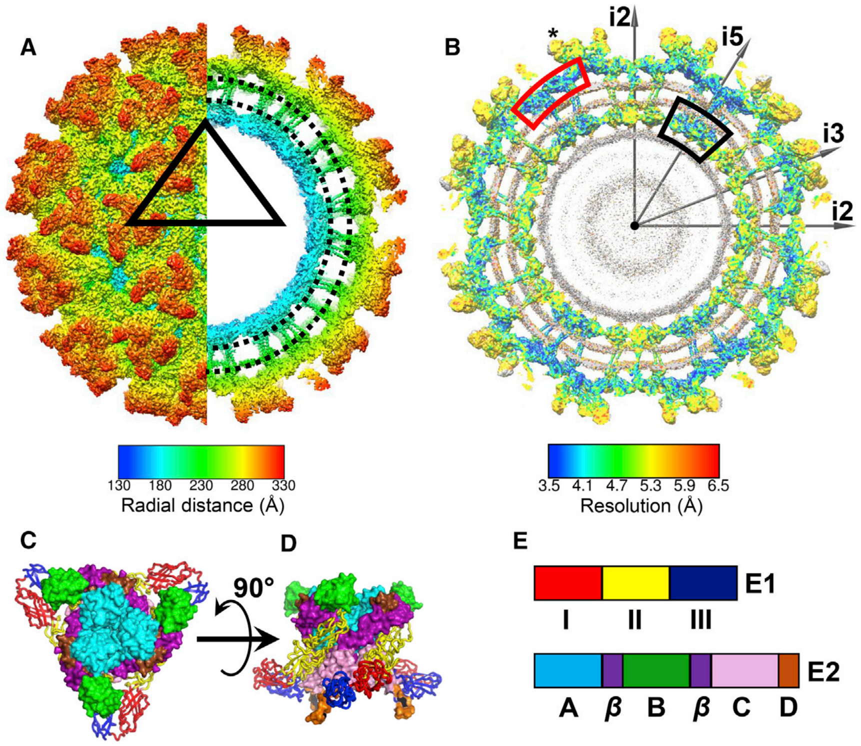

Figure 1. Cryo-EM Structure of EEEV

(A) Radially colored surface representation of EEEV. The right half shows a section of the map (lipid bilayer between the black dotted lines).

(B) Resolution distribution in the cryo-EM map according to the scale provided at the bottom. The RNA genome (gray) is excluded from the resolution scale. Gray arrows: directions of icosahedral symmetry axes. Red box, E1 ectodomain; black box, capsid proteins near icosahedral 5-fold axis; asterisk, E2 ectodomain.

(C–E) Trimeric E1-E2 spikes shown in (C) a radial orientation and (D) rotated by 90°. E1 and E2 are shown using ribbon and surface representation, respectively. (E) Domain distribution in E1 and E2 protein ecto-domains. The color codes follow domain distribution as indicated in (E).

See also Figures S1–S3.

initiate infections (Roy et al., 2009). Severe neurological disease is associated with infections of eastern equine encephalitis virus (EEEV), which causes up to 70% fatality rates in symptomatic cases (Armstrong and Andreadis, 2013; Villari et al., 1995). Outbreaks of EEEV have been reported in recent years in the eastern parts of the United States and in Panama (Carrera et al., 2013; Silverman et al., 2013). To gain insight into the molecular organization of encephalitic alphaviruses, we determined a cryo-EM structure of an EEEV virion derived from a SINV-EEEV chimeric virus to a resolution varying from 3.5 to 6.5 Å, corresponding to an average resolution of 4.4 Å. This structure provides information about EEEV entry into host cells (steps 1–4 in Figure S1). Structures of previously reported sequences of a genome binding site (Owen and Kuhn, 1996) and a ribosome binding site (RBS) (Wengler et al., 1992) were observed on the capsid protein. The EEEV cryo-EM map also revealed a binding site for heparan sulfate (HS), which has been linked to viral neurovirulence and avoidance of lymphotropism (Gardner et al., 2011, 2013). The cryo-EM analysis of EEEV, quantitative glycan analysis, and virus internalization assays provide mechanistic insights into an evasion mechanism by which EEEV inefficiently enters into myeloid lineage cells including macrophages and dendritic cells. Cryo-EM structures of EEEV complexed with Fab fragments from five potently neutralizing monoclonal antibodies (mAbs) provided insights into host cell entry and neutralization.

RESULTS

The EEEV cryo-EM map shows overall conservation of structural features among alphaviruses (Figures 1A, 1B, S2, S3A–S3D; Tables S1 and S2). The E1 ectodomain is divided into domains I, II (responsible for fusion), and III. The E2 ectodomain consists of domains A (putative receptor-binding), B (putative receptor-binding and protection of fusion loop), C, D, and a β-ribbon connector (Li et al., 2010; Voss et al., 2010) (Figures 1C–1E). The EEEV E1 and E2 ectodomains are stabilized by disulfide bonds (Figures S3E and S3F). The E1 and E2 ectodomain are resolved to a resolution of ~3.5–4.0 and ~4.0–5.5 Å, respectively (Figure 1B), with the relatively poor resolution of the latter due to potential flexibility. The capsid molecules near icosahedral 5-fold axes have a resolution of ~3.5 Å. In contrast, capsid molecules positioned near other sites have a relatively poor resolution.

HS Binding Motif

The HS binding phenotype of EEEV is observed in wild-type strains and is not an artifact of cell culture adaptation (Gardner et al., 2011, 2013). EEEV neurovirulence, HS binding phenotype, and avoidance of lymphotropism are linked to three basic residues, Lys71, Lys74, and Lys77 (‘‘Lys-triad’’), on the E2 ectodomain (Gardner et al., 2011, 2013). Here, these three E2 Lys residues are part of an exposed β-strand and loop on the surface of domain A (Figures 2A and 2B). Lys77 is located closest to the trimeric spike 3-fold axis. The Cα-atoms of adjacent Lys residues are separated by ~10 Å and form a linear binding site for HS. The symmetry-related Cα-atoms of the three Lys77 residues on a trimeric spike are separated by a distance of ~25 Å. Among other alphaviruses, none of which interact with HS with the efficiency of EEEV (Gardner et al., 2011), Lys74 is most conserved, Lys77 is least conserved, and Lys71 is often replaced by His (Figure S4). The residue at E2 position 75 is acidic in all alphaviruses except EEEV (Figure S4).

Envelope Protein Glycosylation

E1-E2 N-linked glycans are potential binding sites for cell surface lectins such as DC-SIGN (dendritic cell-specific intercellular

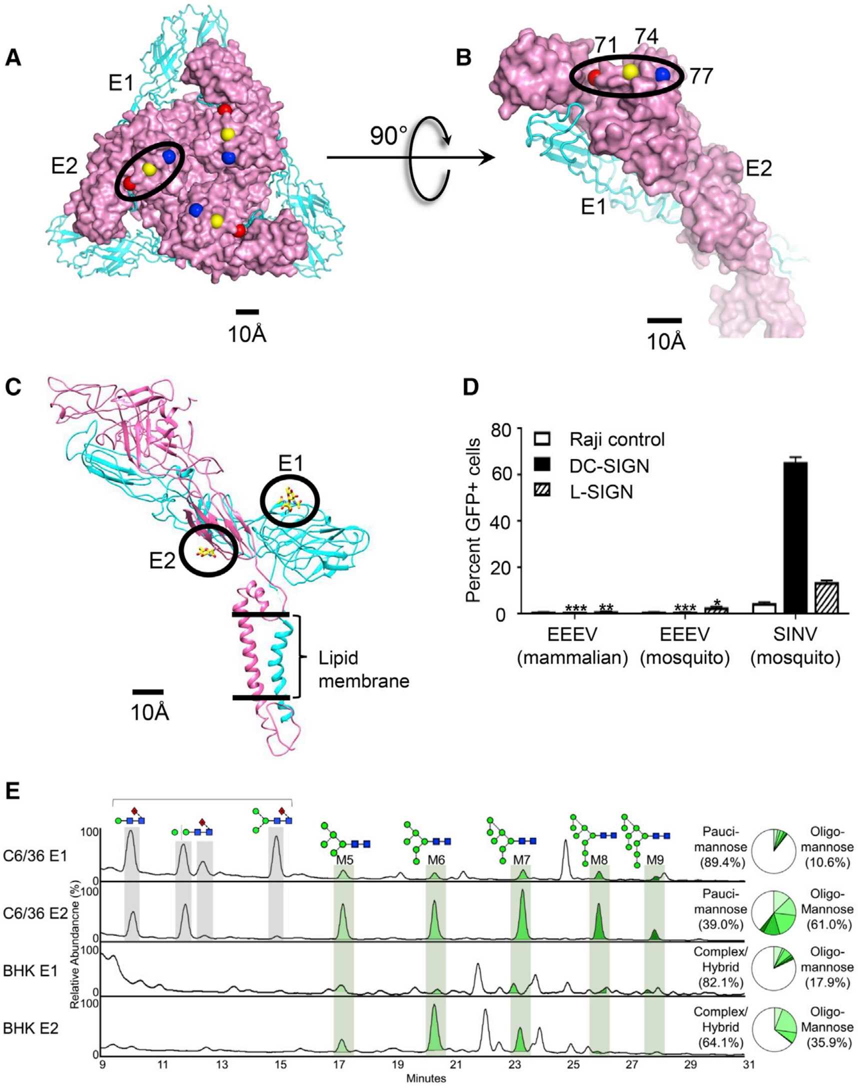

Figure 2. Putative Receptor-Binding Sites on the E1 and E2 Proteins

(A) The Cα-atoms of the HS binding Lys residues are shown as spheres (Lys71, red; Lys74, yellow; Lys77, blue) on the E2 ectodomain (pink). One of the three symmetry-related triads in a trimeric spike is marked in a black oval.

(B) HS binding residues in one E1-E2 heterodimer.

(C) N-linked glycosylation sites on E1 (cyan) and E2 (pink) ectodomains, highlighted in black circles.

(D) Assay of EEEV infection in Raji cells expressing DC-SIGN and L-SIGN. Infection of L-SIGN expressing Raji cells by EEEV derived from mosquito cells was significantly greater (two-way ANOVA with Tukey's multiple-comparison test; $^{*}p < 0.0001$) than infection of the Raji control cells, whereas infection with mammalian cell-derived virus was not ($^{**}p > 0.05$). Mosquito cell and mammalian cell-derived EEEV infection rates were not significantly higher for DC-SIGN-expressing cells than control cells ($^{***}p > 0.05$). SINV is used as a positive control for host cell entry. Three replicates were used for each virus and cell type, and two independent experiments were performed. The error bars represent SD.

(E) Quantitative and compositional analysis of EEEV glycosylation. HILIC-UPLC profiles of fluorescently labeled N-linked glycans from EEEV-derived E1 and E2 glycoproteins from C6/36 and BHK-15 cells. Oligomannose-type glycans (M5-M9; $Man_5GlcNAc_2$-$Man_9GlcNAc_2$) (green) were identified by Endo H digestion with quantification of major glycan types summarized in the pie charts.

See also Figures S4–S6.

adhesion molecule-3-grabbing non-integrin) and L-SIGN (liver/lymph node-specific intercellular adhesion molecule-3 grabbing non-integrin) (Klimstra et al., 2003). DC-SIGN is expressed in lymphoid dendritic cells and macrophages, whereas L-SIGN is expressed in endothelial cells in lymph nodes, liver sinusoids, and the placenta (Lozach et al., 2007). N-linked glycans are attached to EEEV E1 Asn134 and E2 Asn315, which are each part of the glycosylation motifs Asn134-Ile135-Thr136 and Asn315-Phe316-Thr317, respectively (Figures 2C, S4, and S5). The E1 glycan is accessible on the EEEV surface close to the icosahedral 2-fold and 5-fold vertices (Figures S6A and S6B). However, the E2 glycan is not exposed (Figure 2C). The EEEV cryo-EM map accommodates an *N*-acetyl-glucosamine monosaccharide at the E2 site and a disaccharide at the E1 site (Figure 2C). Of note, at least one glycosylation site on the E2 protein is accessible on the surface of all alphaviruses examined here except for EEEV (Figure S6C).

To evaluate whether EEEV interacts with DC-SIGN or L-SIGN possibly through the exposed E1 glycan, a flow cytometric assay was performed using Raji B lymphoblast cells ectopically expressing DC-SIGN or L-SIGN (Figure 2D). Expression of DC-SIGN and L-SIGN on the surface of Raji cells was previously shown to allow alphavirus binding, entry, and replication in these otherwise receptor-deficient cells (Klimstra et al., 2003). We observed that EEEV was unable to efficiently infect with Raji cells expressing DC-SIGN or L-SIGN as compared to SINV, the positive control (Figure 2D). Thus, unlike other alphaviruses, neurotropic EEEV has limited interactions with DC-SIGN and L-SIGN.

DC-SIGN and L-SIGN have been shown to interact most efficiently with high-mannose carbohydrate modifications, which are a characteristic feature of invertebrate glycosylation pathways including those in arbovirus mosquito vectors (Crispin et al., 2014; Mitchell et al., 2001). To evaluate the carbohydrate composition of the EEEV E1 and E2 glycans, a comparative hydrophilic interaction chromatography ultra-performance liquid chromatography (HILIC-UPLC) analysis was performed using virus cultivated in *Aedes albopictus* C6/36 or mammalian BHK-15 cells (Figure 2E). The exposed E1 glycan consisted of predominantly pauci-mannose carbohydrates in the C6/36 cell-derived virus and complex-type carbohydrates in the BHK-15 cell-derived virus, but not oligo-mannose glycans required for

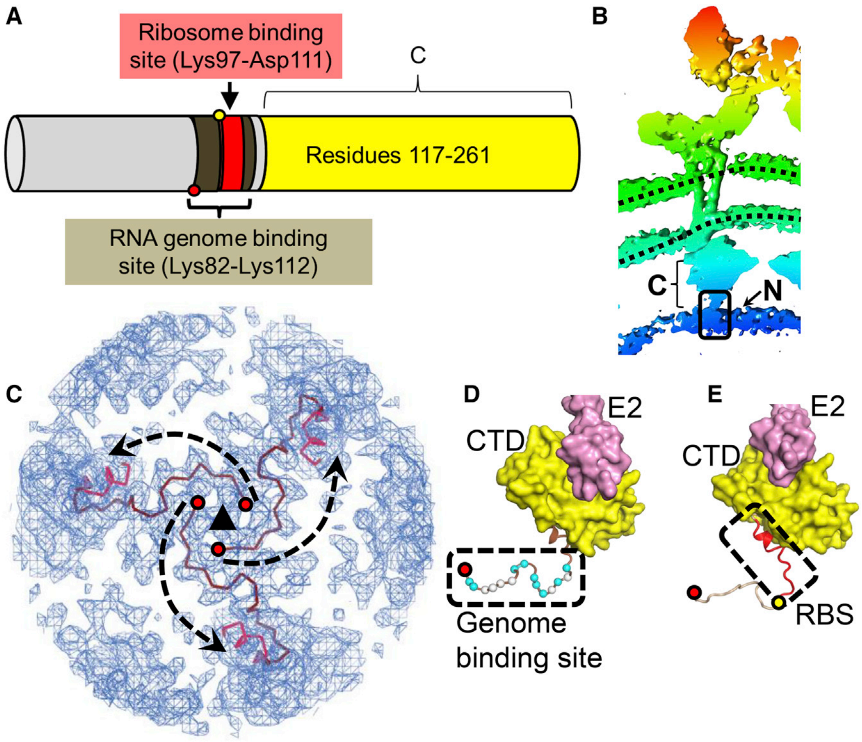

Figure 3. Structure of the EEEV Capsid Protein

(A) NTD and CTD are shown in gray and yellow, respectively.

(B) Map around one E1-E2-capsid trimer (radial coloring according to scale in Figure 1A; dotted lines: viral membrane). Capsid NTD and CTD are labeled "N" and "C," respectively. Black box, depth of region shown in (C).

(C) Genome binding residues Lys82-Lys112 are shown as extended brown chains. The Lys82 Cα-atom is shown in (C)–(E) to mark the N terminus of the main-chain trace of the capsid genome binding site.

(D) Capsid genome binding site (within a black box) and CTD (yellow surface). Red circle, Lys82 Cα-atom; cyan and white circles, Cα-atoms of basic and Pro residues, respectively; pink, E2 endodomain.

(E) RBS is shown as a red ribbon enclosed in a box. Yellow circle, Lys97 Cα-atom (see A for reference).

See also Figure S7.

efficient interactions with DC-SIGN and L-SIGN. Therefore, even though EEEV contains an exposed glycan on the E1 ectodomain, the carbohydrate composition of the invertebrate-derived glycan at this site does not favor interactions with these lectins.

Interactions of EEEV Membrane with Receptors

A third potential alphavirus receptor is the mucin TIM-1, likely because of its ability to bind phosphatidylserine (PS) lipids (Jemielity et al., 2013; Moller-Tank et al., 2013). The EEEV membrane is accessible at the icosahedral 2- and 5-fold vertices (Figures S6D and S6E), which is a conserved structural feature of alphaviruses and presents potential lipid interaction sites with host TIM1. As the diameter of the TIM1 lipid-binding IgV domain is ~26 Å (PDB ID 5DZO [Yuan et al., 2015]), this receptor could be accommodated near the exposed EEEV membrane at the icosahedral 2-fold vertices; here, the hole exposing the viral membrane has an approximately elliptical shape and a diameter of ~32 Å along the shorter elliptical axis. In contrast, TIM1-viral membrane interactions at the icosahedral 5-fold vertices (hole diameter, ~23 Å) would require conformational changes in the E1 ectodomains near the 5-fold axes and possibly in the TIM1 receptor.

Integrin Binding Sites

Integrins are membrane proteins involved in cellular adhesion (Ruoslahti, 1996). Two integrin-binding motifs are found in the EEEV E2 protein sequence, i.e., an RGD (Arg37-Gly38-Asp39) and a PPG (Pro104-Pro105-Gly106) motif in the E2 ectodomain (Figure S6F), and one motif is found in the E1 ectodomain sequence, i.e., a KGD motif (Lys378-Gly379-Asp380) (Figure S6F). The Pro104-Pro105-Gly106 motif also is found in the E2 protein of encephalitic WEEV and the arthritogenic Mayaro virus (MAYV), RRV, and SINV (Figure S4). All three motifs have been implicated in interactions of viruses with integrins (Chen et al., 2012; La Linn et al., 2005; Mason et al., 1994). The PPG motif also was suggested to be involved in alphavirus interactions with an integrin (La Linn et al., 2005). Of the three integrin binding sites described here, the PPG site is most accessible on the viral surface whereas the RGD site is least exposed as it is located at the E1-E2 interface. The KGD motif is located close to the E1-E1 interface near the icosahedral 2- and 5-fold vertices. However, direct binding interactions of alphaviruses to integrins have yet to be demonstrated.

Structure of the Capsid Protein

The EEEV capsid protein consists of two domains: NTD, residues 1–116, and CTD, residues 117–261 (Figures 3A and 3B). The structure of the alphavirus capsid CTD, which has a chymotrypsin-like fold, has been determined by crystallography (Choi et al., 1991) and cryo-EM (Zhang et al., 2011). The capsid residues Lys81-Arg114 in SINV (Owen and Kuhn, 1996) (corresponding to EEEV capsid Lys82-Lys112) have been implicated in interactions with the RNA genome (Figure 3A). Despite the identification of the NTD genome-binding sequence on the capsid protein more than 20 years ago (Owen and Kuhn, 1996), the structure of this domain has remained elusive, probably because the capsid NTD sequence (Met1-Ile116 in EEEV) shows features characteristic of intrinsically disordered proteins (Uversky, 2013) with high concentrations of basic residues Arg and Lys (27% of the sequence) and structure-disrupting Pro and Gly residues (26% of the sequence).

In the EEEV cryo-EM map, the main-chain coordinates of the genome binding capsid protein residues Lys82-Lys112

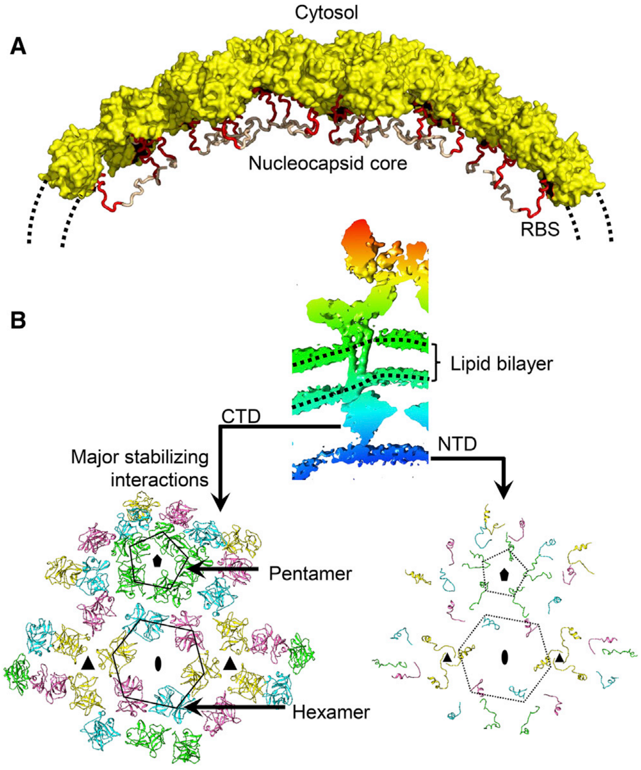

Figure 4. Organization of the Nucleocapsid Core

(A) Core surface formed by capsid CTD (yellow). RBSs are colored red.

(B) Organization of capsid double-layered shell. Top: cryo-EM map around one E1-E2-capsid trimer (radial coloring according to the scale in Figure 1A; dotted black lines, viral membrane). Bottom panels, left: Pentamers and hexamers of capsid CTD near the icosahedral 5- and 2-fold axes, respectively. No capsid-capsid contacts are observed near the icosahedral 3-fold axes. Right: Capsid genome binding segment. Capsid-capsid contacts are only near the icosahedral 3-fold.

See also Figure S7.

were observed to be adjacent to the capsid CTD (Figure 3C). The residues Lys82-Gly99 form an extended network underneath the capsid CTD (Figures 3C and 3D). These residues form an extended coil that contains a high concentration of basic residues and structure-disrupting Pro residues (Figure 3D).

An analysis of the EEEV cryo-EM map at lower contour levels shows multiple conformations of the capsid N-terminal chain, although noise in the map does not allow the main-chain atoms of these other conformations to be traced. In a previous low-resolution reconstruction of SINV, the section of the map corresponding to the Lys82-Gly99 region described here (Figures S7A and S7B) was interpreted to represent genomic RNA and not the capsid NTD (Zhang et al., 2002). The current map of EEEV confirms that the density into which Lys82-Gly99 has been traced is indeed a part of the capsid protein.

The alphavirus capsid protein binds to ribosomes during NC disassembly in the cytosol (Wengler et al., 1992). In EEEV, the residues constituting the RBS (Lys97-Asp111) form a coil and a short helix (Figure 3E). The RBS was located on the inside of the intact NC cores (Figure 4A). This implies that exposure of the RBS requires at least a partial dissociation of the icosahedral capsid shell. The icosahedral capsid shell in EEEV consists of an outer layer formed by the chymotrypsin-like CTD and an inner layer formed by the extended NTD (Figure 4B). The capsid-capsid contacts in the outer capsid layer occur around the icosahedral 2-fold and 5-fold vertices and not around the 3-fold vertices (Figure 4B). Two pairs of residues involved in electrostatic interactions were observed at the capsid-capsid interface in the outer CTD layer: Lys171-Glu233 and Asp173-Arg236. The outer NC layer is stabilized by interactions with the E2 protein endodomains (Figure S7C).

Electrostatic Interactions at the E1-E2 Dimer

Endosome acidification during alphavirus entry triggers the dissociation of the E1-E2 dimer (Haag et al., 2002). The E1-E2 dimer interface has a concentration of complementary acidic residues on E1 and basic residues on E2 ectodomains (Figure 5A; Table S3). This pH-responsive complementary charged character of E1 and E2 proteins is conserved among alphaviruses (Table S4).

Formation of Fusogenic E1 Trimers

In the acidic endosomal environment, E1-E2 dimer is followed by the formation of fusogenic E1 trimers, which requires displacement of the E2 ectodomains away from the 3-fold axis of each trimeric spike (Haag et al., 2002). In EEEV, the E2-E2 interface formed by the three E2 ectodomains of each trimeric spike is enriched in pH-responsive basic residues (Figures 5B–5D), which show limited sequence conservation among alphaviruses (Figure S4).

Inhibition of EEEV Entry by mAbs

The entry-related steps in the alphavirus replication cycle can be exploited as targets for neutralization of infection by mAbs. Hence, a structural analysis was performed of EEEV complexed with Fab fragments derived from five potently neutralizing entry-inhibiting anti-EEEV mouse mAbs, whose production and neutralization characteristics are described elsewhere (Kim et al., 2018).

The binding footprints of Fab fragments of five neutralizing mAbs (EEEV-3, EEEV-5, EEEV-42, EEEV-58, and EEEV-69) were mapped using cryo-EM structures (Figure 6). EEEV-5, EEEV-42, and EEEV-58 Fabs bound to domain A of E2 utilizing primarily polar interactions (Figure S7D). EEEV-5 and EEEV-42 Fabs interacted with Lys71 and Lys74, two of the three HS binding residues (Gardner et al., 2011), whereas EEEV-58 interacted only with Lys74 of the three HS binding residues (Figure S7D). These three Fabs also make a few interactions with residues in domain B and the β-connector (Figure S7D). In comparison, Fab fragments of EEEV-3 and EEEV-69 interacted exclusively with residues in domain B (Figure S7D). Domains A and B were previously implicated in host cell attachment and pH-triggered conformational changes (Li et al., 2010; Voss et al., 2010). The E2 residues that comprise the footprints of these anti-EEEV mAbs had only limited sequence conservation with other alphaviruses (Figure S7D). Consistent with this

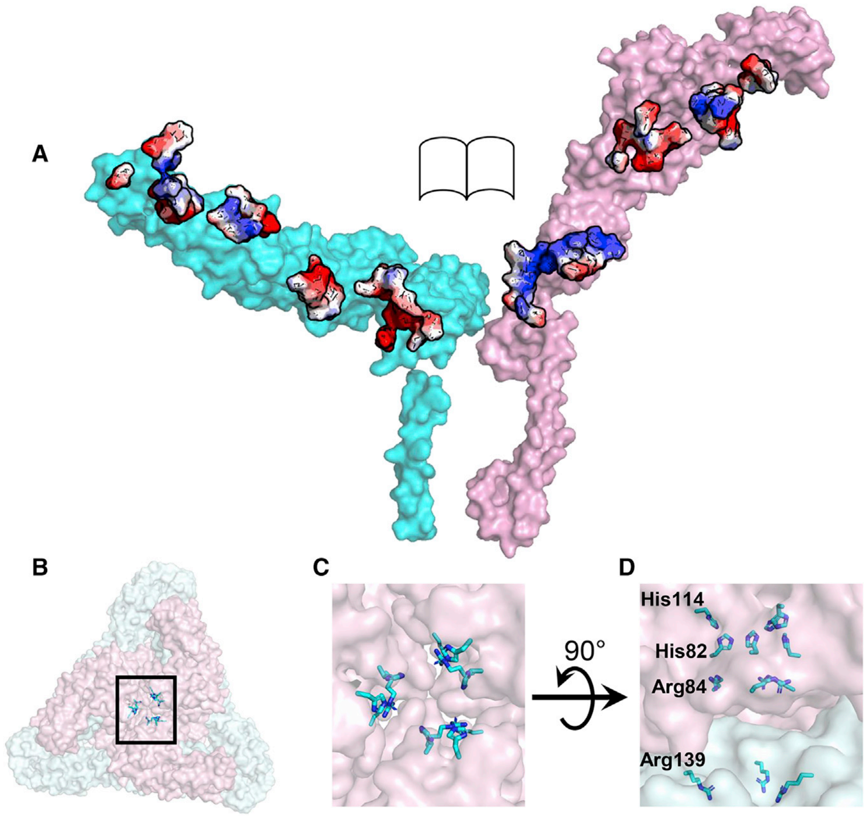

Figure 5. Charged Residues at the E1-E2 Interface

(A) Open book view of E1 (cyan) and E2 (pink) proteins showing complementary charged residues at the E1-E2 dimer interface. Acidic and basic residues are colored red and blue, respectively.

(B–D) Basic residues in the E2-E2 interface formed by three symmetry-related E2 ectodomains (pink), along the spike 3-fold axis.

(B) Trimeric spike along 3-fold axis. The black box encloses the basic residues in the E2-E2 interface.

(C) Magnified view of the black box from (B).

(D) The four basic residues along the E2-E2 interface from three symmetry-related E2 molecules.

See also Figures S4 and S7.

observation, the neutralizing activity of these mAbs does not extend to VEEV and WEEV, the related encephalitic alphaviruses (Kim et al., 2018).

Monovalent Fabs can achieve neutralization of alphavirus infections by either interfering with receptor binding to domain A or by clamping domain B in its neutral-pH conformation, which inhibits subsequent pH-triggered conformational changes. The stabilization of domains A and B can be assessed by calculating the average density of the fitted domain atoms ("sumf" [Rossmann et al., 2001]). In the EEEV-Fab cryo-EM maps (Table S5), the sumf values showed no significant stabilization of either domain A or B upon Fab binding. Indeed, the Fab fragments of the five anti-EEEV mAbs did not inhibit EEEV infection efficiently as confirmed by neutralization assays, which suggests that a bivalent, cross-linking activity may be required to achieve optimal inhibition (Figures 7A and 7B) (Edeling et al., 2014).

Fab occupancies were determined by comparing sumf values (see above for definition) of fitted Fab coordinates with the sumf values of the fitted E1-E2 ectodomains assuming that the ectodomains are present in the virus at 100% occupancy (Table S6). The EEEV-5 Fab had the lowest average occupancy of 45%, whereas the EEEV-58 Fab had the highest average occupancy of 97% (Table S6). The EEEV-Fab complex cryo-EM structures also show diversity in Fab orientations (Figure 6; Table S6). EEEV-5 is the most radial in orientation of the five Fab fragments and forms an angle of 14.5° with the spike 3-fold axis, whereas B domain-specific EEEV-69 is the most tangential with an orientation angle of 52.0° with the spike 3-fold axis.

DISCUSSION

The overall cryo-EM structure of EEEV described in this investigation is analogous to a previously published VEEV cryo-EM structure (Zhang et al., 2011). However, the present investigation identified features related to EEEV entry and disassembly including the structure of the capsid genome binding segment for which no structural information was previously available.

Receptor Binding Motifs: Implications for Host Cell Binding

Site-directed mutagenesis of EEEV Lys-triad residues to neutral Ala reduced HS dependence of EEEV infection by ~90%, indicating that the three E2 Lys residues, especially positions 71 and 74, have major roles in HS binding (Gardner et al., 2011, 2013). Our study showed that these three EEEV E2 Lys residues form a β-strand close to the 3-fold axis of each trimeric spike. Three symmetry-related copies of this HS binding Lys-triad are exposed on the surface of each trimeric spike. As HS is an extended polymer (Fuster and Wang, 2010), it is possible that the three symmetry-related Lys-triad sites on a trimeric spike are simultaneously engaged in high-avidity HS binding.

Previous investigations have correlated the lack of myeloid cell infectivity of EEEV to a micro-RNA-based translation-inhibition mechanism that suppresses replication of internalized EEEV genomes (Trobaugh et al., 2014). This mechanism circumvents immune system activation that is otherwise a common feature of other alphavirus infections (Trobaugh et al., 2014). We show here that lack of exposure of the E2 high-mannose glycan on the viral surface results in limited lectin-dependent infection of EEEV in cells expressing DC-SIGN or L-SIGN, which includes myeloid cells of the immune system. Along with HS binding, which reduces virus access to lymphoid tissues (Gardner et al., 2011), this lack of lectin-dependent cell entry may be utilized

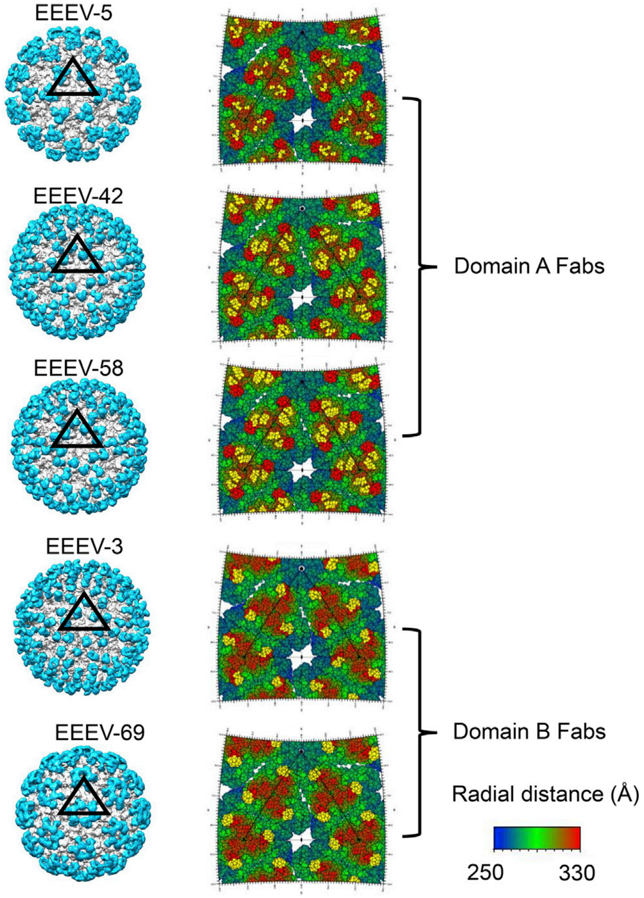

Figure 6. Cryo-EM Maps of EEEV-Fab Complexes
The Fabs are bound to E2 domains A and B. Color scheme: Fabs, cyan; E1-E2, white. Right side: Radially colored road-maps of the EEEV surface show contact residues (yellow) constituting the Fab footprint.
See also Figures S4 and S7.

by EEEV to further suppress myeloid cell infection and, together with micro-RNA-mediated inhibition, drive the extreme neurovirulence in mammals.

Another putative mechanism by which alphaviruses interact with host cell membranes is through the viral lipid bilayer, which can bind with TIM1 membrane protein that binds PS lipid (Jemielity et al., 2013). PS is exposed on the outer plasma membrane leaflet upon the induction of apoptosis (Leventis and Grinstein, 2010). Alphavirus infections cause apoptosis of host cells (Levine et al., 1993). Progeny alphavirus particles that bud from the mammalian cell plasma membrane could expose PS on the exposed outer leaflet of the viral membrane. Indeed, PS has been reported in alphavirus membranes (Hirschberg and Robbins, 1974; Laine et al., 1972). Here, it was inferred that TIM1-alphavirus interactions would be favored at the icosahedral 2-fold vertices compared to the 5-fold vertices as the diameter of the holes in the alphaviral envelope is large enough at the 2-fold vertices to accommodate the lipid-binding domain of TIM1. Whether interactions of alphaviruses with TIM1 involve only the lipid membrane or also the envelope proteins is currently not known.

Electrostatic Interactions at the E1-E2 Dimer Interface: Relevance to Alphavirus Disassembly

Endosome acidification triggers E1-E2 dimer dissociation in internalized alphavirus particles, resulting in viral-endosomal membrane fusion (Wahlberg et al., 1992). The EEEV E1-E2 dimer interface is formed by the acidic E1 and basic E2 proteins. Acidic pH in the endosome can neutralize the negatively charged E1 residues, thereby decreasing dimer stability. As the charged character of E1-E2 proteins is conserved among alphaviruses, this pH-dependent dimer dissociation mechanism is likely to be common to the life cycle of alphaviruses.

Low-pH-induced E1-E2 dimer dissociation is accompanied by trimeric spike disruption. As reported here, the E2-E2 interface along the 3-fold axis of each trimeric spike is enriched in basic residues. Acidic pH would protonate these basic residues, leading to electrostatic repulsion and trimer dissociation. The limited sequence conservation of these basic residues might explain variations in pH requirements for fusion of different alphaviruses in either early or late endosomes (van Duijl-Richter et al., 2015). An estimation of the quantitative contribution of each charged amino acid to the pH-triggered structural changes would require a comprehensive residue-by-residue mutagenesis analysis.

Low pH in the endosome lumen triggers the exposure of the E1 fusion loop peptide. The purified E1 ectodomain is stable as a monomer under neutral pH conditions without the need for stabilizing detergent or lipid (Klimjack et al., 1994; Wahlberg et al., 1992). The crystal structural of the E1 ectodomain showed an exposed fusion loop in the absence of stabilizing detergents or lipids (Lescar et al., 2001). This implies that the exposure of the fusion loop peptide is not sufficient for membrane fusion. Low pH has been implicated in the generation of a fusogenic state of the E1 ectodomain independent of its association with the E2 ectodomain (Klimjack et al., 1994). This E1 fusogenic state then results in the insertion of the E1 fusion loop peptide and additional portions of the E1 ectodomain into the host membrane (Gibbons et al., 2003). As reported here, the alphavirus E1 ectodomain has a conserved acidic character. Neutralization of acidic residues has been reported to serve as a mechanism of enhancement of the hydrophobicity of acidic proteins, which promotes interaction with membranes (Barrera et al., 2011). The conserved acidic character of the E1 ectodomain may provide a mechanism for the insertion of portions of the E1 ectodomain, including the fusion loop, into lipid membranes. In fact, residues outside the E1 fusion loop have been implicated in interactions with membranes indicating that other portions of the E1 ectodomain are also involved in membrane interactions (Chatterjee et al., 2002; Vashishtha et al., 1998).

The assembly and fusion of alphaviruses and flaviviruses in low-pH environments show similarities. The E3 protein prevents alphavirus E1-E2 dimer dissociation during assembly in the low-pH trans-Golgi network (Voss et al., 2010; Zhang et al., 2011). Analogously, the flavivirus prM protein stabilizes flavivirus envelope (E) proteins during assembly and maturation in the acidic Golgi environment (Guirakhoo et al., 1992). Functional similarities are also inferred from homologous structures of alphavirus E1 fusion protein and the flavivirus E fusion protein

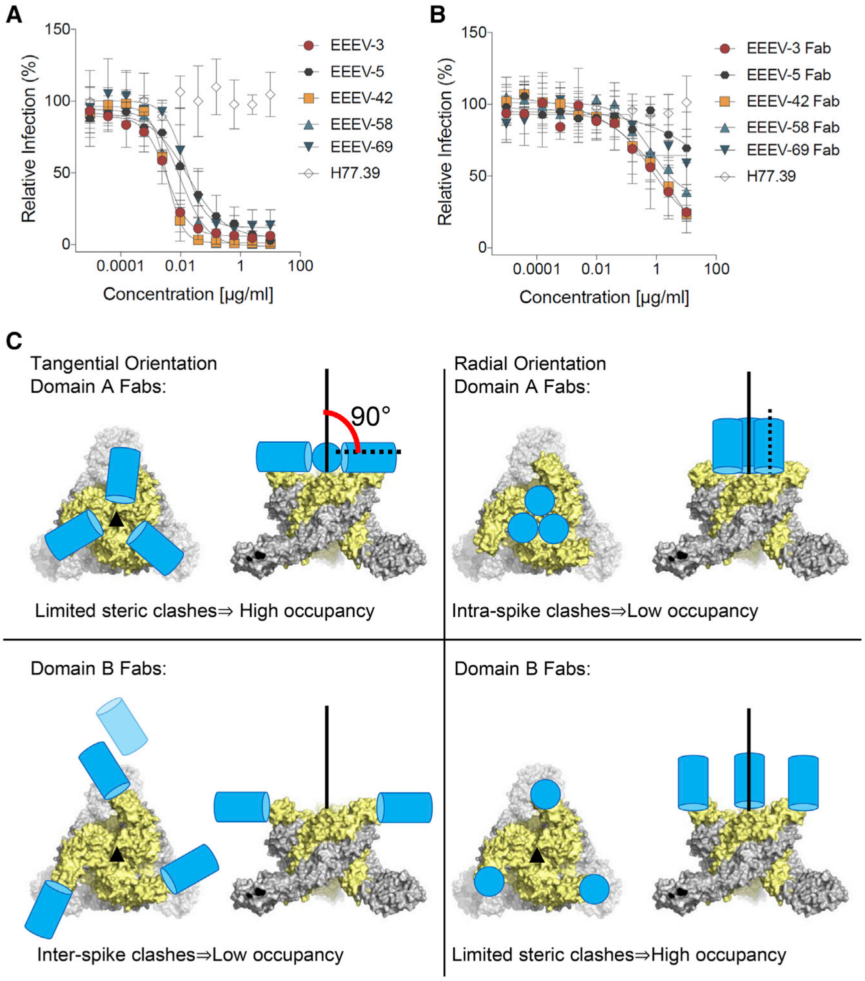

Figure 7. Neutralization of EEEV Infection

(A and B) Focus reduction neutralization assay. (A) Bivalent mouse IgG mAbs inhibit infection efficiently unlike (B) monovalent Fabs (hepatitis C virus-specific mAb H77.39, negative control). The neutralization experiments were performed twice, each time in duplicate, and the curves report mean values and SDs.

(C) Steric restrictions on Fab binding to alphaviruses. E1-E2 spike, gray-yellow; Fab, blue cylinder; Fab quasi-2-fold axis, broken line. Tangential binding does not cause significant clashes between domain A Fabs bound to a spike (upper left) unlike radial binding (upper right). In contrast, tangential binding of domain B Fabs may encounter clashes with Fabs bound to neighboring spikes and even with neighboring spikes (lower left), unlike radial binding (lower right).

(Figures S7E and S7F) (Haag et al., 2002; Harrison, 2008; Lescar et al., 2001; Uchime et al., 2013; Wahlberg et al., 1992), with both proteins having an acidic character (Tables S4 and S7).

Structure of the Capsid Protein

The two-layered structure of the EEEV capsid core described here suggests a mechanism for capsid shell disassembly in the cytosol. Host membrane-to-E1 fusion will decrease the curvature of the E1-E2 layer. As only a short E2 endodomain interacts with the capsid shell, this may initiate “peeling off” of the E1-E2 layer from the NC. In the absence of stabilizing contacts from the envelope protein layer, disassembly of the NC may be initiated at the 3-fold axes that lack stabilizing interactions. Indeed, NCs have been shown to form holes and undergo expansion upon release from alphaviruses, implying that the released NCs are less compact in monomer-monomer associations (Paredes et al., 2003). Moreover, the EEEV capsid-capsid interface is enriched in complementary charged residues, which would be sensitive to pH changes and supports a disassembly mechanism based on acidification (Wengler and Wengler, 2002). Based on the present structural analysis, it could be speculated that the released alphavirus capsid may function as scaffolds that place the viral RNA genome, which acts similarly to a host cell mRNA molecule, next to a ribosome to initiate viral protein synthesis. However, evidence that demonstrates increased efficiency of translation due to ribosome binding next to the mRNA site is currently not available.

The presence of one RNA genome per alphavirus particle and 240 capsid proteins causes heterogeneity in RNA-capsid interactions. However, the icosahedrally averaged EEEV cryo-EM map shows an ordered main-chain for the capsid genome binding residues Lys82-Lys112, implying the occurrence of repetitive RNA-capsid interactions across 60 icosahedral asymmetric units. The occurrence of short sequence repeats in alphavirus genomes (Alam et al., 2014) suggests the possibility of structurally similar RNA-capsid interactions in each icosahedral asymmetric unit.

Non-genomic single-stranded nucleic acids have been reported to initiate NC assembly (Tellinghuisen et al., 1999). However, in the absence of high-resolution reconstructions of artificially assembled NCs, it is currently not possible to evaluate how strictly specific RNA-capsid interactions are required for the assembly of icosahedral NCs.

Structural Mechanism of EEEV Inhibition by Neutralizing mAbs

The structural analysis of Fab binding described here has general applications for icosahedral viruses, which demonstrate clustering of epitopes close to symmetry axes. This inhibits Fab or mAb binding to symmetry-related epitopes.

In this analysis, only the mAbs and not the Fab fragments achieve significant neutralization unlike the common observation that both bivalent mAbs and monovalent Fabs achieve neutralization (Edeling et al., 2014; Fibriansah et al., 2015). We speculate that cross-linking of E2 domains by bivalent mAbs augments neutralizing activity. An alternative explanation for the poor

neutralization potency of Fabs is the loss of avidity due to mAb digestion into a monovalent Fab, although Fabs were able to bind to the E2 protein.

Fab-complexed structures provide insights into steric factors that influence mAb interactions, although saturation binding of the viral envelope proteins is not required for neutralization (Pierson et al., 2007) and Fab occupancies may not directly correlate with mAb potency. Icosahedral symmetry imposes steric constraints that are different for Fabs binding to domain A compared to domain B. In extreme cases, the Fab quasi-2-fold axis can be parallel (''radial'') or perpendicular (''tangential'') to the 3-fold axis of the trimeric spikes (Figure 7C). A Fab bound to domain A in a radial orientation will clash with Fabs bound to the other two symmetry-related positions on the same trimeric spike lowering average Fab occupancy (Figure 7C). This is consistent with the low occupancy of the approximately radially binding EEEV-5 Fab and with the higher occupancy of the more tangentially binding EEEV-58 Fab. Low-occupancy radial binding of Fab or mAb to domain A might result in incomplete blocking of receptor binding sites. In contrast to domain A Fabs, a tangential binding orientation of a domain B Fab will result in clashes with Fabs bound to neighboring spikes and also with E1-E2 dimers from neighboring spikes, thereby lowering the average Fab occupancy (Figure 7C). This will result in incomplete inhibition of pH-triggered conformational changes of domain B.

Here, it seems likely that mAbs whose Fabs bind to E2 domain A cause intraspike cross-linking due to the presence of three symmetry-related copies of domain A near the 3-fold axis of the trimeric spikes. In contrast, mAbs whose Fabs bind to domain B may favor interspike cross-linking as domain B is located at the tip of the E1-E2 dimer. Differences in the occupancies of Fabs that have similar orientations can be attributed to variations in the paratope sequence and Fab affinity for the epitope. This may be the case for EEEV-42 and EEEV-58 Fabs that have similar orientations but different occupancies.

In summary, this investigation has provided mechanistic insights into EEEV entry into host cells and the inhibition of EEEV infections by neutralizing mAbs.

STAR★METHODS

Detailed methods are provided in the online version of this paper and include the following:

- KEY RESOURCES TABLE
- CONTACT FOR REAGENT AND RESOURCE SHARING
- EXPERIMENTAL MODEL AND SUBJECT DETAILS
- METHOD DETAILS
  - Purification of SINV-EEEV chimeric particles from mammalian cells
  - Purification of SINV-EEEV chimeric particles from mosquito cells
  - CryoEM data collection
  - CryoEM data processing
  - CryoEM model building
  - Interpretation of EEEV-Fab complex structures
  - Calculation of isoelectric points
  - Prediction of glycosylation sites
  - Quantitative glycan analysis by hydrophilic interaction chromatography-ultra performance liquid chromatography (HILIC-UPLC)
  - Flow cytometry analysis
  - mAb production
  - Focus reduction neutralization assay
- QUANTIFICATION AND STATISTICAL ANALYSIS
- DATA AND SOFTWARE AVAILABILITY

SUPPLEMENTAL INFORMATION

Supplemental Information includes seven figures and seven tables and can be found with this article online at https://doi.org/10.1016/j.celrep.2018.11.067.

ACKNOWLEDGMENTS

We thank Yingyuan Sun and Valorie D. Bowman (Purdue University) for technical help in cryo-EM data collection and Scott Weaver (University of Texas Medical Branch at Galveston) for advice on EEEV purification. Cryo-EM data were collected at the Purdue University Cryo-EM Facility. This work was supported by NIH Grant Awards NIH/NIAID/R01AI095366 (M.G.R.), NIH/NIAID/2R01AI095436 (W.B.K.), and NIH/NIAID/R21AI117331-01 (W.B.K.); Defense Threat Reduction Agency Awards DTRA HDTRA 1-15-1-0047 (W.B.K.) and DTRA HDTRA 1-15-1-0013 (M.S.D.); and Bill and Melinda Gates Foundation through Collaboration for AIDS Vaccine Discovery Grant OPP1115782 (M.C.).

AUTHOR CONTRIBUTIONS

M.G.R., M.S.D., S.S.H., and W.B.K. conceived the project. A.S.K. purified monoclonal antibodies and performed neutralization assays. S.S.H. purified Fab fragments from antibodies. C.S. generated SINV-EEEV chimera. C.-L.C., G.B., and S.S.H. purified EEEV virions. S.S.H. froze EEEV virions and EEEV-Fab complexes on cryo-EM grids. S.S.H. and T.K. collected cryo-EM data and performed cryo-EM reconstruction and model building. Y.W. and M.C. performed glycan analysis. C.S. and W.B.K. performed FACS analysis. All authors contributed to data analysis and discussions. A.S.K., M.C., M.G.R., M.S.D., S.S.H., W.B.K., and Y.W. wrote the manuscript.

DECLARATION OF INTERESTS

M.S.D. is a consultant for Inbios and on the Scientific Advisory Board of Moderna.

Received: August 13, 2018
Revised: October 1, 2018
Accepted: November 15, 2018
Published: December 11, 2018

REFERENCES

Adams, P.D., Afonine, P.V., Bunkóczi, G., Chen, V.B., Davis, I.W., Echols, N., Headd, J.J., Hung, L.W., Kapral, G.J., Grosse-Kunstleve, R.W., et al. (2010). PHENIX: A comprehensive Python-based system for macromolecular structure solution. Acta Crystallogr. D Biol. Crystallogr. *66*, 213–221.

Alam, C.M., Singh, A.K., Sharfuddin, C., and Ali, S. (2014). In- silico exploration of thirty alphavirus genomes for analysis of the simple sequence repeats. Meta Gene *2*, 694–705.

Alford, R.F., Leaver-Fay, A., Jeliazkov, J.R., O'Meara, M.J., DiMaio, F.P., Park, H., Shapovalov, M.V., Renfrew, P.D., Mulligan, V.K., Kappel, K., et al. (2017). The Rosetta all-atom energy function for macromolecular modeling and design. J. Chem. Theory Comput. *13*, 3031–3048.

Armstrong, P.M., and Andreadis, T.G. (2013). Eastern equine encephalitis virus—old enemy, new threat. N. Engl. J. Med. *368*, 1670–1673.

Barrera, F.N., Weerakkody, D., Anderson, M., Andreev, O.A., Reshetnyak, Y.K., and Engelman, D.M. (2011). Roles of carboxyl groups in the transmembrane insertion of peptides. J. Mol. Biol. *413*, 359–371.

Carragher, B., Kisseberth, N., Kriegman, D., Milligan, R.A., Potter, C.S., Pulokas, J., and Reilein, A. (2000). Leginon: An automated system for acquisition of images from vitreous ice specimens. J. Struct. Biol. *132*, 33–45.

Carrera, J.P., Forrester, N., Wang, E., Vittor, A.Y., Haddow, A.D., López-Vergès, S., Abadía, I., Castaño, E., Sosa, N., Báez, C., et al. (2013). Eastern equine encephalitis in Latin America. N. Engl. J. Med. *369*, 732–744.

Chatterjee, P.K., Eng, C.H., and Kielian, M. (2002). Novel mutations that control the sphingolipid and cholesterol dependence of the Semliki Forest virus fusion protein. J. Virol. *76*, 12712–12722.

Chen, V.B., Arendall, W.B., 3rd, Headd, J.J., Keedy, D.A., Immormino, R.M., Kapral, G.J., Murray, L.W., Richardson, J.S., and Richardson, D.C. (2010). MolProbity: all-atom structure validation for macromolecular crystallography. Acta Crystallogr. D Biol. Crystallogr. *66*, 12–21.

Chen, J., Rowe, C.L., Jardetzky, T.S., and Longnecker, R. (2012). The KGD motif of Epstein-Barr virus gH/gL is bifunctional, orchestrating infection of B cells and epithelial cells. mBio *3*, e00290-11.

Choi, H.K., Tong, L., Minor, W., Dumas, P., Boege, U., Rossmann, M.G., and Wengler, G. (1991). Structure of Sindbis virus core protein reveals a chymotrypsin-like serine proteinase and the organization of the virion. Nature *354*, 37–43.

Crispin, M., Harvey, D.J., Bitto, D., Bonomelli, C., Edgeworth, M., Scrivens, J.H., Huiskonen, J.T., and Bowden, T.A. (2014). Structural plasticity of the Semliki Forest virus glycome upon interspecies transmission. J. Proteome Res. *13*, 1702–1712.

Edeling, M.A., Austin, S.K., Shrestha, B., Dowd, K.A., Mukherjee, S., Nelson, C.A., Johnson, S., Mabila, M.N., Christian, E.A., Rucker, J., et al. (2014). Potent dengue virus neutralization by a therapeutic antibody with low monovalent affinity requires bivalent engagement. PLoS Pathog. *10*, e1004072.

Emsley, P., and Cowtan, K. (2004). Coot: Model-building tools for molecular graphics. Acta Crystallogr. D Biol. Crystallogr. *60*, 2126–2132.

Fibriansah, G., Tan, J.L., Smith, S.A., de Alwis, R., Ng, T.S., Kostyuchenko, V.A., Jadi, R.S., Kukkaro, P., de Silva, A.M., Crowe, J.E., and Lok, S.M. (2015). A highly potent human antibody neutralizes dengue virus serotype 3 by binding across three surface proteins. Nat. Commun. *6*, 6341.

Fuster, M.M., and Wang, L. (2010). Endothelial heparan sulfate in angiogenesis. Prog. Mol. Biol. Transl. Sci. *93*, 179–212.

Gardner, C.L., Ebel, G.D., Ryman, K.D., and Klimstra, W.B. (2011). Heparan sulfate binding by natural eastern equine encephalitis viruses promotes neurovirulence. Proc. Natl. Acad. Sci. USA *108*, 16026–16031.

Gardner, C.L., Choi-Nurvitadhi, J., Sun, C., Bayer, A., Hritz, J., Ryman, K.D., and Klimstra, W.B. (2013). Natural variation in the heparan sulfate binding domain of the eastern equine encephalitis virus E2 glycoprotein alters interactions with cell surfaces and virulence in mice. J. Virol. *87*, 8582–8590.

Gasteiger, E., Gattiker, A., Hoogland, C., Ivanyi, I., Appel, R.D., and Bairoch, A. (2003). ExPASy: The proteomics server for in-depth protein knowledge and analysis. Nucleic Acids Res. *31*, 3784–3788.

Gavel, Y., and von Heijne, G. (1990). Sequence differences between glycosylated and non-glycosylated Asn-X-Thr/Ser acceptor sites: Implications for protein engineering. Protein Eng. *3*, 433–442.

Gibbons, D.L., Erk, I., Reilly, B., Navaza, J., Kielian, M., Rey, F.A., and Lepault, J. (2003). Visualization of the target-membrane-inserted fusion protein of Semliki Forest virus by combined electron microscopy and crystallography. Cell *114*, 573–583.

Gibbons, D.L., Vaney, M.C., Roussel, A., Vigouroux, A., Reilly, B., Lepault, J., Kielian, M., and Rey, F.A. (2004). Conformational change and protein-protein interactions of the fusion protein of Semliki Forest virus. Nature *427*, 320–325.

Gouet, P., Robert, X., and Courcelle, E. (2003). ESPript/ENDscript: Extracting and rendering sequence and 3D information from atomic structures of proteins. Nucleic Acids Res. *31*, 3320–3323.

Guirakhoo, F., Bolin, R.A., and Roehrig, J.T. (1992). The Murray Valley encephalitis virus prM protein confers acid resistance to virus particles and alters the expression of epitopes within the R2 domain of E glycoprotein. Virology *191*, 921–931.

Guo, F., and Jiang, W. (2014). Single particle cryo-electron microscopy and 3-D reconstruction of viruses. Methods Mol. Biol. *1117*, 401–443.

Haag, L., Garoff, H., Xing, L., Hammar, L., Kan, S.T., and Cheng, R.H. (2002). Acid-induced movements in the glycoprotein shell of an alphavirus turn the spikes into membrane fusion mode. EMBO J. *21*, 4402–4410.

Harrison, S.C. (2008). Viral membrane fusion. Nat. Struct. Mol. Biol. *15*, 690–698.

Hirschberg, C.B., and Robbins, P.W. (1974). The glycolipids and phospholipids of Sindbis virus and their relation to the lipids of the host cell plasma membrane. Virology *61*, 602–608.

Jemielity, S., Wang, J.J., Chan, Y.K., Ahmed, A.A., Li, W., Monahan, S., Bu, X., Farzan, M., Freeman, G.J., Umetsu, D.T., et al. (2013). TIM-family proteins promote infection of multiple enveloped viruses through virion-associated phosphatidylserine. PLoS Pathog. *9*, e1003232.

Jose, J., Snyder, J.E., and Kuhn, R.J. (2009). A structural and functional perspective of alphavirus replication and assembly. Future Microbiol. *4*, 837–856.

Kim, A.S., Austin, S.K., Gardner, C.L., Zuiani, A., Reed, D.S., Williamson, L.E., Crowe, J.J.E., Slifka, M.K., Fremont, D.H., Klimstra, W.B., et al. (2018). Protective antibodies against eastern equine encephalitis virus bind to epitopes 2 in domains A and B of the E2 glycoprotein. Nat. Microbiol., Published online November 19, 2018. https://doi.org/10.1038/s41564-018-0286-4.

Klimjack, M.R., Jeffrey, S., and Kielian, M. (1994). Membrane and protein interactions of a soluble form of the Semliki Forest virus fusion protein. J. Virol. *68*, 6940–6946.

Klimstra, W.B., Nangle, E.M., Smith, M.S., Yurochko, A.D., and Ryman, K.D. (2003). DC-SIGN and L-SIGN can act as attachment receptors for alphaviruses and distinguish between mosquito cell- and mammalian cell-derived viruses. J. Virol. *77*, 12022–12032.

Kostyuchenko, V.A., Jakana, J., Liu, X., Haddow, A.D., Aung, M., Weaver, S.C., Chiu, W., and Lok, S.M. (2011). The structure of barmah forest virus as revealed by cryo-electron microscopy at a 6-angstrom resolution has detailed transmembrane protein architecture and interactions. J. Virol. *85*, 9327–9333.

Kucukelbir, A., Sigworth, F.J., and Tagare, H.D. (2014). Quantifying the local resolution of cryo-EM density maps. Nat. Methods *11*, 63–65.

Kwon, D.S., Gregorio, G., Bitton, N., Hendrickson, W.A., and Littman, D.R. (2002). DC-SIGN-mediated internalization of HIV is required for trans-enhancement of T cell infection. Immunity *16*, 135–144.

La Linn, M., Eble, J.A., Lübken, C., Slade, R.W., Heino, J., Davies, J., and Suhrbier, A. (2005). An arthritogenic alphavirus uses the $\alpha1\beta1$ integrin collagen receptor. Virology *336*, 229–239.

Laine, R., Kettunen, M.L., Gahmberg, C.G., Kääriäinen, L., and Renkonen, O. (1972). Fatty chains of different lipid classes of Semliki Forest virus and host cell membranes. J. Virol. *10*, 433–438.

Lander, G.C., Stagg, S.M., Voss, N.R., Cheng, A., Fellmann, D., Pulokas, J., Yoshioka, C., Irving, C., Mulder, A., Lau, P.W., et al. (2009). Appion: An integrated, database-driven pipeline to facilitate EM image processing. J. Struct. Biol. *166*, 95–102.

Lescar, J., Roussel, A., Wien, M.W., Navaza, J., Fuller, S.D., Wengler, G., Wengler, G., and Rey, F.A. (2001). The fusion glycoprotein shell of Semliki Forest virus: an icosahedral assembly primed for fusogenic activation at endosomal pH. Cell *105*, 137–148.

Leventis, P.A., and Grinstein, S. (2010). The distribution and function of phosphatidylserine in cellular membranes. Annu. Rev. Biophys. *39*, 407–427.

Levine, B., Huang, Q., Isaacs, J.T., Reed, J.C., Griffin, D.E., and Hardwick, J.M. (1993). Conversion of lytic to persistent alphavirus infection by the bcl-2 cellular oncogene. Nature *361*, 739–742.

Li, L., Jose, J., Xiang, Y., Kuhn, R.J., and Rossmann, M.G. (2010). Structural changes of envelope proteins during alphavirus fusion. Nature *468*, 705–708.

Long, F., Fong, R.H., Austin, S.K., Chen, Z., Klose, T., Fokine, A., Liu, Y., Porta, J., Sapparapu, G., Akahata, W., et al. (2015). Cryo-EM structures elucidate neutralizing mechanisms of anti-chikungunya human monoclonal antibodies with therapeutic activity. Proc. Natl. Acad. Sci. USA *112*, 13898–13903.

Lozach, P.Y., Burleigh, L., Staropoli, I., and Amara, A. (2007). The C type lectins DC-SIGN and L-SIGN: Receptors for viral glycoproteins. Methods Mol. Biol. *379*, 51–68.

Mancini, E.J., Clarke, M., Gowen, B.E., Rutten, T., and Fuller, S.D. (2000). Cryo-electron microscopy reveals the functional organization of an enveloped virus, Semliki Forest virus. Mol. Cell *5*, 255–266.

Mason, P.W., Rieder, E., and Baxt, B. (1994). RGD sequence of foot-and-mouth disease virus is essential for infecting cells via the natural receptor but can be bypassed by an antibody-dependent enhancement pathway. Proc. Natl. Acad. Sci. USA *91*, 1932–1936.

McKnight, K.L., Simpson, D.A., Lin, S.C., Knott, T.A., Polo, J.M., Pence, D.F., Johannsen, D.B., Heidner, H.W., Davis, N.L., and Johnston, R.E. (1996). Deduced consensus sequence of Sindbis virus strain AR339: Mutations contained in laboratory strains which affect cell culture and in vivo phenotypes. J. Virol. *70*, 1981–1989.

Mitchell, D.A., Fadden, A.J., and Drickamer, K. (2001). A novel mechanism of carbohydrate recognition by the C-type lectins DC-SIGN and DC-SIGNR. Subunit organization and binding to multivalent ligands. J. Biol. Chem. *276*, 28939–28945.

Moller-Tank, S., Kondratowicz, A.S., Davey, R.A., Rennert, P.D., and Maury, W. (2013). Role of the phosphatidylserine receptor TIM-1 in enveloped-virus entry. J. Virol. *87*, 8327–8341.

Mukhopadhyay, S., Zhang, W., Gabler, S., Chipman, P.R., Strauss, E.G., Strauss, J.H., Baker, T.S., Kuhn, R.J., and Rossmann, M.G. (2006). Mapping the structure and function of the E1 and E2 glycoproteins in alphaviruses. Structure *14*, 63–73.

Owen, K.E., and Kuhn, R.J. (1996). Identification of a region in the Sindbis virus nucleocapsid protein that is involved in specificity of RNA encapsidation. J. Virol. *70*, 2757–2763.

Paredes, A., Alwell-Warda, K., Weaver, S.C., Chiu, W., and Watowich, S.J. (2003). Structure of isolated nucleocapsids from venezuelan equine encephalitis virus and implications for assembly and disassembly of enveloped virus. J. Virol. *77*, 659–664.

Pettersen, E.F., Goddard, T.D., Huang, C.C., Couch, G.S., Greenblatt, D.M., Meng, E.C., and Ferrin, T.E. (2004). UCSF Chimera—a visualization system for exploratory research and analysis. J. Comput. Chem. *25*, 1605–1612.

Pierson, T.C., Xu, Q., Nelson, S., Oliphant, T., Nybakken, G.E., Fremont, D.H., and Diamond, M.S. (2007). The stoichiometry of antibody-mediated neutralization and enhancement of West Nile virus infection. Cell Host Microbe *1*, 135–145.

Porta, J., Jose, J., Roehrig, J.T., Blair, C.D., Kuhn, R.J., and Rossmann, M.G. (2014). Locking and blocking the viral landscape of an alphavirus with neutralizing antibodies. J. Virol. *88*, 9616–9623.

Pritchard, L.K., Spencer, D.I., Royle, L., Bonomelli, C., Seabright, G.E., Behrens, A.J., Kulp, D.W., Menis, S., Krumm, S.A., Dunlop, D.C., et al. (2015). Glycan clustering stabilizes the mannose patch of HIV-1 and preserves vulnerability to broadly neutralizing antibodies. Nat. Commun. *6*, 7479.

Rohou, A., and Grigorieff, N. (2015). CTFFIND4: Fast and accurate defocus estimation from electron micrographs. J. Struct. Biol. *192*, 216–221.

Rosenthal, P.B., and Henderson, R. (2003). Optimal determination of particle orientation, absolute hand, and contrast loss in single-particle electron cryomicroscopy. J. Mol. Biol. *333*, 721–745.

Rossmann, M.G., Bernal, R., and Pletnev, S.V. (2001). Combining electron microscopic with x-ray crystallographic structures. J. Struct. Biol. *136*, 190–200.

Roy, C.J., Reed, D.S., Wilhelmsen, C.L., Hartings, J., Norris, S., and Steele, K.E. (2009). Pathogenesis of aerosolized eastern equine encephalitis virus infection in guinea pigs. Virol. J. *6*, 170.

Ruoslahti, E. (1996). RGD and other recognition sequences for integrins. Annu. Rev. Cell Dev. Biol. *12*, 697–715.

Sabo, M.C., Luca, V.C., Prentoe, J., Hopcraft, S.E., Blight, K.J., Yi, M., Lemon, S.M., Ball, J.K., Bukh, J., Evans, M.J., et al. (2011). Neutralizing monoclonal antibodies against hepatitis C virus E2 protein bind discontinuous epitopes and inhibit infection at a postattachment step. J. Virol. *85*, 7005–7019.

Scheres, S.H. (2012). RELION: Implementation of a Bayesian approach to cryo-EM structure determination. J. Struct. Biol. *180*, 519–530.

Scheres, S.H., and Chen, S. (2012). Prevention of overfitting in cryo-EM structure determination. Nat. Methods *9*, 853–854.

Schwartz, O., and Albert, M.L. (2010). Biology and pathogenesis of chikungunya virus. Nat. Rev. Microbiol. *8*, 491–500.

Sherman, M.B., and Weaver, S.C. (2010). Structure of the recombinant alphavirus western equine encephalitis virus revealed by cryoelectron microscopy. J. Virol. *84*, 9775–9782.

Sievers, F., Wilm, A., Dineen, D., Gibson, T.J., Karplus, K., Li, W., Lopez, R., McWilliam, H., Remmert, M., Söding, J., et al. (2011). Fast, scalable generation of high-quality protein multiple sequence alignments using Clustal Omega. Mol. Syst. Biol. *7*, 539.

Silverman, M.A., Misasi, J., Smole, S., Feldman, H.A., Cohen, A.B., Santagata, S., McManus, M., and Ahmed, A.A. (2013). Eastern equine encephalitis in children, Massachusetts and New Hampshire, USA, 1970–2010. Emerg. Infect. Dis. *19*, 194–201, quiz 352.

Smith, T.J., Cheng, R.H., Olson, N.H., Peterson, P., Chase, E., Kuhn, R.J., and Baker, T.S. (1995). Putative receptor binding sites on alphaviruses as visualized by cryoelectron microscopy. Proc. Natl. Acad. Sci. USA *92*, 10648–10652.

Strauss, J.H., and Strauss, E.G. (1994). The alphaviruses: gene expression, replication, and evolution. Microbiol. Rev. *58*, 491–562.

Sun, S., Xiang, Y., Akahata, W., Holdaway, H., Pal, P., Zhang, X., Diamond, M.S., Nabel, G.J., and Rossmann, M.G. (2013). Structural analyses at pseudo atomic resolution of chikungunya virus and antibodies show mechanisms of neutralization. eLife *2*, e00435.

Tang, J., Jose, J., Chipman, P., Zhang, W., Kuhn, R.J., and Baker, T.S. (2011). Molecular links between the E2 envelope glycoprotein and nucleocapsid core in Sindbis virus. J. Mol. Biol. *414*, 442–459.

Tellinghuisen, T.L., Hamburger, A.E., Fisher, B.R., Ostendorp, R., and Kuhn, R.J. (1999). In vitro assembly of alphavirus cores by using nucleocapsid protein expressed in *Escherichia coli*. J. Virol. *73*, 5309–5319.

Trobaugh, D.W., Gardner, C.L., Sun, C., Haddow, A.D., Wang, E., Chapnik, E., Mildner, A., Weaver, S.C., Ryman, K.D., and Klimstra, W.B. (2014). RNA viruses can hijack vertebrate microRNAs to suppress innate immunity. Nature *506*, 245–248.

Uchime, O., Fields, W., and Kielian, M. (2013). The role of E3 in pH protection during alphavirus assembly and exit. J. Virol. *87*, 10255–10262.

Uversky, V.N. (2013). A decade and a half of protein intrinsic disorder: Biology still waits for physics. Protein Sci. *22*, 693–724.

van Duijl-Richter, M.K., Hoornweg, T.E., Rodenhuis-Zybert, I.A., and Smit, J.M. (2015). Early events in chikungunya virus infection—from virus cell binding to membrane fusion. Viruses *7*, 3647–3674.

Vashishtha, M., Phalen, T., Marquardt, M.T., Ryu, J.S., Ng, A.C., and Kielian, M. (1998). A single point mutation controls the cholesterol dependence of Semliki Forest virus entry and exit. J. Cell Biol. *140*, 91–99.

Villari, P., Spielman, A., Komar, N., McDowell, M., and Timperi, R.J. (1995). The economic burden imposed by a residual case of eastern encephalitis. Am. J. Trop. Med. Hyg. *52*, 8–13.

Voss, J.E., Vaney, M.C., Duquerroy, S., Vonrhein, C., Girard-Blanc, C., Crublet, E., Thompson, A., Bricogne, G., and Rey, F.A. (2010). Glycoprotein organization of chikungunya virus particles revealed by X-ray crystallography. Nature *468*, 709–712.

Wahlberg, J.M., Bron, R., Wilschut, J., and Garoff, H. (1992). Membrane fusion of Semliki Forest virus involves homotrimers of the fusion protein. J. Virol. *66*, 7309–7318.

Wengler, G., and Wengler, G. (2002). In vitro analysis of factors involved in the disassembly of Sindbis virus cores by 60S ribosomal subunits identifies a possible role of low pH. J. Gen. Virol. *83*, 2417–2426.

Wengler, G., Würkner, D., and Wengler, G. (1992). Identification of a sequence element in the alphavirus core protein which mediates interaction of cores with ribosomes and the disassembly of cores. Virology *191*, 880–888.

Xiao, C., and Rossmann, M.G. (2007). Interpretation of electron density with stereographic roadmap projections. J. Struct. Biol. *158*, 182–187.

Yang, J., Yan, R., Roy, A., Xu, D., Poisson, J., and Zhang, Y. (2015). The I-TASSER Suite: Protein structure and function prediction. Nat. Methods *12*, 7–8.

Yuan, S., Cao, L., Ling, H., Dang, M., Sun, Y., Zhang, X., Chen, Y., Zhang, L., Su, D., Wang, X., and Rao, Z. (2015). TIM-1 acts a dual-attachment receptor for Ebolavirus by interacting directly with viral GP and the PS on the viral envelope. Protein Cell *6*, 814–824.

Zhang, W., Mukhopadhyay, S., Pletnev, S.V., Baker, T.S., Kuhn, R.J., and Rossmann, M.G. (2002). Placement of the structural proteins in Sindbis virus. J. Virol. *76*, 11645–11658.

Zhang, W., Heil, M., Kuhn, R.J., and Baker, T.S. (2005). Heparin binding sites on Ross River virus revealed by electron cryo-microscopy. Virology *332*, 511–518.

Zhang, R., Hryc, C.F., Cong, Y., Liu, X., Jakana, J., Gorchakov, R., Baker, M.L., Weaver, S.C., and Chiu, W. (2011). 4.4 Å cryo-EM structure of an enveloped alphavirus Venezuelan equine encephalitis virus. EMBO J. *30*, 3854–3863.

Zhang, R., Kim, A.S., Fox, J.M., Nair, S., Basore, K., Klimstra, W.B., Rimkunas, R., Fong, R.H., Lin, H., Poddar, S., et al. (2018). Mxra8 is a receptor for multiple arthritogenic alphaviruses. Nature *557*, 570–574.

Zheng, S.Q., Palovcak, E., Armache, J.P., Verba, K.A., Cheng, Y., and Agard, D.A. (2017). MotionCor2: Anisotropic correction of beam-induced motion for improved cryo-electron microscopy. Nat. Methods *14*, 331–332.

STAR★METHODS

KEY RESOURCES TABLE

REAGENT or RESOURCE	SOURCE	IDENTIFIER
Antibodies		
EEEV-3	Diamond Lab	Kim et al., 2018
EEEV-5	Diamond Lab	Kim et al., 2018
EEEV-42	Diamond Lab	Kim et al., 2018
EEEV-58	Diamond Lab	Kim et al., 2018
EEEV-69	Diamond Lab	Kim et al., 2018
H77.39	Diamond Lab	Sabo et al., 2011
Peroxidase AffiniPure Goat Anti-Mouse IgG (H+L)	Jackson ImmunoResearch	115-032-062
Bacterial and Virus Strains		
SINV(TR339)-EEEV(FL93-939) chimera	Kim et al., 2018	N/A
SINV-TR339	Klimstra et al., 2003	N/A
Chemicals, Peptides, and Recombinant Proteins		
Acetonitrile	Fisher Scientific	Cat# 10489553
Procainamide hydrochloride	Abcam	Cat# ab120955
Dithiothreitol	Sigma-Aldrich	Cat# 43819
Ammonium formate buffer	Waters	Cat# 186007081
Sodium cyanoborohydride	Sigma-Aldrich	Cat# 156159
DMSO	Sigma-Aldrich	Cat# D2438
Acetic acid	Fisher Scientific	Cat# 10384970
PNGase F	New England BioLabs	Cat# P0705S
Endoglycosidase H	New England BioLabs	Cat# P0702S
PNGase A	New England BioLabs	Cat# P0707S
Critical Commercial Assays		
Pierce Fab Preparation Kit	Thermo Scientific	Cat# 44985
Deposited Data		
CryoEM map of native SINV-EEEV chimera (4.4Å)	This paper	EMD-9280
CryoEM map of native SINV-EEEV chimera showing N-terminal chain (4.8Å)	This paper	EMD-9281
CryoEM map of SINV-EEEV chimera with Fab of EEEV-3	This paper	EMD-9274
CryoEM map of SINV-EEEV chimera with Fab of EEEV-5	This paper	EMD-9275
CryoEM map of SINV-EEEV chimera with Fab of EEEV-42	This paper	EMD-9249
CryoEM map of SINV-EEEV chimera with Fab of EEEV-58	This paper	EMD-9278
CryoEM map of SINV-EEEV chimera with Fab of EEEV-69	This paper	EMD-9279
Coordinates of E1-E2-capsid protein for native SINV-EEEV cryoEM map	This paper	PDB ID 6MX4
Coordinates of capsid protein for native SINV-EEEV cryoEM map	This paper	PDB ID 6MX7
Coordinates of E1-E2 ecto-domains and fitted Fab homolog in SINV-EEEV: EEEV-3 cryoEM map	This paper	PDB ID 6MW9
Coordinates of E1-E2 ecto-domains and fitted Fab homolog in SINV-EEEV: EEEV-5 cryoEM map	This paper	PDB ID 6MWC
Coordinates of E1-E2 ecto-domains and fitted Fab homolog in SINV-EEEV: EEEV-42 cryoEM map	This paper	PDB ID 6MUI
Coordinates of E1-E2 ecto-domains and fitted Fab homolog in SINV-EEEV: EEEV-58 cryoEM map	This paper	PDB ID 6MWV
Coordinates of E1-E2 ecto-domains and fitted Fab homolog in SINV-EEEV: EEEV-69 cryoEM map	This paper	PDB ID 6MWX

(*Continued on next page*)

Continued		
REAGENT or RESOURCE	SOURCE	IDENTIFIER
Experimental Models: Cell Lines		
African green monkey kidney (Vero) cell	WHO Reference Cell Bank	WHO Vero cells
Baby Hamster Kidney (BHK)-15 cells	Kuhn Lab (Purdue University)	N/A
Aedes albopictus C6/36	ATCC	Cat#CRL-1660
Raji B cells expressing DC-SIGN	Kwon et al., 2002	N/A
Raji B cells expressing L-SIGN	Kwon et al., 2002	N/A
Raji B cells	Kwon et al., 2002	N/A
Software and Algorithms		
Prism	GraphPad Software	Prism Version 7.0d
Empower 3.0	Waters	N/A
NetNGlyc 1.0	http://www.cbs.dtu.dk/services/NetNGlyc/	N/A
Leginon	Carragher et al., 2000	N/A
Motioncor2	Zheng et al., 2017	N/A
CTFFIND4	Rohou and Grigorieff, 2015	N/A
Appion	Lander et al., 2009	N/A
Relion	Scheres, 2012	N/A
jspr	Guo and Jiang, 2014	N/A
ResMap	Kucukelbir et al., 2014	N/A
Phenix Real Space Refine	Adams et al., 2010	N/A
Rosetta	Alford et al., 2017	N/A
MolProbity	Chen et al., 2010	N/A
COOT	Emsley and Cowtan, 2004	N/A
PyMol	https://pymol.org/2/	N/A
Chimera	Pettersen et al., 2004	N/A
RIVEM	Xiao and Rossmann, 2007	N/A
EMfit	Rossmann et al., 2001	N/A
I-TASSER	Yang et al., 2015	N/A
Clustal Omega	Sievers et al., 2011	N/A
ESpript	Gouet et al., 2003	N/A
ExPasy	Gasteiger et al., 2003	N/A
Other		
Spe-ed Amide 2 cartridges	Applied Separations	Cat# 4821
Glycan BEH Amide column (2.1 mm x 100 mm, 1.7 μM)	Waters	Cat# 186004741
PVDF protein-binding membrane	Millipore	Cat# MAIPS4510
Ultrathin continuous carbon grids	Ted Pella	Cat# 01824

CONTACT FOR REAGENT AND RESOURCE SHARING

Further information and requests for resources and reagents should be directed to and will be fulfilled by the Lead Contact Michael G. Rossmann (mr@purdue.edu). An approved Material Transfer Agreement (MTA) may be required for resource sharing.

EXPERIMENTAL MODEL AND SUBJECT DETAILS

Baby hamster kidney (BHK)-15 cells were obtained from the laboratory of Richard J. Kuhn (Purdue University). Vero cells were obtained from the WHO Reference Cell Bank. C6/36 cells were obtained from ATCC. Raji B cells including those engineered to express DC-SIGN and L-SIGN were obtained from the laboratory of Dan Littman, New York University School of Medicine. The construction of the SINV (TR339 strain)-EEEV (FL93-939 strain) chimera is reported elsewhere (Kim et al., 2018). All virus-infected cells were handled following biosafety level-2 containment safety procedures defined in institutional biosafety protocols.

METHOD DETAILS

Purification of SINV-EEEV chimeric particles from mammalian cells

EEEV purification was optimized from a previously published alphavirus purification protocol (Zhang et al., 2011). Chimeric particles were grown in BHK-15 cells after infection of ~80% confluent cells at multiplicity of infection ~5 under biological safety level (BSL) 2 containment conditions. Modified Eagle Medium (MEM) growth medium supplemented with 2% fetal bovine serum (FBS) and 1X non-essential amino acids (NEAA) was collected after a post-infection incubation of 37°C for 16 h and clarified of cell debris by centrifugation. EEEV particles in the clarified growth medium were then subjected to precipitation with 14% (weight/volume) PEG-6000 and 4.6% (weight/volume) NaCl. The growth medium was then centrifuged at 2,500 x g at 4°C for 30 min to pellet EEEV particles. Further purification of EEEV particles was performed using a linear, continuous 0%–90% Optiprep gradient centrifuged at 247,000 x g for 1 h at 4°C. The purified EEEV particles were concentrated to 1-2 mg/ml of E2 protein for cryoEM.

Purification of SINV-EEEV chimeric particles from mosquito cells

Aedes albopictus mosquito C6/36 cells were cultured to ~90% confluence at 28-30°C in Dulbecco's Modified Eagle Medium (DMEM) supplemented with 10% FBS, 1X non-essential amino acids and 1X streptomycin-pencillin. EEEV infection was performed using virus diluted to an MOI of ~10 in DMEM followed by gentle rocking at 25°C for 1-2 hours. The EEEV containing medium was then removed, infected cells washed with 1X PBS and incubated for ~30 hours in the presence of DMEM (1X), FBS (2%) and NEAA (1X). Purification of EEEV from C6/36 cells was performed as described earlier for alphavirus produced in BHK-15 cells (Mukhopadhyay et al., 2006).

CryoEM data collection

Purified EEEV particles were flash frozen on lacey carbon EM grids in liquid ethane under BSL-2 containment conditions. For cryoEM analysis, movies of the frozen EEEV particles were recorded with the software Leginon (Carragher et al., 2000) using a Gatan K2 direct electron detector attached to a 300 keV Titan-Krios microscope using a dose of ~8 electrons per second.

CryoEM data processing

CryoEM movies (55 frames, 200 msec exposure per frame) were corrected for beam-induced motion using MotionCor2 (Zheng et al., 2017). Estimation of contrast transfer function (CTF) parameters was performed in CTFFIN4 (Rohou and Grigorieff, 2015). EEEV particles selected from micrographs using the software Appion (Lander et al., 2009) were subjected to reference-free 2D classification in RELION (Scheres, 2012) to identify a subset of homogeneous EEEV particles. A *de-novo* initial model from a small fraction of EEEV particles in *jspr* (Guo and Jiang, 2014) and was used for 2D alignment and 3D reconstruction by projection-matching. Refinement of particle center and orientation angles yielded a resolution of 8.5Å. Refinement of higher order parameters, i.e., defocus, beam tilt, astigmatism and anisotropic magnification improved the resolution of the cryoEM map to a resolution range of 3.5Å to 6.5Å, corresponding to an average resolution of 4.4Å. The resolution of the map was estimated corresponding to a gold-standard Fourier Shell Correlation (FSC) coefficient of 0.143 and the resolution range was calculated using the software ResMap (Kucukelbir et al., 2014; Rosenthal and Henderson, 2003; Scheres and Chen, 2012). The sharpening of the cryoEM map was performed in RELION using a B-factor of $-198Å^2$.

CryoEM model building

Homology models of EEEV E1 and E2 proteins and a structure of VEEV capsid CTD (Yang et al., 2015; Zhang et al., 2011) modified to EEEV capsid sequence were re-built in Rosetta (Alford et al., 2017) to improve fitting of the coordinates into the EEEV cryoEM map. The resulting coordinates were subjected to an iterative protocol of manual rebuilding in Coot (Emsley and Cowtan, 2004) and refinement against the cryoEM map in phenix.real-space.refine (Adams et al., 2010) to improve coordinate fitting by minimizing Ramachandran outliers (Table S2). Main-chain coordinates of residues Lys82-Gly99 of the capsid protein NTD directly preceding the capsid CTD were traced in the map in Coot and refined against the map in phenix.real-space.refine. The EEEV cryoEM map showed evidence of glycosylation of E1 and E2 ectodomains at Asn134 and Asn315 respectively. Carbohydrates were built in Coot. The quality of the protein models was evaluated using Molprobity (Chen et al., 2010).

Interpretation of EEEV-Fab complex structures

Five cryoEM structures of EEEV-Fab complexes were interpreted by fitting coordinates of E1-E2 ectodomains and an alphavirus Fab homolog, PDB ID 5ANY (Long et al., 2015) using EMfit assuming T = 4 quasi-symmetry in the icosahedral asymmetric unit. A quantity "sumf" that measures average electron density around fitted atoms was determined for E1-E2 ectodomains and Fabs fitted in each map assuming T = 4 icosahedral symmetry (Rossmann et al., 2001). The occupancies of fitted Fabs were determined by scaling Fab sumf values to averaged E1-E2 sumf values assuming that E1-E2 are present at 100% occupancy in each map. Roadmaps of Fab footprints on the EEEV E2 protein were generated using the program RIVEM (Xiao and Rossmann, 2007).

The orientation angles for Fabs with respect to the spike 3-fold axis were calculated as follows. Fabs consist of four domains: heavy (V_H) and light (V_L) chain variable domains and heavy (C_H) and light (C_L) chain constant domains. A quasi-2-fold axis relates the V_H-C_H domains to the V_L-C_L domains. The centers of mass of all four domains were determined individually. Then, an average value was

calculated for the center of mass for the V_H-V_L pair and another for the C_H-C_L pair. The Fab quasi-2-fold axis was represented as a vector extending from the average center of mass for the V_H-V_L pair to the C_H-C_L pair. Fab orientations were calculated between the Fab quasi-2-fold vector and the spike 3-fold vector.

Calculation of isoelectric points

Isoelectric points were calculated using the Expasy online server (Gasteiger et al., 2003). Sequence alignments were performed using Clustal-Omega (Sievers et al., 2011).The accession numbers for E1 and E2 sequences are: (EEEV), E1, NP_740648.1 and E2, ANB41727.1; (WEEV), E1, ACT75276.1 and E2, ABD57956.1; (VEEV), E1, AAD37000.1 and E2, AAU89534.1; (SINV), E1 NP_740677.1; (CHIKV), E1, AUS84459.1 and E2, ABN04188.1; (MAYV), E1, NP_579970.1 and E2, NP_579970.1, (RRV), E1, P08491 and E2, P08491. The SINV TR339 E2 sequence was obtained from (McKnight et al., 1996). Accession numbers for flavivirus E protein sequences are: Dengue virus serotype 1 (DENV1): GQ398255; Dengue virus serotype 2 (DENV2): NC_001474; Dengue virus serotype 3 (DENV3): EU081190; Dengue virus serotype 4 (DENV4): GQ398256; Japanese encephalitis virus (JEV): D90194; West Nile virus (WNV): DQ211652; Yellow fever virus Asibi (YFV_Asibi): AY640589; Zika virus (ZIKV_HPF): KJ776791.

Prediction of glycosylation sites

The sequences of E1 and E2 proteins listed above were analyzed for N-linked glycosylation motifs (Asn-X-Ser/Thr; X, any residue except Pro) (Gavel and von Heijne, 1990) using the NetNGlyc 1.0 software (http://www.cbs.dtu.dk/services/NetNGlyc/).

Quantitative glycan analysis by hydrophilic interaction chromatography-ultra performance liquid chromatography (HILIC-UPLC)

The E1 and E2 proteins of C6/36 and BHK-15 derived EEEV samples were separated by denaturing SDS-PAGE. Protein bands were excised for HILIC-UPLC. EEEV samples were inactivated by heating at 75°C for 5 minutes and then at 60°C for 15 minutes in the presence of 1% SDS for quantitative glycan analysis. Excised EEEV E1 and E2 gel bands were washed with alternate washes of acetonitrile and water before drying in a vacuum centrifuge. Bands were rehydrated with 100 μl of water and incubated with PNGase A and F at 37°C overnight. Released N-linked glycans were labeled by overnight incubation at 65°C with procainamide, using a labeling mixture of 110 mg/ml procainamide and 60 mg/ml sodium cyanoborohydrate in 70%DMSO and 30% glacial acetic acid. Labeled glycans were analyzed using a 2.1 mm × 10 mm Acquity BEH Glycan column (Waters) on a Waters Acquity H-Class UPLC instrument as performed in (Pritchard et al., 2015) with wavelengths of λ_{ex} = 310 and λ_{em} = 370. Endo H digestions of labeled glycans were used to quantify the abundance of oligomannose-type glycans, as previously described (Pritchard et al., 2015).

Flow cytometry analysis

Parental control Raji cells and Raji cells stably transfected with human DC-SIGN and L-SIGN were generously provided (Dan Littman, New York University School of Medicine) (Kwon et al., 2002). Cells were infected with GFP-expressing EEEV and SINV TR339 viruses exactly as described (Klimstra et al., 2003). At 18 hours post infection, cells were fixed in 4% paraformaldehyde and examined for GFP expression by flow cytometry.

mAb production

The generation, isolation and characterization of mouse mAbs has been described elsewhere (Kim et al., 2018). Fabs were generated from the mAbs by digestion with papain using a commercial Fab preparation kit from Thermo Fisher Scientfic following the manufacturer's instructions.

Focus reduction neutralization assay

Vero cells were seeded at 3 × 10^5 cells/well in a 96-well flat bottom plate 24 h prior to assay. mAbs or Fab fragments were diluted in DMEM supplemented with 5% fetal bovine serum (FBS), 100 U/ml penicillin, 100 μg/ml streptomycin and 10 mM HEPES and incubated with 100 FFU of EEEV for 1 h at 37°C. Virus-mAb or virus-Fab complex was added to the cell monolayer, and after a 1.5 h incubation, cells were overlaid with MEM containing 2% FBS and 1% (w/v) methylcellulose. Infection was allowed to proceed for 18 h and then fixed with 1% paraformaldehyde in phosphate buffered saline for 1 h. Plates were incubated with EEEV-10 (Kim et al., 2018) and subsequently with horseradish peroxidase-conjugated goat anti-human IgG. To visualize EEEV infected cell foci, TrueBlue peroxidase substrate was added to the plates and quantitated using an ImmunoSpot 5.0.37 macroanalyzer (Cellular Technologies Ltd). Neutralization curves were normalized to infected wells containing no mAbs and fitted using a nonlinear regression model. The HCV-specific mAb H77.39 was used as a negative control (Sabo et al., 2011).

Figures were prepared in Chimera (Pettersen et al., 2004), Coot (Emsley and Cowtan, 2004), PyMol and ESpript (http://espript.ibcp.fr/ESPript/ESPript/) (Gouet et al., 2003).

QUANTIFICATION AND STATISTICAL ANALYSIS

CryoEM reconstructions were performed using a "gold-standard" method (Scheres and Chen, 2012). Briefly, each particle data-set was divided randomly into two halves. Each half-set was reconstructed independently to minimize overfitting. The resolution of each

cryoEM map was determined using a Fourier shell correlation (FSC) coefficient of 0.143 between the two independently reconstructed half-maps (Rosenthal and Henderson, 2003). Data in resolution shells whose correlation was lower than 0.143 were omitted from the final maps. The reconstruction software *jspr* (Guo and Jiang, 2014) was used to calculate the FSC curves. Virus infection assays followed by flow cytometry were evaluated using two-way ANOVA with Tukey's multiple comparison test to identify the means whose values were significantly different from the remaining means. Neutralization experiments were performed twice, each time in duplicate. The neutralization curves report mean-values and standard deviations.

DATA AND SOFTWARE AVAILABILITY

All software and programs used in this paper for structure determination are available freely and are discussed in detail above. See the KEY RESOURCES table for the relevant references. The cryoEM maps and coordinates have been uploaded to the Electron Microscopy databank (EMDB) and Protein Data Bank (PDB) with the following accession codes: EEEV 4.4Å map (EMD-9280, PDB ID 6MX4), EEEV capsid N-terminal domain (EMD-9281, PDB ID 6MX7), EEEV complexed with Fab of EEEV-3 (EMD-9274, PDB ID 6MW9), EEEV complexed with Fab of EEEV-5 (EMD-9275, PDB ID 6MWC), EEEV complexed with Fab of EEEV-42 (EMD-9249, PDB ID 6MUI), EEEV complexed with Fab of EEEV-58 (EMD-9278, PDB ID 6MWV) and EEEV complexed with Fab of EEEV-69 (EMD-9279, PDB ID 6MWX).

Cell Reports, Volume *25*

Supplemental Information

Cryo-EM Structures of Eastern Equine Encephalitis Virus Reveal Mechanisms of Virus Disassembly and Antibody Neutralization

S. Saif Hasan, Chengqun Sun, Arthur S. Kim, Yasunori Watanabe, Chun-Liang Chen, Thomas Klose, Geeta Buda, Max Crispin, Michael S. Diamond, William B. Klimstra, and Michael G. Rossmann

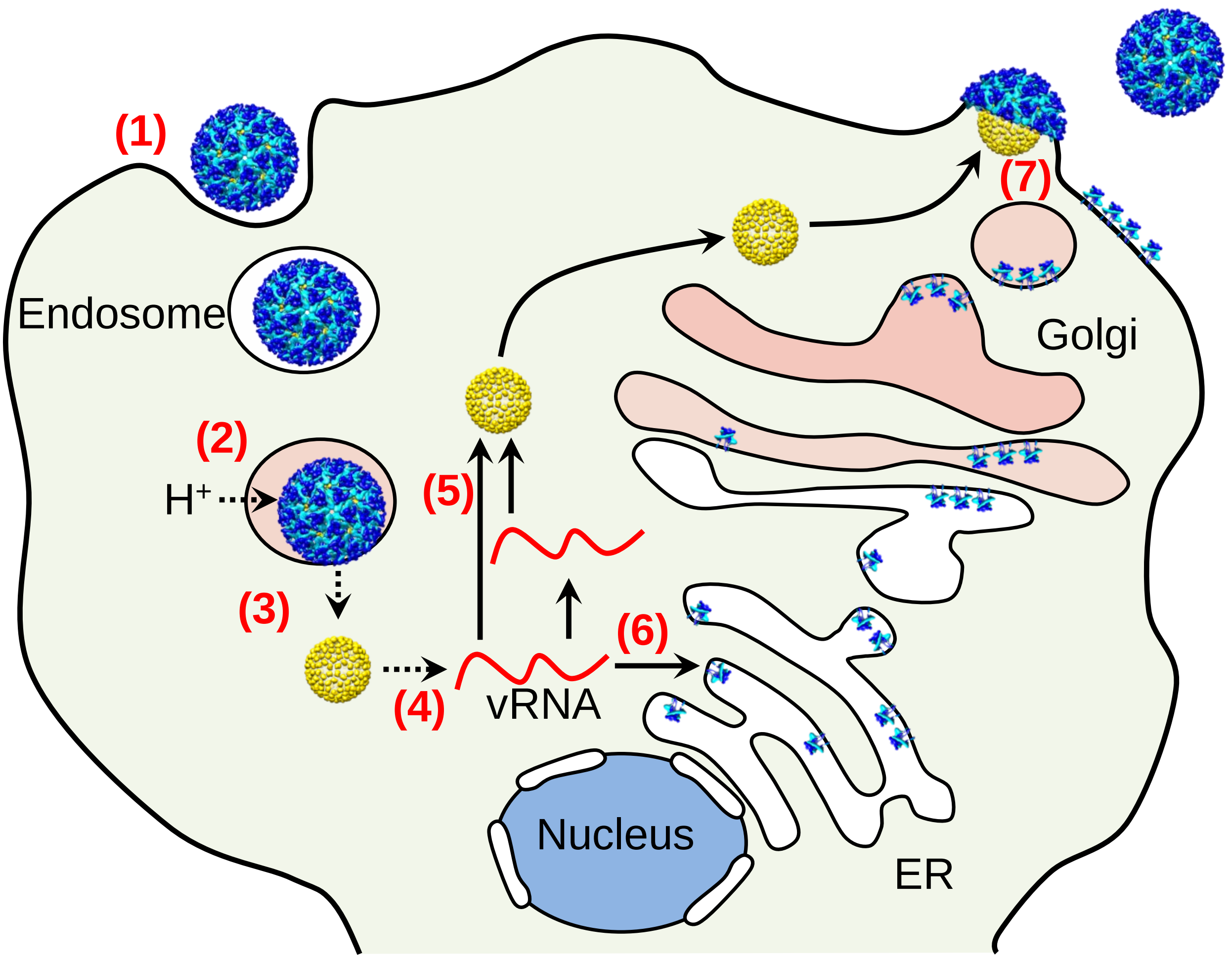

Figure S1. Life cycle of alphaviruses, Related to **Figure 1**. The steps involved in virus entry and disassembly are labelled in red from (**1**) to (**4**). The steps involved in progeny virus assembly and exit are labelled in red from (**5**) to (**7**). (**Step 1**) Alphavirus particles (blue, cyan) enter host cells by receptor-mediated endocytosis. (**Step 2**) The acidification of the endosome lumen (represented in pink) triggers fusion of the viral and endosomal membranes. (**Step 3**) The nucleocapsid core (yellow) is released into the cytosol. (**Step 4**) The core disintegrates to release the viral RNA genome (vRNA, red line). (**Step 5**) The synthesis of progeny RNA genome and nucleocapsid core takes place in the cytosol, whereas envelope protein synthesis involves the ER and Golgi network (**Step 6**). (**Step 7**) The assembly of progeny particles takes place at the plasma membrane.

Hasan *et al.*, Figure S2

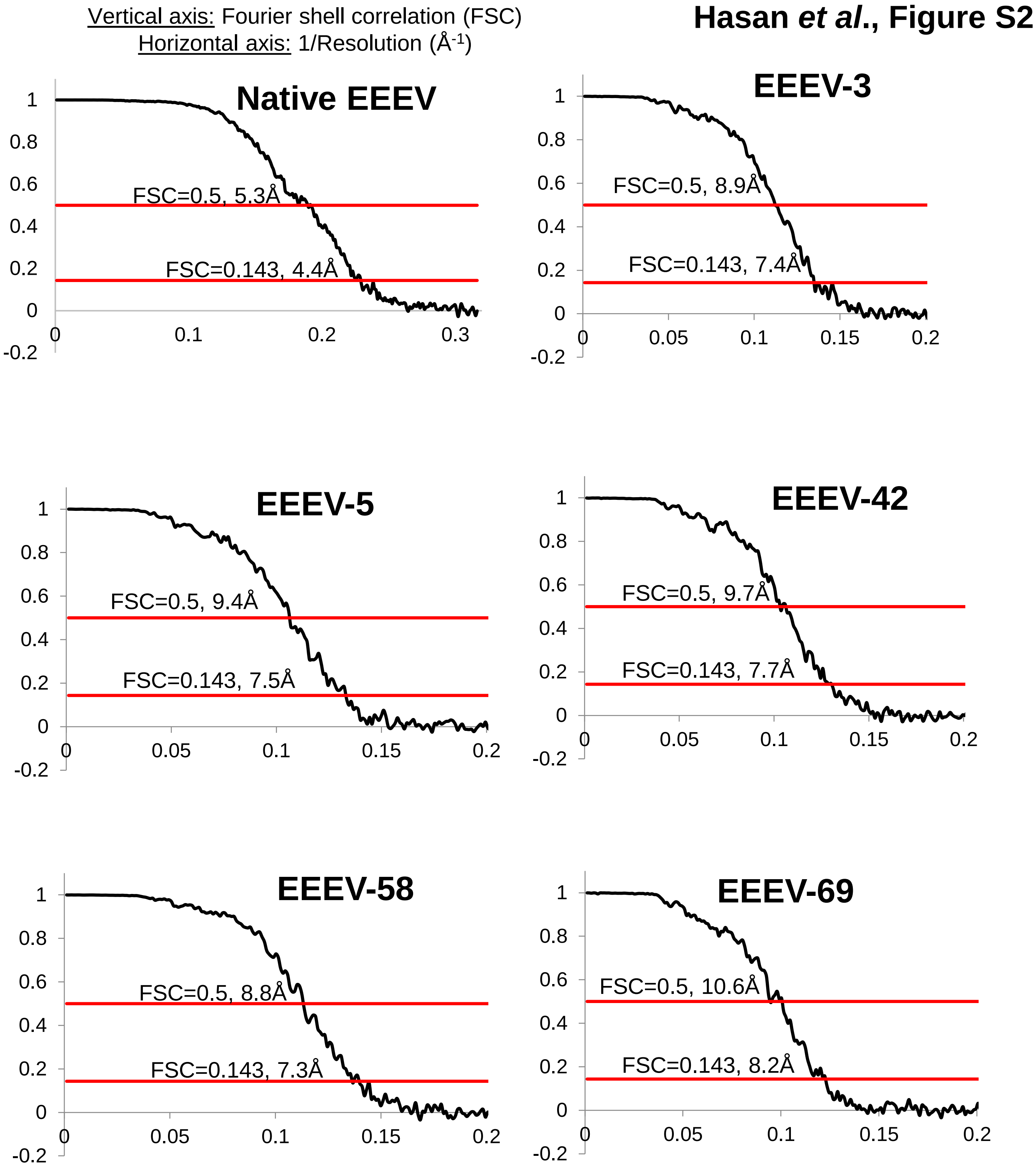

Figure S2. Fourier shell correlation (FSC) curves showing the resolutions of the native EEEV map and resolutions of five EEEV-Fab complex cryoEM maps, Related to **Figure 1**.

Statistics: Particles in each cryoEM reconstruction- native=30,806; EEEV+Fab3=8,416; EEEV+Fab5=6,583; EEEV+Fab42=4,733; EEEV+Fab58=7,335; EEEV+Fab69=5,964.

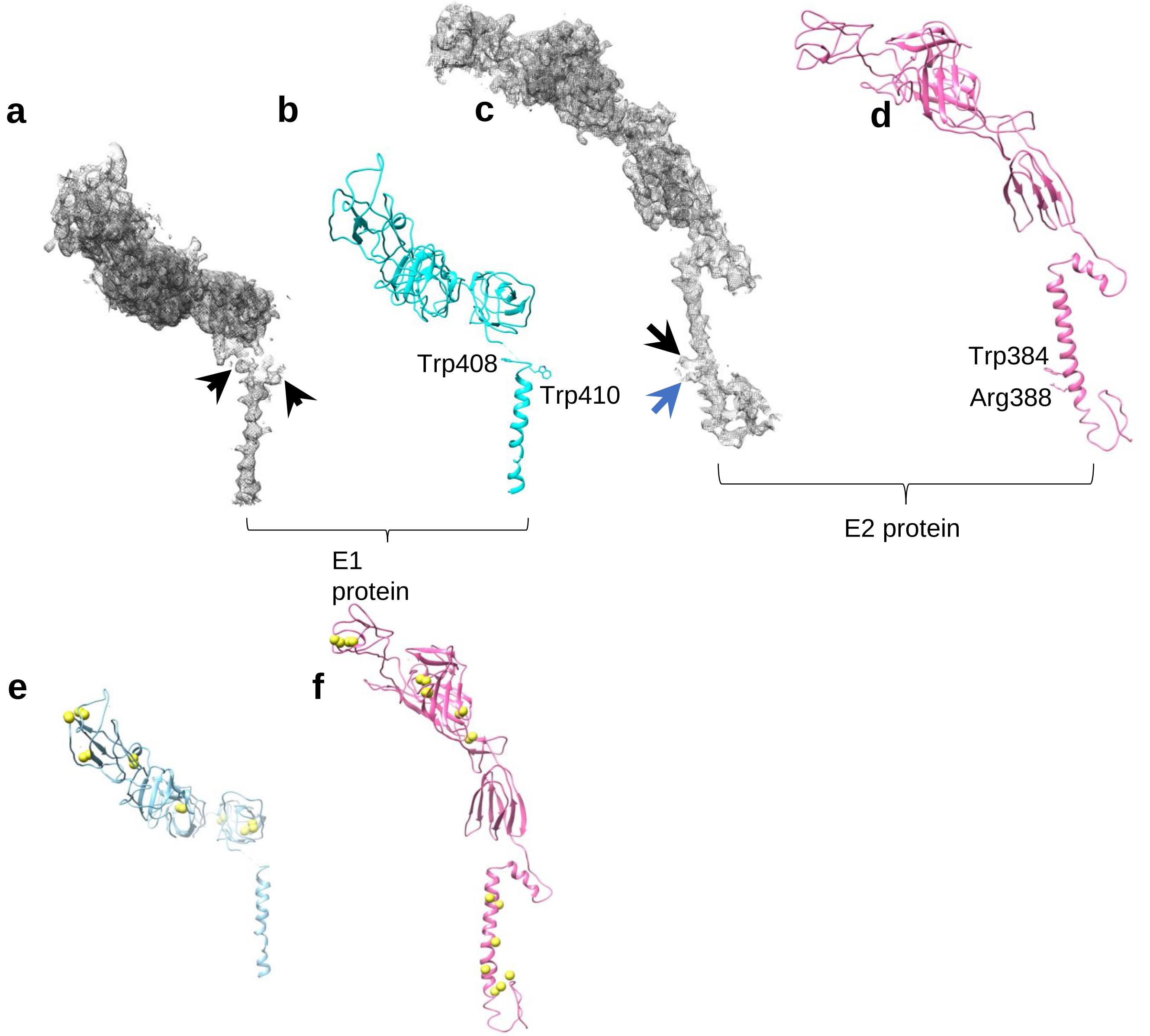

Figure S3. Structure of E1-E2 proteins, Related to **Figure 1**. (**a**) Map and (**b**) coordinates of the E1 protein, and (**c**) map and (**d**) coordinates of the E2 protein are shown. The black arrows point to aromatic residues whereas the blue arrow points to an Arg residue. (**e, f**) Cys residues in EEEV envelope proteins E1 and E2. The side-chain S-atoms of Cys residues are shown as yellow spheres. The Cys residues in E1 are restricted to the ecto-domain.

```
         1        10        20        30        40        50
1 EEEV   DLDTHFTQYKLARPYIADCPNCGHS.RCDSPIAIEEVRGDAHAGVIRIQTSAMFGL
2 VEEV   STEELFKEYKLTRPYMARCIRCAVG.SCHSPIAIEAVKSDGHDGYVRLQTSSQYGL
3 WEEV   PKRSITDDFTLTSPYLGFCPYCRHSAPCFSPIKIENVWDESDDGSIRIQVSAQFGY
4 CHIKV  STKDNFNVYKATRPYLAHCPDCGEGHSCHSPVALERIRNEATDGTLKIQVSLQIGI
5 MAYV   STANHFNAYKLTRPYVAYCADCGMGHSCHSPAMIENIQADATDGTLKIQFASQIGL
6 RRV    SVTEHFNVYKATRPYLAYCADCGDGYFCYSPVAIEKIRDEAPDGMLKIQVSAQIGL
7 SINV   ...SVTDDFTLTSPYLGTCSYCHHTEPCFSPVKIEQVWDEADDNTIRIQTSAQFGY

         60        70        80        90        100
1 EEEV   KTDGVD...L.AYMSFMNGKTQKSIKIDNLHVRTSAPCSLVSHHGYYILAQCPPGD
2 VEEV   DSSGNL...KGRTMRYDMHGTIKEIPLHQVSLHTSRPCHIVDGHGYFLLARCPAGD
3 WEEV   DQAGTADVTKFRYMSYDHDHDIKEDSVKKIAISTSGPCRRLGHKGYFLLAQCPPGD
4 CHIKV  KTDDSHDWTKLRYM...DNHMPADAERAGLFVRTSAPCTITGTMGHFILARCPKGE
5 MAYV   TKTDTHDHTKIRYA...EGHDIAEAARSTLKVHSSSECTVTGTMGHFILAKCPPGE
6 RRV    DKAGTHAHTKIRYM...AGHDVQESKRDSLRVYTSAACSIHGTMGHFIVAHCPPGD
7 SINV   DQSGAASANKYRYMSLEQDHTVKEGTMDDIKISTSGPCRRLSYKGYFLLAKCPPGD

         110       120       130       140       150       160
1 EEEV   TVTVGFHDGPN.RHTCTVAHKVEFRPVGREKYRHPPEHGVELPCNRYTHKRADQGH
2 VEEV   SITMEFKKDSV.THSCSVPYEVKFNPVGRELYTHPPEHGVEQACQVYAHDAQNRGA
3 WEEV   SVTVSITSGAS.ENSCTVEKKIRRKFVGREEYLFPPVHGKLVKCHVYDHLKETSAG
4 CHIKV  TLTVGFTDSRKISHSCTHPFHHDPPVIGREKFHSRPQHGKELPCSTYVQSTAATTE
5 MAYV   RISVSFVDSKNEHRTCRIAYHHEQRLIGRERFTVRPHHGIELPCTTYQLTTAETSE
6 RRV    YLKVSFEDADSHVKACKVQYKHDPLPVGREKFVVRPHFGVELPCTSYQLTTAPTDE
7 SINV   SVTVSIVSSNS.ATSCTLARKIKPKFVGREKYDLPPVHGKKIPCTVYDRLKETTAG

             170       180       190       200       210
1 EEEV   YVEMHQPGLVADHSLLSIHSAKVKITVPSGAQVKYYCKCPD..VREGITSSDHTTT
2 VEEV   YVEMHLPGSEVDSSLVSLSGSSVTVTPPVGTSALVECECGGTKISETINKTKQFSQ
3 WEEV   YITMHRPGPHAYKSYLEEASGEVYIKPPSGKNVTYECKCGDYSTGI.VSTRTKMNG
4 CHIKV  EIEVHMPPDTPDRTLMSQQSGNVKITV.NGQTVRYKCNCGGSNEGL..TTTDKVIN
5 MAYV   EIDMHMPPDIPDRTILSQQSGNVKITV.NGRTVRYSSSCGSQAVGT..TTTDKTIN
6 RRV    EIDMHTPPDIPDRTLLSQTAGNVKITA.GGRTIRYNCTCGRDNVGT..TSTDKTIN
7 SINV   YITMHRPGPHAYTSYLEESSGKVYAKPPSGKNITYECKCGDYKTGT.VSTRTEITG

           220       230       240       250       260       270
1 EEEV   CTDVKQCRAYLIDNKKWVYNSGRLPRGEG.DTFKGKLHVPFVPVKAKCIATLAPEP
2 VEEV   CTKKEQCRAYRLQNDKWVYISDKLPKAAG.ATLKGKLHVPFLLADGKCTVPLAPEP
3 WEEV   CTKAKQCIAYKSDQTKWVYNSPDLIRHTD.HSVQGKLHIPFRLTPTFCPVPLAHTP
4 CHIKV  NCKVDQCHAAVTNHKKWQYNSPLVPRNAELGDRKGKIHIPFPLANVTCRVPKARNP
5 MAYV   SCTVDKCQAYVTSHTKWQFNSPFVPRRMQ.AERKGKVHIPFPLINTTCRVPLAPEA
6 RRV    TCKIDQCHAAVTSHDKWQFTSPFVPRADQ.TARRGKVHVPFPLTNVTCRVPLARAP
7 SINV   CTAIKQCVAYKSDQTKWVFNSPDLIRHDD.HTAQGKLHLPFKLIPSTCMVPVAHAP

             280       290       300       310   ★   320
1 EEEV   LVEHKHRTLILHLHPDHPTLLTTRSLGSDANPTRQWIERPTTVNFTVTGEGLEYTW
2 VEEV   MITFGFRSVSLKLHPKNPTYLTTRQLADEPHYTHELISEPAVRNFTVTGKGWEFVW
3 WEEV   TVTKWFKGITLHLTATRPTLLTTRKLGLRADATAEWITGTTSRNFSVGREGLEYVW
4 CHIKV  TVTYGKNQVIMLLYPDHPTLLSYRNMGEEPNYQEEWVMHKKEVVLTVPTEGLEVTW
5 MAYV   LVRSGKREATLSLHPIHPTLLSYRTFGAERVFDEQWITAQTEVTIPVPVEGVEYQW
6 RRV    DVTYGKKEVTLRLHPDHPTLFSYRSLGAEPHPYEEWVDKFSERIIPVTEEGIEYQW
7 SINV   NVIHGFKHISLQLDTDHLTLLTTRRLGANPEPTTEWIVGKTVRNFTVDRDGLEYIW

         330       340       350       360       370       380
1 EEEV   GNHPPKRVWAQESGEGNPHGWPHEVVVYYYNRYPLTTIIGLCTCVAIIMVSCVTSV
2 VEEV   GNHPPKRFWAQETAPGNPHGLPHEVITHYYHRYPMSTILGLSICAAIATVSVAAST
3 WEEV   GNHEPVRVWAQESAPGDPHGWPHEIIIHYYHRHPVYTVIVLCGVALAILVGIASSA
4 CHIKV  GNNEPYKYWPQLSTNGTAHGHPHEIILYYYELYPTMTVVVVSVATFILLSMVGMAA
5 MAYV   GNHKPQRFVVALTTEGKAHGWPHEIIEYYYGLHPTTTIVVVIRVSVVVLLSFAASV
6 RRV    GNNPPVRLWAQLTTEGKPHGWPHEIIQYYYGLYPAATIAAVSGASLMALLTLAATC
7 SINV   GNHEPVRVYAQESAPGDPHGWPHEIVQHYYHRHPVYTILAVASATVAMMIGVTVAV

             390       400       410       420
1 EEEV   WLLCRTRNLCITPYKLAPNAQVPILLALLCCIKPTRA
2 VEEV   WLFCRSRVACLTPYRLTPNARIPFCLAVLCCARTARA
3 WEEV   ACIAKARRDCLTPYALAPNATVPTALAVLCCIRPTNA
4 CHIKV  GMCMCARRRCITPYELTPGATVPFLLSLICCIRTAKA
5 MAYV   YMCVVARTKCLTPYALTPGAVVPVTIGVLCCAPKAHA
6 RRV    CMLATARRKCLTPYALTPGAVVPLTLGLLCCAPRANA
7 SINV   LCACKARRECLTPYALAPNAVIPTSLALLCCVRSANA
```

Figure S4. **Multiple sequence alignment of alphavirus E2 protein sequences,** Related to **Figure 2, 5, and 6**. Glycosylation motif is shown in a blue box. Sequence conservation color code: red, complete; yellow, partial; white, none.

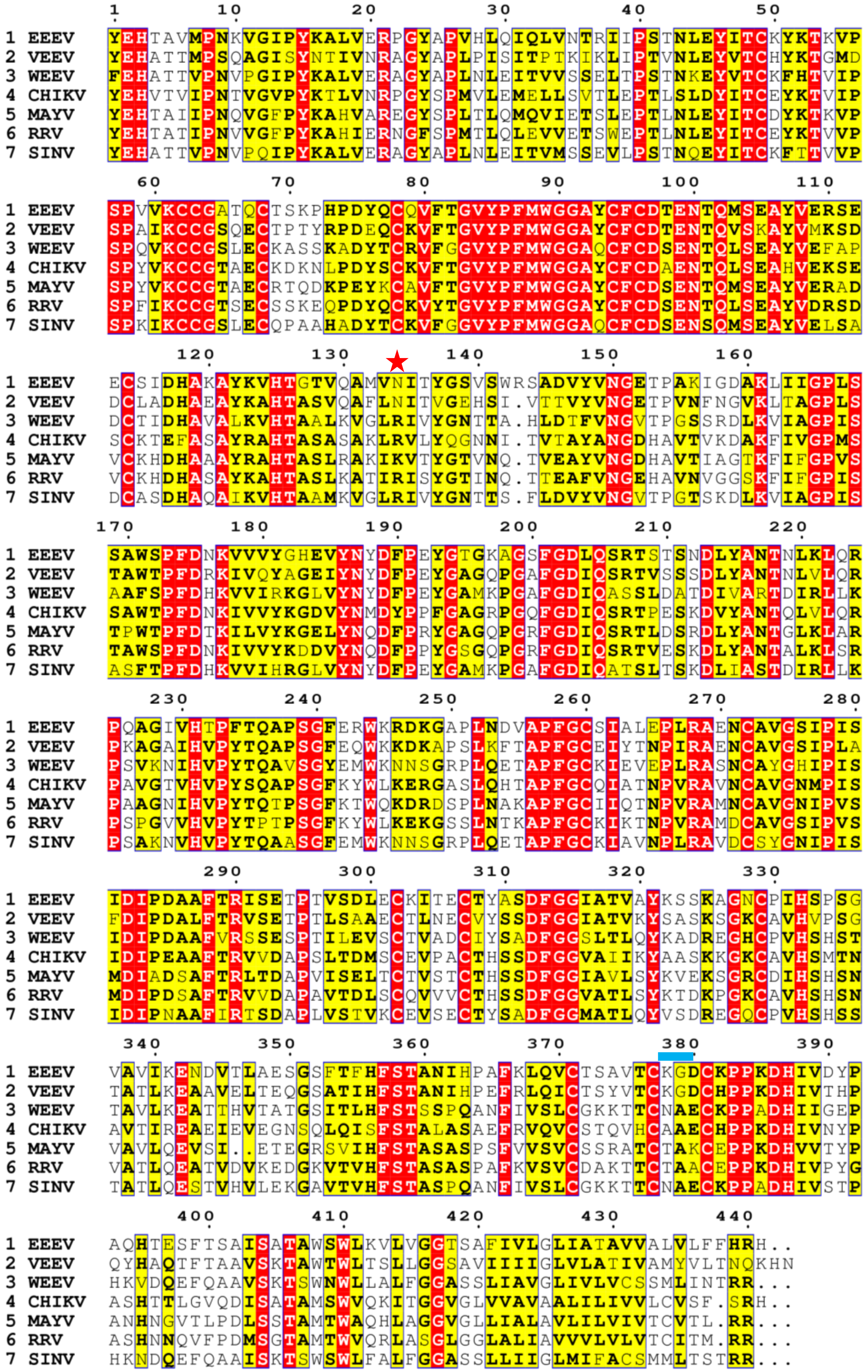

Figure S5. Multiple sequence alignment of alphavirus E1 protein sequences, Related to **Figure 2, and 6**. Glycosylation motif is shown in a blue box. Sequence conservation color code: red, complete; yellow, partial; white, none.

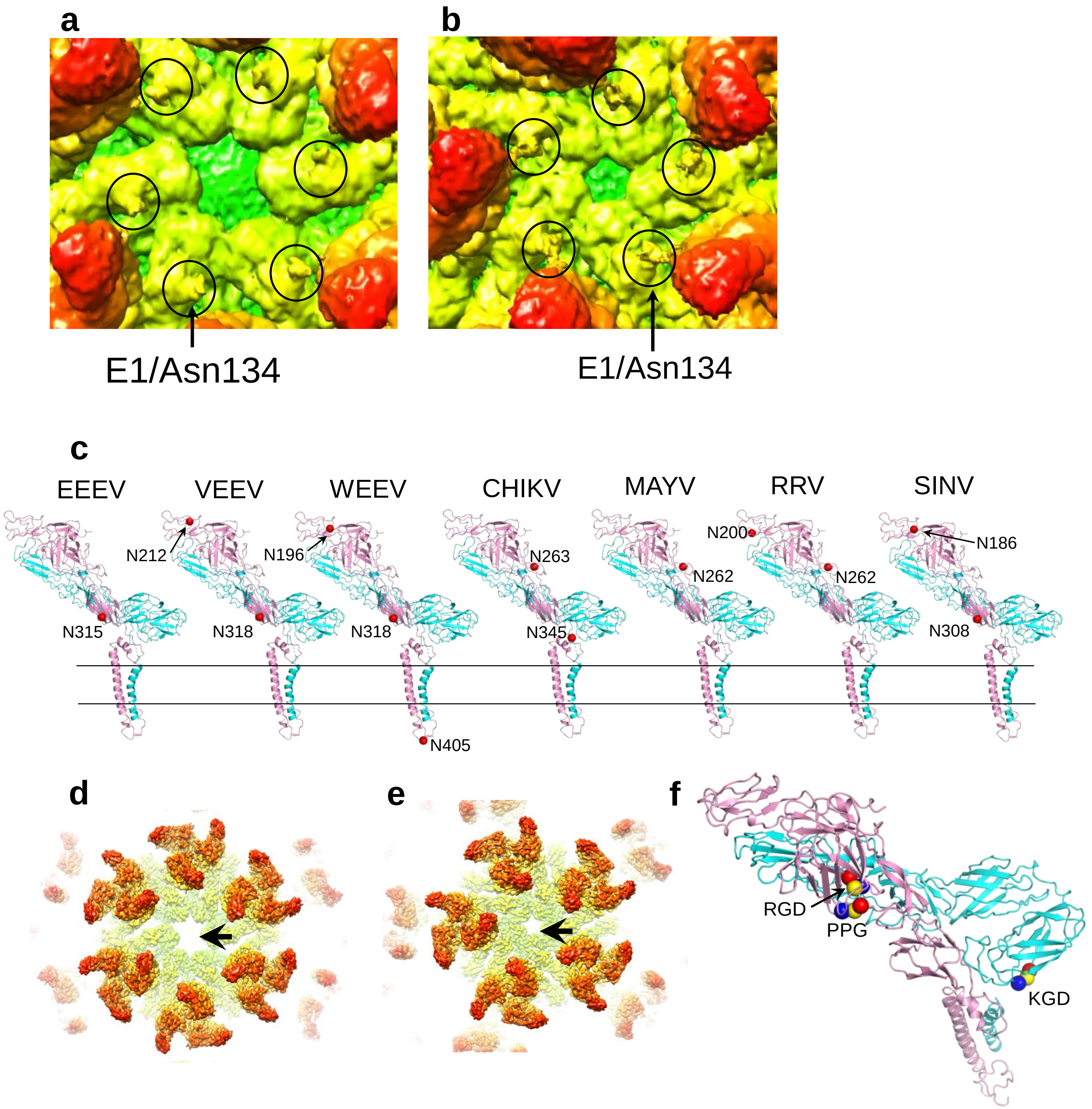

Figure S6. Envelope protein glycosylation and receptor binding sites, Related to **Figure 2**. Symmetry-related carbohydrate sites are shown near the (a) icosahedral 2-fold and (b) 5-fold vertices. The black ovals highlight symmetry-related carbohydrate sites. The map is unsharpened and colored according to the radial scale in Figure 1a. (**c**) Glycosylation sites in alphavirus receptor-binding E2 protein. The Cα-atom of Asn residue in a glycosylation motif is shown as a red sphere modeled in the EEEV E1-E2 dimer structure. E1, cyan; E2, pink. (**d, e, f**) Putative receptor binding sites in EEEV. Exposed viral membrane at the icosahedral (**d**) 2-fold and (e) 5-fold vertices for TIM1 interactions. Black arrows point to the exposed membrane. The map is colored according to the radial scale in Figure 1a. (**f**) Integrin binding motifs in EEEV E1-E2 dimer. Each motif consists of three amino acids as described here and in the main text. The C-atoms of each amino acid in the motif are shown as spheres (red, yellow, blue from N to C terminus). EEEV E2 motifs: RGD (Arg37-Gly38-Asp39) and PPG (Pro104-Pro105-Gly106). E1 motif: KGD motif (Lys378-Gly379-Asp380). E1, cyan; E2, pink.

Hasan *et al.*, Figure S7

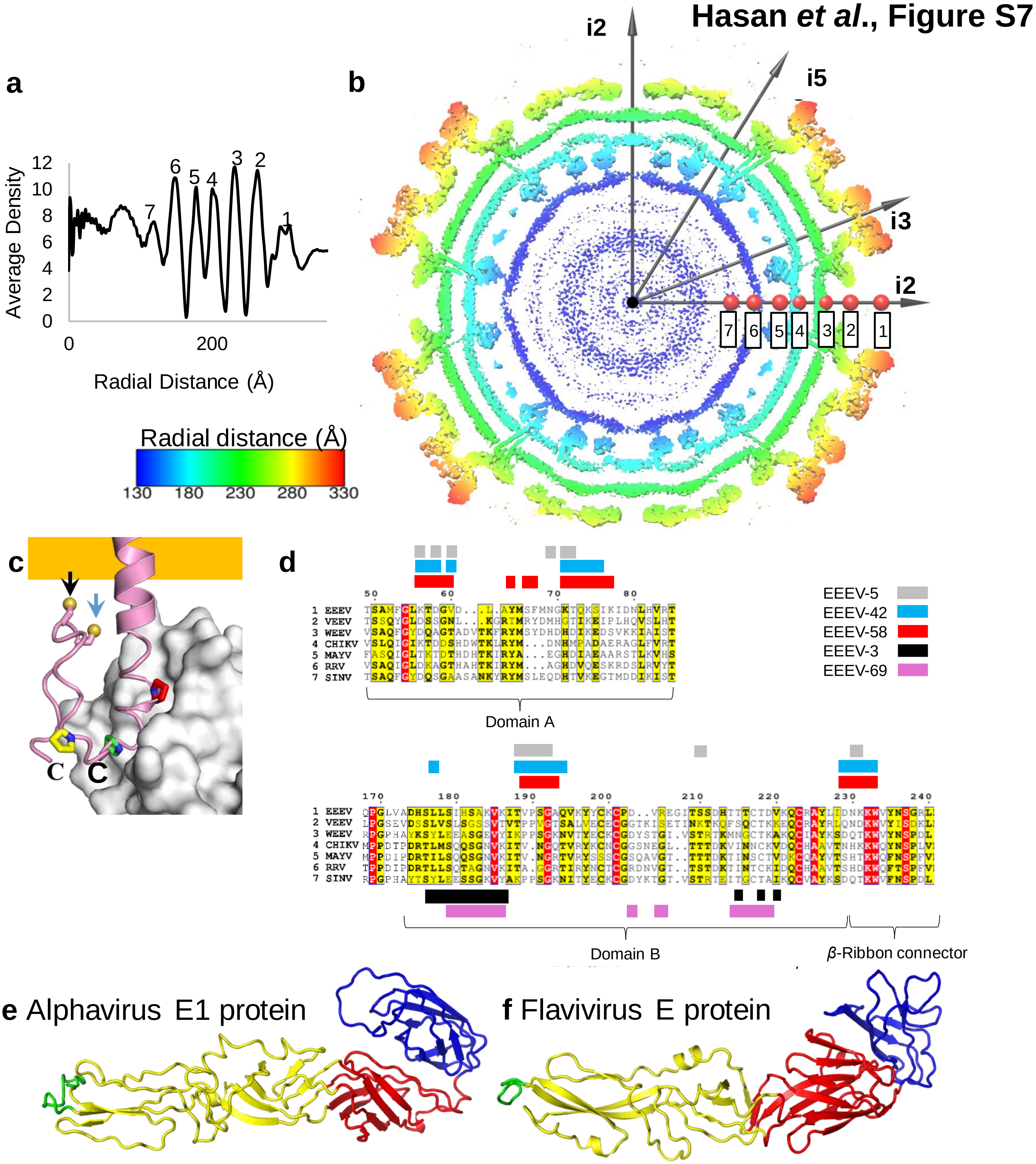

Figure S7. Structural features of EEEV, Related to **Figures 3, 4, 5, and 6**. (**a, b**) Radial density distribution of EEEV cryoEM map. (**a**) Trace of radial density. (**b**) Peak assignment: E2 ecto-domain (peak 1, r=300Å), base of E1-E2 spike (peak 2, r=263Å), outer-leaflet lipid bilayer head-groups (peak 3, r=232Å), inner-leaflet lipid bilayer head-groups (peak 4, r=200Å), capsid chymotrypsin-like C-terminal domain (peak 5, r=178Å), capsid RNA-binding N-terminal domain (peak 6, r=147Å), unidentified density (peak 7, r=119 Å). The peaks of radial density distribution are mapped as red spheres on a radially colored section of the EEEV cryoEM map. Icosahedral axes are shown as arrows. (**c**) Capsid-E2 interactions. The capsid C-terminal chymotrypsin-like domain (white surface) accommodates a turn at the E2 C-terminus (pink). Conserved E2 residues critical for the structure of the turn are shown (red, Pro396; green, Pro401; yellow, Pro406. The black and blue arrows point to the side-chains of Cys413 and Cys414 respectively, which face the membrane. (**d**) Footprints of individual Fabs are color-coded according to the key on the right. Red indicates complete conservation, yellow partial conservation and white no conservation of E2 residues. (**e**) EEEV E1 ecto-domain and (**f**) ZIKV E ecto-domain (PDB 5IRE). Color code: domain I, red; domain II, yellow; domain III, blue; fusion peptide, green.

SUPPLEMENTARY TABLES

Table S1. Summary of cryoEM data collection and processing, Related to **Figure 1**.

			EEEV-Fab Complex				
		EEEV	EEEV-3	EEEV-5	EEEV-42	EEEV-58	EEEV-69
Data Deposition	EMDB	9280	9274	9275	9249	9278	9279
	PDB ID	6MX4	6MW9	6MWC	6MUI	6MWV	6MWX
Microscope Settings	Dose (e^-/Å^2)	31	31	31	31	31	31
	Magnification (X)	18,000	18,000	18,000	18,000	18,000	18,000
	Pixel size (Å)	1.58	1.62	1.62	1.62	1.62	1.62
	Defocus range (µm)	-0.5 to -3.5	-1.5 to -5.0	-1.5 to -5.0	-1.0 to -3.5	-1.5 to -5.0	-1.0 to -3.5
Data	#Micrographs	2,416	937	765	570	900	579
	#Boxed particles	72,833	24,202	16,583	7,318	15,679	7,574
	#Particles after 2D classification	30,806	8,416	6,583	4,733	7,335	5,964
Resolution (Å)	FSC=0.5	5.3	8.9	9.4	9.7	8.8	10.6
	FSC=0.143	4.4	7.4	7.5	7.7	7.3	8.2

Table S2. Refinement statistics of E1-E2-capsid coordinates assuming four copies each of protein in the asymmetric unit, Related to **Figure 1**.

Ramachandran (favored+allowed)	99.6%
Ramchandran (outliers)	0.4%
Rotamers (favored + allowed)	100%
Rotamers (outliers)	0.0%
Cβ deviations	0
Molprobity all atom clash-score	11.0

Table S3. Isoelectric points of EEEV envelope proteins, Related to **Figure 5**.

	E1	E2
Full length	6.6	8.8
Ecto-domain	6.2	8.6
Binding site	4.6	10.2

Table S4. Isoelectric points (pI) of alphavirus envelope protein ecto-domains, Related to **Figure 5**.

Disease	Virus	E1 ecto-domain	E2 ecto-domain
		pI	pI
Encephalitis	EEEV	6.2	8.6
	WEEV	6.0	8.9
	VEEV	6.1	8.3
Arthritis	SINV	6.0	7.9
	CHIKV	6.1	7.6
	MAYV	6.7	8.0
	RRV	6.2	6.8

Table S5. Sumf values of E1-E2 domains in EEEV-Fab complex cryoEM maps, Related to **Figures 6** and **7**. The sumf values were averaged for four symmetry-related positions within the T=4 icosahedral asymmetric.

	Fab	E1 ecto-domain			E2 ecto-domain			
		I	II	III	β-Ribbon	A	B	C
Domain A	5	38.3	42.2	38.8	35.2	32.4	40.7	26.8
	42	38.7	39.9	41.1	32.5	31.2	41.0	28.7
	58	37.7	42.1	41.0	33.3	31.5	42.0	26.9
Domain B	3	36.4	41.3	40.6	37.9	35.4	42.6	26.9
	69	37.7	42.1	41.0	33.3	31.5	42.0	26.9

Table S6. Fabs: Occupancy and orientation, Related to **Figures 6** and **7**.

E2 Domain	A				A				A			
Fab	5				42				58			
Position	i1	q1	q2	q3	i1	q1	q2	q3	i1	q1	q2	q3
E1 ecto-domain	40.1	40.4	40.3	39.6	39.3	39.4	39.2	40.4	39.9	40.0	41.6	40.3
E2 ecto-domain	35.7	36.8	37.1	36.1	35.1	35.7	35.1	33.5	35.4	36.8	36.3	34.4
Fab	17.2	19.5	16.3	16.6	28.4	27.6	26.4	27.6	37.4	36.9	35.8	36.3
Occupancy (%)	45.4	50.5	42.1	43.9	76.3	73.5	71.1	74.7	99.3	96.1	91.9	97.2
Avg. Occupancy (%)	45.4				74.7				97.2			
Angle (°)	14.5				31.3				32.0			

E2 Domain	B				B			
Fab	3				69			
Position	i1	q1	q2	q3	i1	q1	q2	q3
E1 ecto-domain	38.8	39.2	39.7	40.8	41.6	41.4	40.4	41.7
E2 ecto-domain	37.3	38.4	39.2	38.2	40.7	40.9	38.6	38.1
Fab	34.5	34.2	34.1	34.1	37.1	34.0	33.6	34.5
Occupancy (%)	90.7	88.1	86.4	86.3	90.2	82.6	85.1	86.5
Avg. Occupancy (%)	88.8				87.5			
Angle (°)	21.1				52.0			

Table S7. Isoelectric points (pI) of flavivirus E protein ecto-domains, Related to **Figure 5**.

Flavivirus	pI
DENV1	6.5
DENV2	6.8
DENV3	6.1
DENV4	6.5
JEV	6.4
WNV	6.4
YFV_Asibi	5.8
ZIKV_HPF	6.1
